# Incidence and mortality of kidney cancer: temporal patterns and global trends in 39 countries

**DOI:** 10.1038/s41598-017-15922-4

**Published:** 2017-11-16

**Authors:** Martin C. S. Wong, William B. Goggins, Benjamin H. K. Yip, Franklin D. H. Fung, Colette Leung, Yuan Fang, Samuel Y. S. Wong, C. F. Ng

**Affiliations:** 1 School of Public Health and Primary Care, Faculty of Medicine, The Chinese University of Hong Kong. Prince of Wales Hospital, Shatin, New Territories Hong Kong; 2 Family Medicine and Primary Health Care, Hospital Authority, Shatin, New Territories Hong Kong; 3 Division of Urology, Department of Surgery, Faculty of Medicine, The Chinese University of Hong Kong, Shatin, New Territories Hong Kong

## Abstract

We tested the hypotheses that kidney cancer incidence was increasing globally whilst its mortality was reducing; and its incidence was positively correlated with country-specific socioeconomic development. The incidence and mortality figures of each country were projected to 2030. Data on age-standardized incidence/mortality rates were retrieved from the GLOBOCAN in 2012. Temporal patterns were examined for 39 countries from the Cancer Incidence in Five Continents volumes I-X and other national registries. We evaluated the correlation between the incidence/mortality rates and Human Development Index (HDI)/Gross Domestic Product (GDP]). The average annual percent change of its incidence and mortality in the most recent 10 years was obtained from joinpoint regression. The highest incidence rates were observed in Eastern Europe and North America, while its mortality rates were the highest in European countries. Incidence was positively correlated with HDI and GDP per capita. Many countries experienced incidence rise over the most recent 10 years, and a substantial reduction in mortality rates was observed for a significant number of countries, yet increases in mortality rates were observed in Eastern Europe. By 2030, Brazil and Ecuador may have the greatest rise in incidence both in men and women, which requires urgent need for planning healthcare resources.

## Introduction

Kidney cancer was the seventh most common malignancy and accounted for 3.3% of all newly diagnosed cancer in 2012^1^. Renal cell carcinoma (RCC) constitutes approximately 90–95% of all kidney neoplasms^2^, and 25–30% of all patients had metastatic disease upon its diagnosis^3^. The estimated economic burden of metastatic RCC was $1.6 billion (2006 USD) in selected countries^3^. It is a rapidly evolving area of solid tumor oncology^4^. Recent studies showed that several European regions reported particularly high incidence rates^5^.

Recognized risk factors for RCC include cigarette smoking, obesity and hypertension^6^. Accumulating evidence suggests an etiologic role for physical inactivity, alcohol consumption, high parity among women, and occupational exposure to trichloroethylene. Many of these risk factors are amenable to lifestyle modifications and there exists a strong prospect for intervention. Previous studies examining its global trends were based on figures in the 1990s to early 2000s, did not make direct comparisons among countries, or focused on selected regions^7–9^. Based on existing literature^10,11^, it is worthwhile to analyze if the patterns and temporal trends of kidney cancer could quantify geographical variations, and identify modifiable factors that might have contributed to trend changes^7^. Projected estimates of its incidence and mortality are particularly useful to inform healthcare planning and priority setting.

There are two important knowledge gaps in kidney cancer research. Firstly, previous literature shows that the highest incidence occurs in more developed countries, and the recent decades witness increasing affluence and technological advancement especially in more developed nations. Temporal trends and projected changes in its future incidence and mortality are largely unaddressed. In addition, the role of socioeconomic status in RCC was still inconclusive when their associations were examined globally^12^. A recent study showed that among 9,623 patients with metastatic RCC at diagnosis, most were uninsured; residing in underprivileged regions, and were poorly educated^13,14^; but sporadic evidence suggested the contrary^15^. These findings highlight the need for a worldwide, across-country analysis. Furthermore, there have been no studies that have attempted to project the future trends of kidney cancer based on current epidemiological data.

Previous studies in the past decade showed that the number of new cases of kidney cancer increased and its mortality declined in populations of different ethnicities^7–11^. Hence, this study tested the *a priori* hypothesis that the temporal trends of its incidence increased and its mortality decreased. Also, we sought to test the hypothesis that its global incidence was positively correlated with country-specific socioeconomic development, and projected the incidence/mortality figures to 2020 and 2030. To standardize the methodology across published literature, we adopted the same analysis plan as reported in previous similar studies on prostate, colorectal, liver and pancreatic cancer^16–19^.

## Methods

### Data Source

We retrieved the incidence and mortality figures for kidney cancer in 2012 from the GLOBOCAN database for 167 countries^1^, excluding cancer of the renal pelvis and ureter. For all countries, data were matched with their Human Development Index (HDI) and Gross Domestic Product (GPD) per capita in the same year based on the United Nations Human Development Report^20^, which highlights the progress on human development over the past quarter century by reporting the different statistical indexes. HDI is a composite index of life expectancy, education period, and income per capita indicators^20^. For incidence figures, we extracted data from the Cancer Incidence in Five Continents series Volumes I-X^21^, which provided high-quality incidence statistics of cancer documented by local registries worldwide.

To acquire incidence data for more recent years, we also utilized publicly available information from the European Union Registration (EUREG)^22^, National Cancer Institute^23^, Nordic Cancer Registries^24^, Australian Cancer Incidence and Mortality Books^25^ and the Ministry of Health of New Zealand^26^. We used GLOBOCAN to report the latest incidence and mortality figures (2012) that are available for all countries, as well as the correlation between the socioeconomic indices and age-standardized rates of incidence/mortality. For evaluation of temporal trends of incidence and mortality in each country, we used the various databases highlighted in Table 1 to evaluate the Average Annual Percent Change (AAPC) by joinpoint regression analysis^22–26^. The incidence data were retrieved according to the International Classification of Diseases (ICD-10, C64; ICD-9-CM 189.0).

**Table 1:** Data source for the age-standardized incidence and mortality rates of kidney cancer.

	Incidence	Mortality
Austria	EUREG (1990–2009)	EUREG (1990–2009)
Croatia	CI5 (1988–2007)	EUREG (2000–2007)
Czech Republic	CI5 (1983–2007)	EUREG (1998–2007)
Denmark	NORDCAN (2004–2013)	NORDCAN (2004–2013)
Estonia	CI5 (1968–2007)	EUREG (1994–2010)
Finland	NORDCAN (2004–2013)	NORDCAN (2004–2013)
France	CI5 (1988–2007)	n/a
Germany	CI5 (1970–2007)	n/a
Iceland	NORDCAN (2004–2013)	NORDCAN (2003–2012)
Italy	CI5 (1993–2007)	n/a
Latvia	CI5 (1988–2007)	EUREG (2003–2007)
Lithuania	CI5 (1978–2007)	n/a
Malta	EUREG (1994–2009)	EUREG (1995–2010)
Netherlands	CI5 (1989–2007)	EUREG (1989–2007)
Norway	NORDCAN (2004–2013)	NORDCAN (2004–2013)
Poland	CI5 (1978–2006)	n/a
Slovakia	CI5 (1968–2007)	EUREG (1978–2010)
Slovenia	CI5 (1963–2007)	EUREG (1985–2008)
Spain	CI5 (1993–2007)	n/a
Sweden	NORDCAN (2004–2013)	NORDCAN (2004–2013)
Switzerland	CI5 (1993–2007)	n/a
United Kingdom	CI5 (1993–2007)	n/a
Australia	AIHW (1982–2012)	AIHW (1968–2013)
New Zealand	New Zealand (1960–2012)	New Zealand (1960–2012)
Bulgaria	EUREG (1993–2007)	EUREG (1993–2008)
Ireland	EUREG (1994–2009)	EUREG (1994–2010)
Brazil	CI5 (1988–2007)	n/a
Colombia	CI5 (1983–2007)	n/a
Costa Rica	CI5 (1980–2007)	n/a
Ecuador	CI5 (1985–2007)	n/a
Canada	CI5 (1978–2007)	n/a
USA	NCI (1975–2013)	NCI (1975–2013)
USA White	NCI (1975–2013)	NCI (1975–2013)
USA Black	NCI (1975–2013)	NCI (1975–2013)
India	CI5 (1993–2007)	n/a
Israel	CI5 (1963–2007)	n/a
Japan	CI5 (1988–2007)	n/a
Philippines	CI5 (1983–2007)	n/a
Singapore	CI5 (1968–2007)	n/a
Thailand	CI5 (1993–2007)	n/a
China	Hospital Authority (1983–2013)	Hospital Authority (1983–2013)

n/a: not available; AIHW: Australian Cancer Incidence and Mortality Books^25^; CI5: Cancer Incidence in Five Continents V^21^; EUREG: European Union Registration^22^; NCI: National Cancer Institute^23^; New Zealand: the Ministry of Health of New Zealand^26^; NORDCAN: Nordic Cancer Registries^24^.

For mortality data, we made reference to the various national databases^22–26^, where the primary data source originated from death certificates. These data were categorized based on the ICD 9^th^ or ICD 10^th^, according to the calendar year where the coding are available specific to each country. The WHO mortality database was not used as it does not consist of “kidney cancer” as a cause of death. Table 1 showed a more detailed description of the data sources and calendar years included for the present analysis. We adopted age-standardized rate (ASR) using the world standard population^27^. More developed regions include Europe, Northern America, Australia/New Zealand and Japan, whilst others are less developed regions^1^.

### Statistical Analysis

We used joinpoint regression analysis to study the incidence/mortality trends^28^. A series of joined straight lines was fit to the ASR trend^28^. We performed logarithmic transformation of the ASRs and computed the standard errors adopting binomial approximation. A maximum number of three joinpoints were used as analysis options, and we evaluated the AAPC and the respective 95% confidence intervals (C.I.) for data available in the most recent 10 years. The AAPC was computerized as a geometrically weighted average of the generated APCs by the joinpoint trend analysis software. Their weights were equivalent to the length of each segment within the specified time interval^29^. We extracted all available global data for the incidence and mortality trends. We selected the most recent 10 years as the timeframe for examining temporal trend changes, as was commonly adopted in previous studies on global epidemiology of other cancers^16,17,30^. All AAPCs with their 95% C.I. lying above and below zero, respectively, were regarded as increasing and decreasing trends. AAPCs with 95% C.Is overlapping with zero was considered as stable trends^16,17,30^. AAPCs with 95% C.Is overlapping with zero was considered as stable trends. We plotted the ASRs of incidence and mortality against the HDI and GDP per capita, respectively, for each country. The HDI was defined as low (≤0.534), medium (0.534–0.710), high (0.710–0.796) and very high (>0.796)^20^. Logarithmic transformation of the ASR of incidence and mortality was applied in their correlations with HDI and GDP per capita as their associations were non-linear. Their correlation coefficients were evaluated. Also, we estimated the percent change in incidence and mortality by 2020 and 2030 when compared to the latest published figures based on the AAPC – a statistical technique employed by Bailey and colleagues in *JAMA Surgery* ^30^. The predicted incidence/mortality rates were assumed to change at a constant percentage of the rate of the previous year. All p values < 0.05 were considered statistically significant.

## Results

### Incidence and mortality in 2012

**Figure 1:**
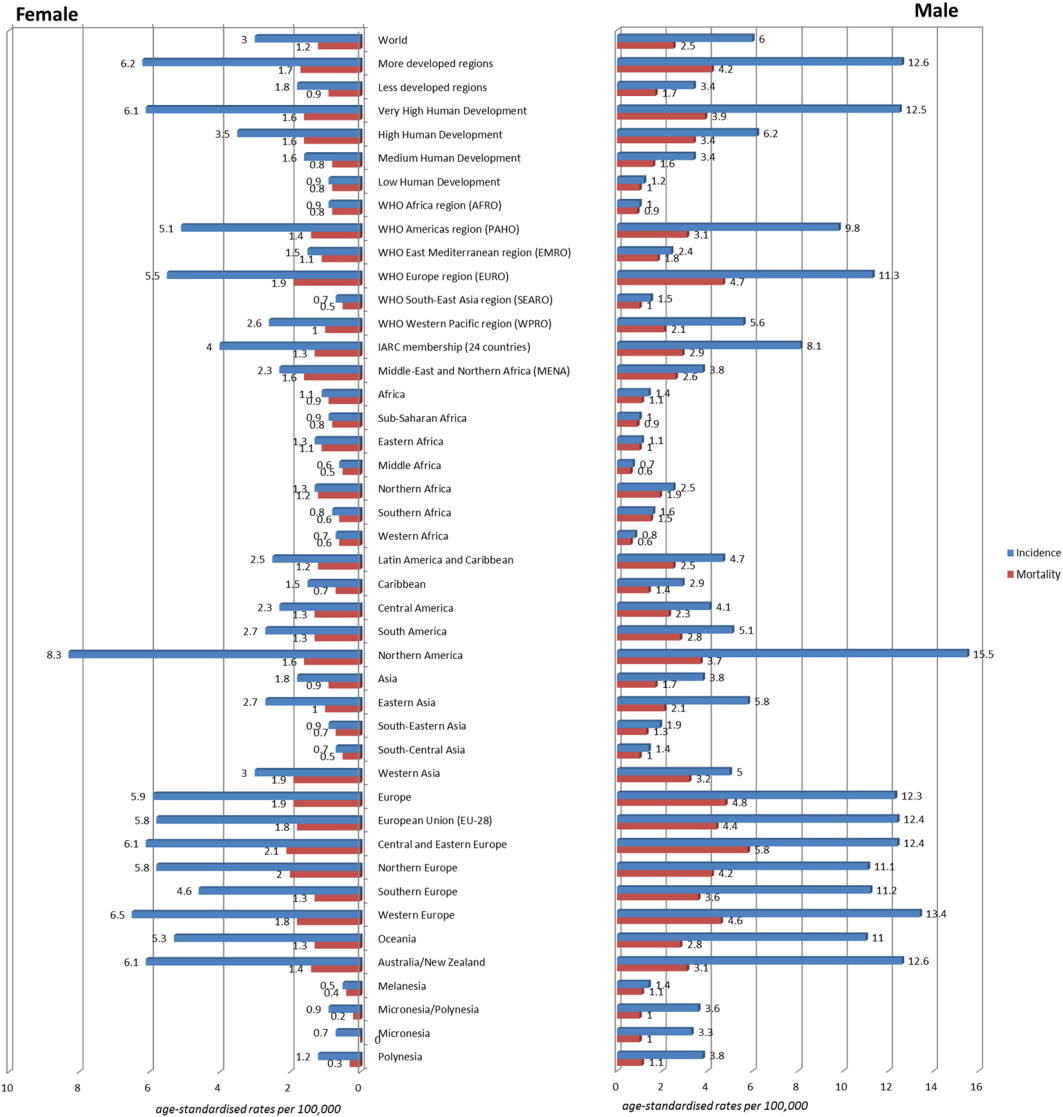
The age-incidence incidence (left) and mortality (right) rates of kidney cancer in 2012 by gender and world regions.

A total of 337,860 new cases of kidney cancer and 143,406 related deaths were reported in 2012. The highest incidence were found in Czech Republic (ASR 16.7 per 100,000), Lithuania (13.2), Slovakia (12.5) and Northern America (11.7), and the lowest were reported in Middle Africa (0.6), Western Africa (0.7) and Sub-Saharan Africa (0.9) (Fig. 1). The incidence was substantially higher in countries with very high HDI (9.1) than those with high (4.7), medium (2.5) and low HDI (1.0).

The mortality rates were higher in more developed than less developed regions (2.8 vs. 1.3). The highest mortality rates were reported in the Lithuania (4.9), Czech Republic (4.8), Latvia (4.7) and Estonia (4.6). The lowest estimated death rates were found in Micronesia/Polynesia (0.6), Middle Africa (0.6), Western Africa (0.6) and South-Central Asia (0.7). These geographical variations were similar when the worldwide incidence was stratified by gender (Table 2).

**Table 2:** The estimated incidence and mortality of kidney cancer according to world area (2012).

World regions	Population size Male, (1,000)	New cases		Mortality		Population size Female, (1,000)	New cases		Mortality	
		n	ASR	n	ASR		n	ASR	n	ASR
**Africa**	549,445	5133	1.4	4900	1.1	549 608	4155	1.1	4014	0.9
Eastern Africa	180,243	1401	1.1	1839	1.3	182 469	1191	1.0	1553	1.1
Middle Africa	69,179	380	0.7	341	0.6	69 644	337	0.6	308	0.5
Northern Africa	106,147	2014	2.5	1468	1.6	105 353	1497	1.9	1104	1.2
Southern Africa	29,735	325	1.6	220	0.8	30 816	283	1.5	170	0.6
Western Africa	164,141	1013	0.8	1032	0.7	161 327	847	0.6	879	0.6
**Asia**	2,179,003	81380	3.8	42022	1.8	2 081 150	36751	1.7	20307	0.9
Eastern Asia	813,296	61482	5.8	31152	2.7	777 374	23697	2.1	13063	1.0
South-Eastern Asia	305,225	4910	1.9	2811	0.9	306 008	3363	1.3	1933	0.7
South-Central Asia	933,786	10406	1.4	5121	0.7	881 514	6907	1.0	3504	0.5
Western Asia	126,697	4582	5.0	2938	3.0	116 253	2784	3.2	1807	1.9
**America**	303,514	6596	3.8	4406	2.3	310 360	4281	2.6	2911	1.6
Caribbean	20,951	650	2.9	380	1.5	21 313	316	1.4	188	0.7
Central America	82,227	2771	4.1	1721	2.3	83 632	1539	2.3	980	1.3
South America	200,336	9695	5.1	5966	2.7	205 415	5268	2.8	3017	1.3
North America	173,209	39781	15.5	24041	8.3	176 585	10662	3.7	5979	1.6
**Europe**	355,275	71790	12.3	43462	5.9	381 747	31338	4.8	17687	1.9
Central and Eastern Europe	138,249	23803	12.4	16550	6.1	155 701	11695	5.8	6852	2.1
Northern Europe	49,574	9539	11.1	5900	5.8	51 252	4100	4.2	2579	2.0
Southern Europe	74,900	15405	11.2	7622	4.6	78 393	5901	3.6	2979	1.3
Western Europe	92,553	23043	13.4	13390	6.5	96 400	9642	4.6	5277	1.8
**Oceania**	18,859	2724	11.0	1444	5.3	18 746	773	2.8	432	1.3
Australia/New Zealand	13,632	2666	12.6	1421	6.1	13 715	740	3.1	418	1.4
Melanesia	4,628	39	1.4	17	0.5	4 451	28	1.1	13	0.4
Micronesia/Polynesia	258	19	3.6	6	0.9	580	5	1.0	1	0.2
More developed regions	604,008	125378	12.6	74613	6.2	637 294	47917	4.2	27031	1.7
Less developed regions	2,975,297	88546	3.4	49323	1.8	2 880 901	42885	1.7	25573	0.9
**World**	3,579,305	213924	6.0	123936	3.0	3 518 195	90802	2.5	52604	1.2

ASR = Age standardized rate per 100,000. Source: GLOBOCAN 2012^1^. Numbers are rounded to the nearest 10 or 100, and may not add up to the total. The population size of the world regions were retrieved from the Population Reference Bureau, Washington, DC. This population size is, however, not necessarily identical as that available in GLOBOCAN. Available at: http://www.prb.org/Publications/Datasheets/2012/world-population-data-sheet/world-map.aspx#/table/population.

### Correlation between incidence/mortality and socioeconomic development

**Figure 2:**
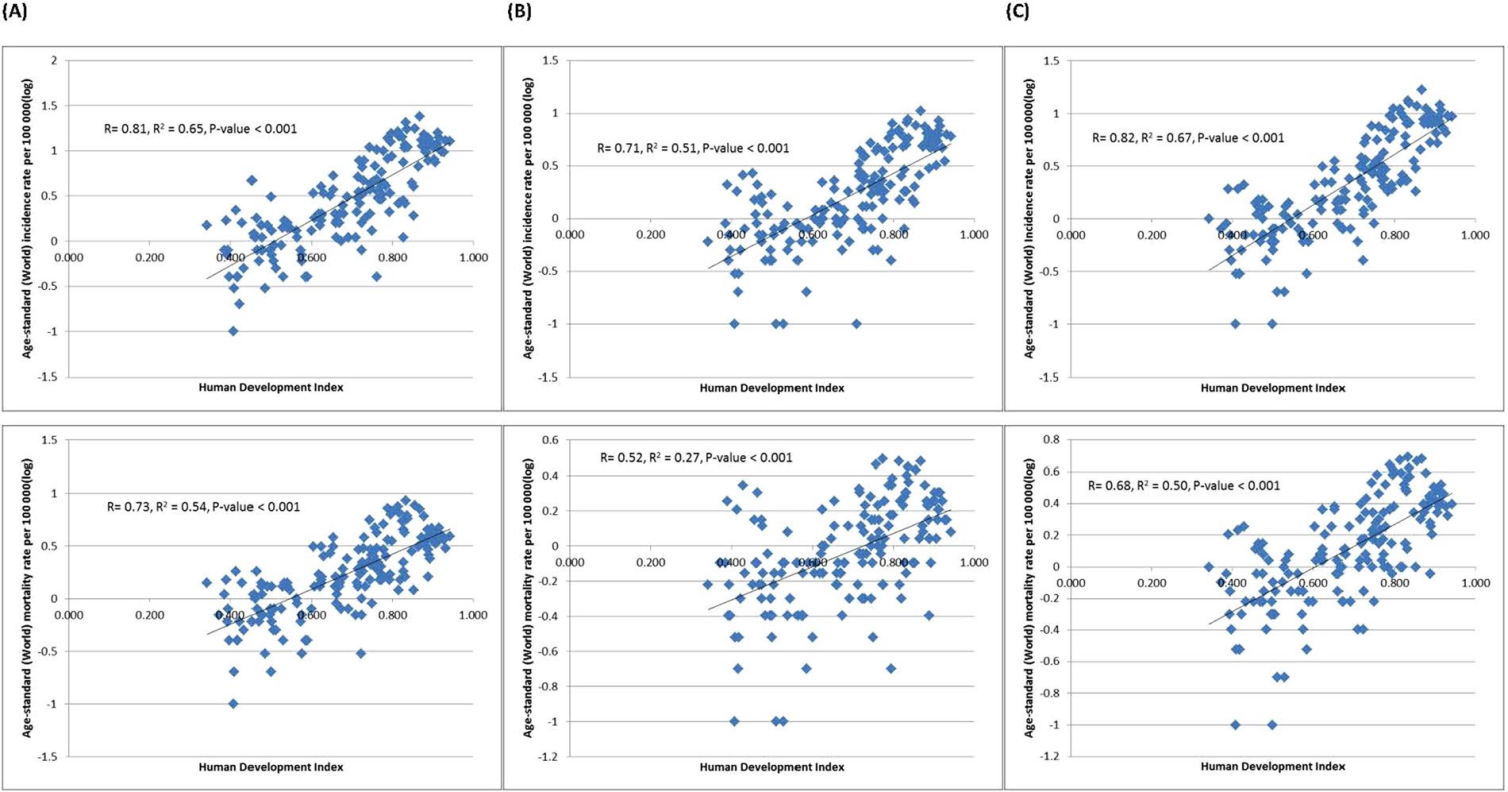
(**A**) Correlation between age-standardised kidney cancer incidence (upper panel) and mortality (lower panel) and Human Development Index (HDI) (Male). (**B**) Correlation between age-standardised kidney cancer incidence (upper panel) and mortality (lower panel) and Human Development Index (HDI) (Female). **(C)** Correlation between age-standardised kidney cancer incidence (upper panel) and mortality (lower panel) and Human Development Index (HDI) (Both sex).

**Figure 3:**
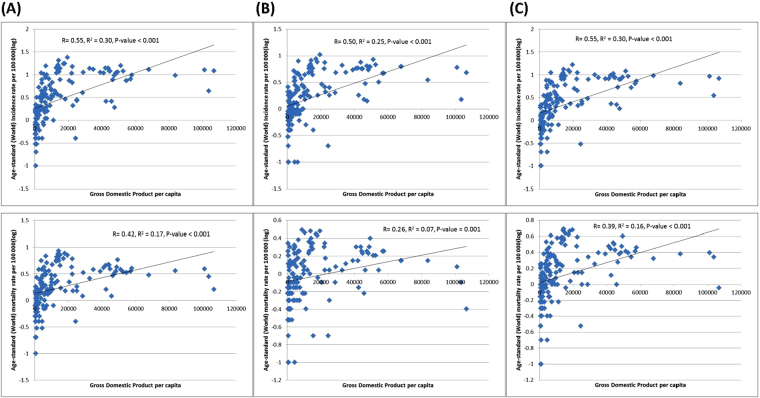
(**A**) Correlation between age-standardised kidney cancer incidence (upper panel) and mortality (lower panel) and Gross Domestic Product (GDP) (Male). (**B**) Correlation between age-standardised kidney cancer incidence (upper panel) and mortality (lower panel) and Gross Domestic Product (GDP) (Female). (**C**) Correlation between age-standardised kidney cancer incidence (upper panel) and mortality (lower panel) and Gross Domestic Product (GDP) (Both sex).

Figure 2A,B and C showed the correlation between the incidence/mortality and HDI, evaluated by simple linear regression analysis. The ASR of incidence (r = 0.82, r^2^ = 0.67) and mortality (r = 0.68, r^2^ = 0.50) increased with higher levels of HDI. Similarly, the ASR of incidence (r = 0.55, r^2^ = 0.30) and mortality (r = 0.39, r^2^ = 0.16) was also correlated with GDP per capita (all p < 0.001; Fig. 3A,B and C). These significant correlations between the ASR figures and HDI/GDP were similar when analyzed separately for men and women.

### Temporal trends

The incidence and mortality trend of each country was shown in Supplementary Figure 1, and the corresponding findings from the joinpoint regression analysis were presented in Supplementary Figures 2 and 3. Many countries experienced incidence rise, in particular Brazil, Ecuador, Thailand and Bulgaria. A substantial reduction in mortality rates was observed for many countries such as Sweden and Denmark, yet increases in mortality rates were observed in some Eastern European countries like Bulgaria.

#### Latin America and the Caribbean

**Figure 4:**
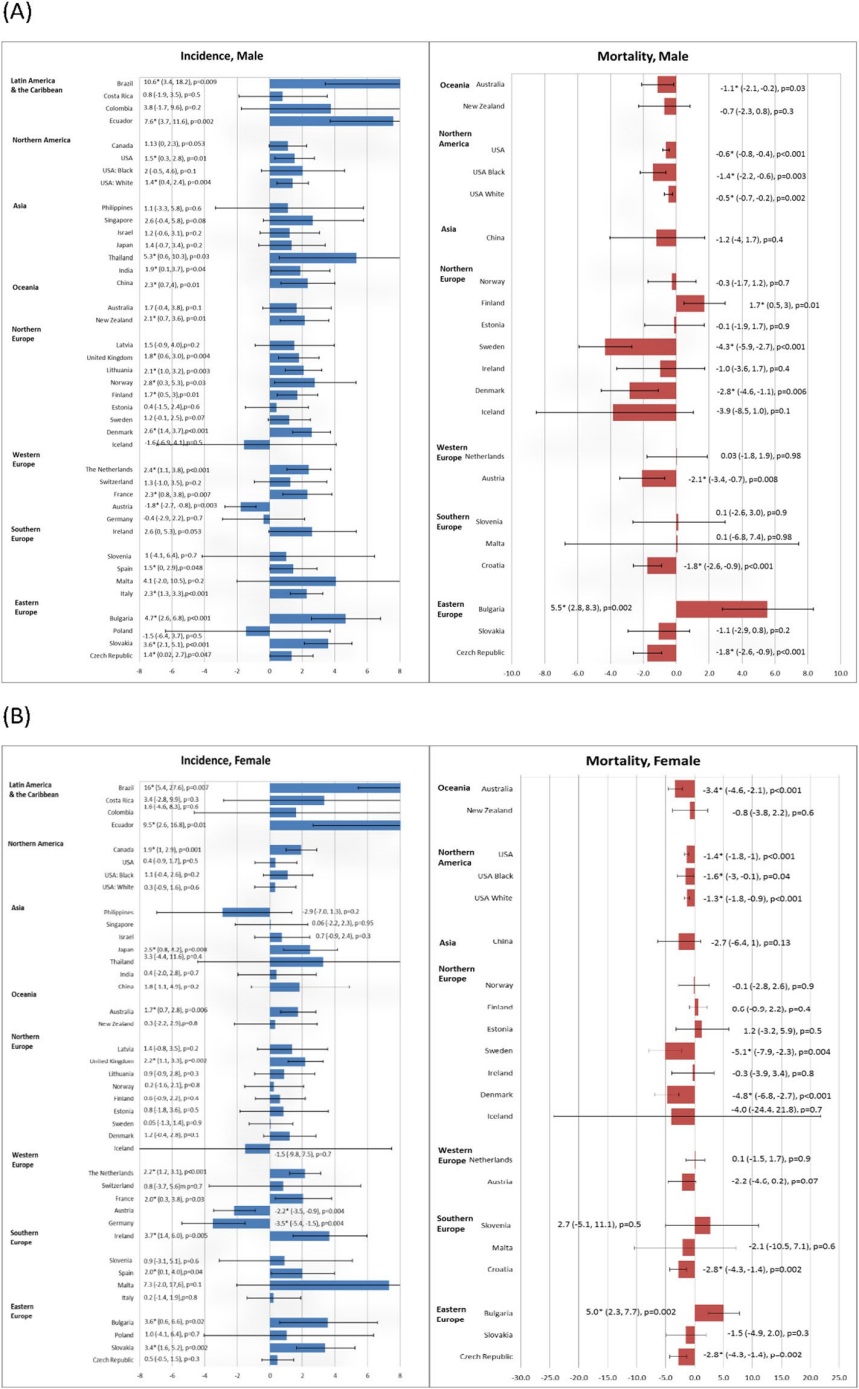
(**A**) The Average Annual Percent Change (AAPC) in the incidence of kidney cancer in male (left) and female (right) in the most recent 10 years. (**B**) The Average Annual Percent Change (AAPC) in the mortality of kidney cancer in male (left) and female (right) in the most recent 10 years.

Brazil (AAPC = 10.6, 95% C.I. = 3.4, 18.2, p = 0.009) and Ecuador (AAPC = 7.6, 95% C.I. = 3.7, 11.6, p = 0.002) showed substantial increase in incidence among men, and the rise in incidence among women was even more marked in these two countries (Brazil: AAPC = 16, 95% C.I. = 5.4, 27.6, p = 0.007; Ecuador: AAPC = 9.5, 95% C.I. = 2.6, 16.8, p = 0.01) (Fig. 4A).

#### Northern America

Its incidence showed a slight rise in male Americans (overall AAPC = 1.5, 95% C.I. = 0.3, 2.8, p = 0.01). Among women, Canada (AAPC = 1.9, 95% C.I. = 1, 2.9, p = 0.001) also showed a rise in incidence. There was a slight decline in mortality rates in Americans (Fig. 4B).

#### Asia

Thailand (AAPC = 5.3, 95% C.I. = 0.6, 10.3, p = 0.03), China (AAPC = 2.3, 95% C.I. 0.7, 4, p = 0.01), and India (AAPC = 1.9, 95% C.I. 0.1, 3.7, p = 0.04) were countries showing an increase in incidence among men, whilst Japan (AAPC = 2.5, 95% C.I. = 0.8, 4.2, p = 0.008) reported an incidence increase among women.

#### Oceania

The incidence in New Zealand was estimated to increase among men (AAPC = 2.1, 95% C.I. = 0.7, 3.6, p = 0.01), whilst Australia reported an increase among women (AAPC = 1.7, 95% C.I. = 0.7, 2.8, p = 0.006). The mortality decreased in Australia among both men and women (Fig. 4B).

#### Northern Europe

There was an incidence increase among men in the United Kingdom, Lithuania, Finland, Norway and Denmark (AAPC ranging from 1.8 to 2.8), as well as women in United Kingdom (AAPC = 2.2, 95% C.I. = 1.1, 3.3, p = 0.002). However, Sweden and Denmark showed a mortality decline in both men (−4.3, 95% C.I. −5.9, −2.7, p < 0.001 and −2.8, 95% C.I. −4.6, −1.1, p = 0.006) and women (−5.1, 95% C.I. −7.9, −2.3, p = 0.004 and −4.8, 95% C.I. −6.8, −2.7, p < 0.001).

#### Western Europe

The Netherlands (AAPC = 2.4, 95% C.I. = 1.1, 3.8, p < 0.001 [men]; AAPC = 2.2, 95% C.I. = 1.2, 3.1 p = 0.001 [women]), France (AAPC = 2.3, 95% C.I. = 0.8, 3.8, p = 0.007 [men]; AAPC = 2, 95% C.I. = 0.3, 3.8 p = 0.03 [women]) and Ireland (AAPC = 3.7, 95% C.I. = 1.4, 6.0, p = 0.005 [women]) reported an incidence increase. Austria (AAPC = −1.8, 95% C.I. = −2.7, −0.8, p = 0.003 [men]; AAPC = −2.2, 95% C.I. = −3.5, −0.9, p = 0.004 [women]) and Germany (AAPC = −3.5, 95% C.I. = −5.4, −1.5, p = 0.004 [women]) showed an incidence decline. Austria also showed a mortality reduction in men (AAPC = −2.1, 95% C.I. = −3.4, −0.7, p = 0.008).

#### Southern Europe

Spain had a slight increase in incidence among men (AAPC = 1.5, 95% C.I. = 0, 2.9, p = 0.048) and women (AAPC = 2.0, 95% C.I. = 0.1, 4, p = 0.04). Italy also reported a rise in incidence among men (AAPC = 2.3, 95% C.I. = 1.3, 3.3, p < 0.001). For mortality, Croatia was the only country showing a decline.

#### Eastern Europe

Bulgaria (AAPC = 4.7, 95% C.I. = 2.6, 6.8, p < 0.001 [men]; AAPC = 3.6, 95% C.I. = 0.6, 6.6, p = 0.02 [women]), Slovakia (AAPC = 3.6 95% C.I. = 2.1, 5.1, p < 0.001 [men]; AAPC = 3.4, 95% C.I. = 1.6, 5.2, p = 0.002 [women]), and Czech Republic (AAPC = 1.4, 95% C.I. = 0.02, 2.7, p = 0.047 [men]) reported a rise in incidence. Bulgaria also showed a significant increase in mortality (AAPC = 5.5, 95% C.I. = 2.8, 8.3, p = 0.002 [men]; AAPC = 5, 95% C.I. = 2.3, 7.7, p = 0.002 [women]).

### AAPC-based projections of incidence and mortality to 2020 and 2030

By 2030, countries with the greatest rise in incidence included Brazil (911%), Ecuador (437%), Thailand (230%), Bulgaria (186%), and Malta (131%) in men. The most drastic rise in incidence by 2030 was found in Brazil (2,938%) and Ecuador (709%) in women. Bulgaria had the biggest rise in mortality in both men (227%) and women (192%) by 2030 (Supplementary Figures 4 and 5).

## Discussion

This study presented the most updated global epidemiological profiles of kidney cancer, and we described the incidence and mortality patterns and trends based on high quality data. Both the incidence and mortality rates were positively correlated with human development levels and GDP per capita. The coefficients of correlation between incidence/mortality and HDI (0.62–0.73), and to a lesser extent GPD (0.36–0.55), were high. Incidence figures in the most recent 10 years reported that a total of 18 and 12 countries/regions, respectively, experienced increases in incidence rates in men and women. Brazil, Ecuador, Thailand and Bulgaria were some countries where incidence trends increased sharply. Many countries reported reduction in mortality trends, in particular Northern Europe (Sweden, Denmark) and Western Europe (Austria). Bulgaria was the only country that showed a substantially increasing mortality trend. We have also projected the incidence and mortality figures of kidney cancer for some selected countries.

Several reasons could explain the higher incidence of kidney cancer in more developed countries, and their positive correlation with HDI and GDP. Firstly, in developed nations with more rapid development and higher productivity, the prevalence of risk factors for RCC including smoking, obesity, physical inactivity and hypertension was higher^7^. Another explanation for the higher incidence could be attributed to the more liberal use of imaging techniques in more resource-privileged countries, such as abdominal ultrasound and computed tomography for non-specific symptoms presented by patients^3^. Indeed, most of the increases in incidence have been attributed to diagnosis of early, local stage tumors^7^. Yet another possible but more speculative contributing factor includes certain occupational and environmental exposures to carcinogenic agents, such as trichloroethylene, cadmium, arsenic, radon and nitrate^3,7^. As only 2–3% of all RCC were familial with distinct genetic phenotypes, hereditary factors seem to be rather remote in its influence on incidence rates. Our findings that some countries outside Europe and North America reported markedly increased incidence trends warrant further studies to elucidate the underlying etiological mechanisms.

The use of linear modelling for trend projection allows comparison between our findings and those reported from other literature. For instance, Bailey and colleagues have examined the incidence rates of colorectal cancer in the United States by using the Surveillance, Epidemiology and End Results (SEER) database, and the incidence was assumed to change at a constant proportion of the annual percent change (APC) of the previous year^30^. Other studies that projected future trends of cancer incidence and mortality also adopted linear modelling^31,32^. In addition, there has been a study that compared the validity of using 15 different models to predict cancer incidence, including linear and non-linear models and one on a smoothed version of the age-period-cohort model. It was found that none of the models significantly out-performed one another^33^. Nevertheless, despite the common use of linear regression for prediction of cancer statistics, there are some caveats that should be mentioned. Projected figures could be underestimated due to emergence of known and unknown risk factors, or on the other hand overestimated with more developed preventive efforts that could lead to a decrease in cancer incidence and mortality^34^. While risk factors related to lifestyle measures are not necessarily correlated with higher cancer incidence or mortality due to the relatively short latency period, an aging population and the growing proportion of elderly with longer life expectancy are associated with an escalated cancer burden in the future^34^.

With respect to reporting bias, cancer registration in relatively less-developed nations could suffer from higher chance of under-reporting. Incidence and mortality figures in more regional cancer registries might be less accurate due to limited communication infrastructure and less robust reporting systems to the healthcare management for new cancer cases and deaths. Other contributing factors to reporting bias include relative lack of clinical services in poor rural areas; low income levels of the general populations and thus lower willingness to utilize healthcare services; limited access to healthcare providers in underdeveloped regions; as well as attribution of cancer diagnoses and death to other diseases due to lower availability of confirmatory investigation tests required for cancer diagnosis. These factors could significantly underestimate the actual incidence and mortality figures when trends were estimated and future incidence/mortality rates were projected, in particular for less developed nations. These should alert cautious interpretation of this analysis that compared the incidence/mortality of kidney cancer among countries.

To address reporting bias as a major source of inaccuracy in analysis of incidence and mortality trends, resources are required to build national cancer registries that capture data in a systematic manner with comprehensive coverage of healthcare service providers. This should be in parallel with regular audit of the hospital case records on disease coding and clinical guidelines to enhance cancer incidence/mortality reporting to national databases. There have been studies conducted in the US that installed electronic health records and developed implementation strategy of new cancer-reporting module in rural clinics to increase cancer reporting rates and accuracy of reporting mortality causes^35^. Strategies to enhance service accessibility for patients with symptoms of cancer to facilitate timely diagnosis are also required. In addition, studies that adopt population-based designs, such as representative surveys that collect data directly from individuals, could supplement data for more precise estimation of cancer incidence and mortality.

The mortality patterns and their correlation with HDI/GDP were similar to that of the cancer’s incidence. Although more developed countries are equipped with technological advancement in disease treatment, it is well recognized that a significant proportion (up to 30%) of patients have metastatic disease during initial diagnosis, whilst another 20% of patients who receive nephrectomy will relapse and develop metastasis during follow-up^3^ - hence the close concordance between incidence and mortality. Nevertheless, the reduction in mortality trends in the recent decade could be explained by the earlier diagnosis leading to stage migration to earlier stage disease, which could be treated by curative intervention. Another driver for the mortality decline could be due to better systemic therapy for advanced diseases, such as interferon and interleukin-2 therapy^36^, and targeted therapy^37^.

Other limitations of this study should be discussed. Firstly, only one-third and one-fifth of the world’s countries, respectively, reported incidence and mortality data of high quality - data precision, coverage, and completeness in the national databases could be different from that of CI5. Furthermore, one could not establish cause-and-effect relationships in correlational analysis, and coincidental changes in lifestyle habits could parallel that of incidence/mortality rates. For instance, increased mortality rates due to kidney cancer is reminiscent of what is happening in Eastern Europe, particularly Bulgaria for coronary heart disease^38^, possibly related to limited preventive measures or poor dietary habits due to economic burden and increase in tobacco smoking and alcohol drinking that are heavily associated with physical inactivity, obesity and hypertension. Also, the analyses have not taken competition of risk into account^39^. Furthermore, we have used a cutoff value of significance at p < 0.05, similar to previous studies that examined the temporal patterns and trends of cancer incidence and mortality^16–19,30^. One should be cautious when our findings are interpreted as there are multiple testing in this study. Lastly, despite our most inclusive approach to analyze the most recent data, the figures used are from 2012 at the latest and the temporal trends will need continuous updates.

## Conclusion

The incidence rates of kidney cancer increased in many countries analyzed in this study, and the mortality rates declined in a large number of nations, particularly in more developed regions. With population ageing and population growth, the absolute incidence of kidney cancer will show a continuing increasing trend out of proportion of the incidence increase here implies. Appropriate healthcare resources should be planned to cope with the increasing need for patient treatment, especially in more resource-deprived countries. Future studies are needed to explore the underlying mechanisms for these epidemiological trends with potential risk factors incorporated in further analysis.

## Electronic supplementary material

Supplementary Figures
