# Supplementary material for: Incidence and mortality of kidney cancer: temporal patterns and global trends in 39 countries
Source: Sci Rep. 2017 Nov 16;7:15698. doi: 10.1038/s41598-017-15922-4 (PMC5691143; doi:10.1038/s41598-017-15922-4)

Incidence and mortality of kidney cancer: temporal patterns and global trends in 38 countries

Martin CS Wong MD, MPH, Bill W Goggins DSc (Harvard), Benjamin HK Yip PhD, Franklin DH Fung BSc, Colette Leung BSc, Yuan Fang PhD, Samuel YS Wong MD, MPH (Johns Hopkins), CF Ng MD

Supplementary Figure 1 Temporal trends in the age-standardized incidence and mortality of kidney cancer according to country and gender

1). Latin America and the Caribbean

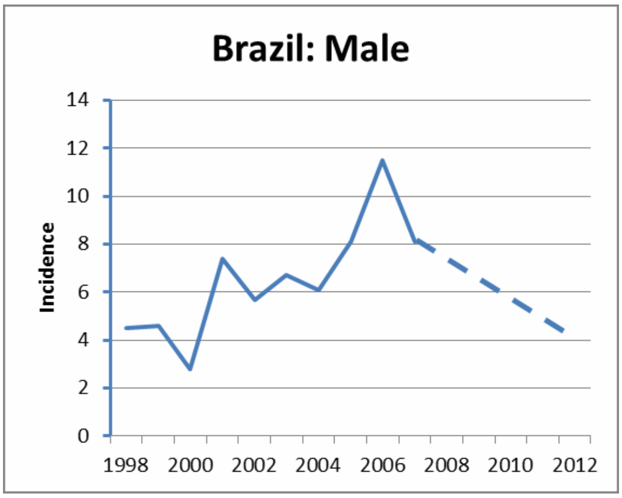

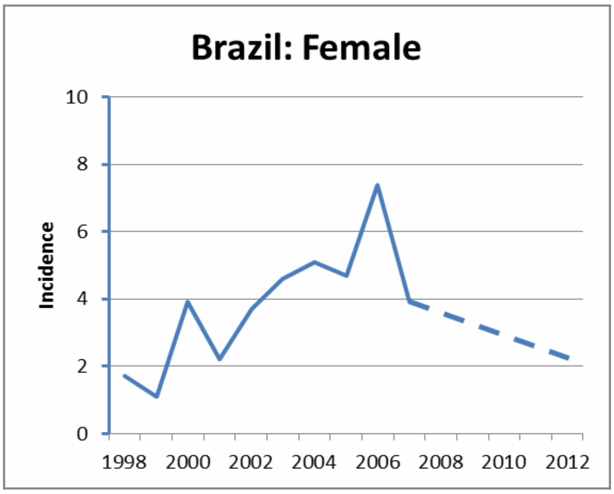

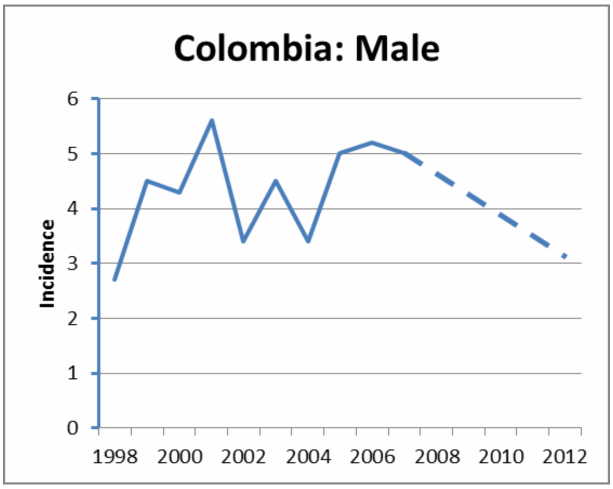

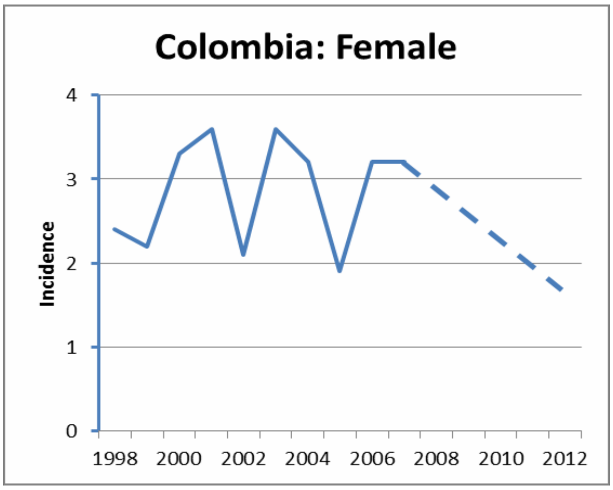

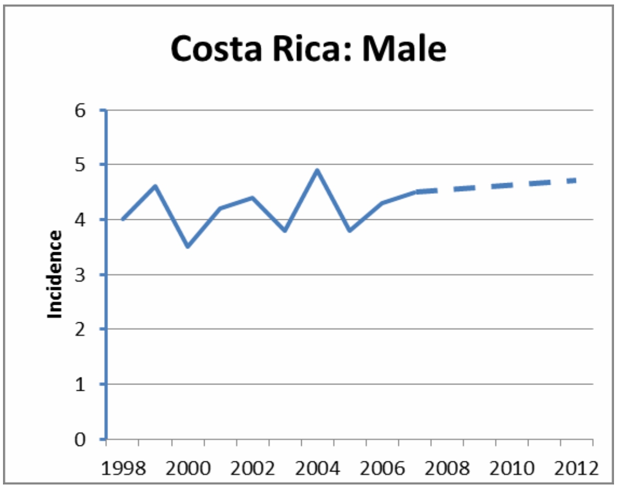

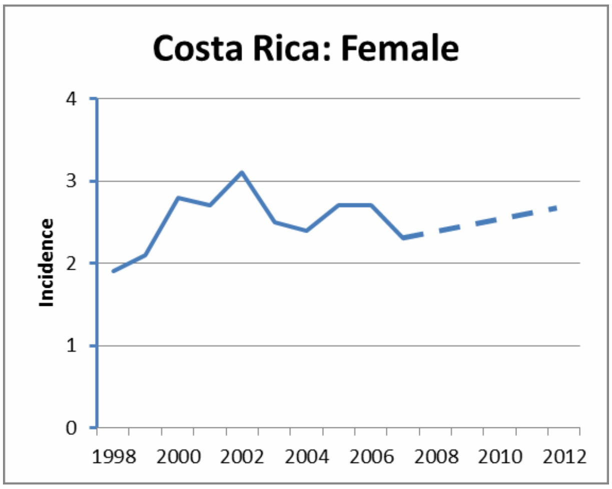

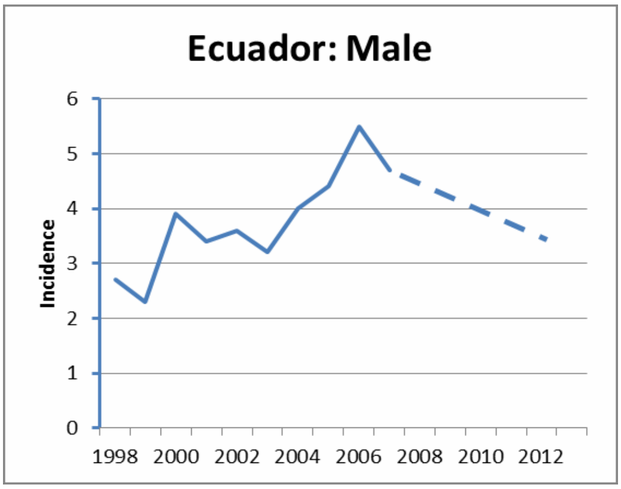

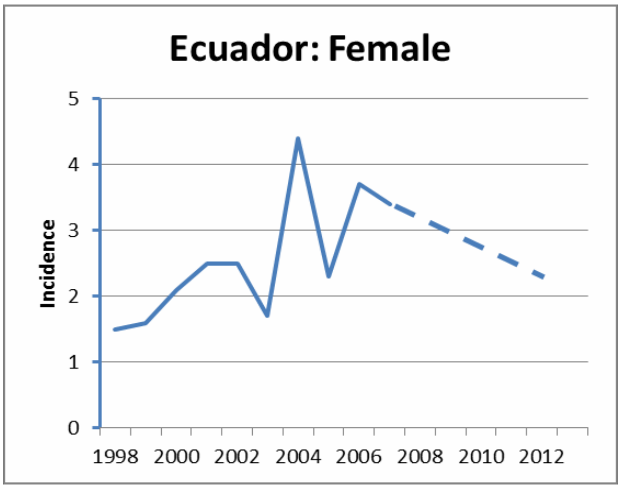

2). Northern America

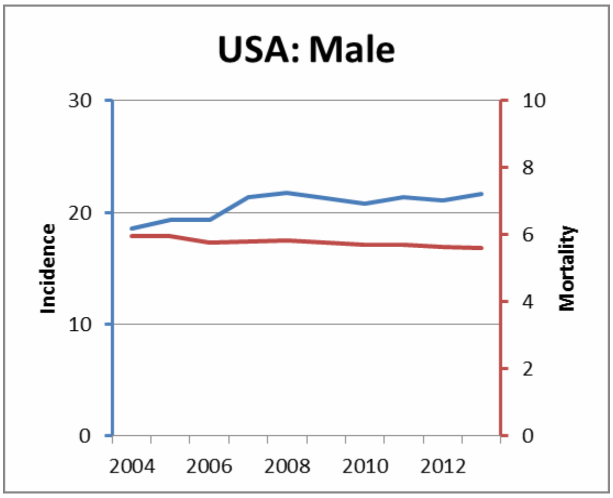

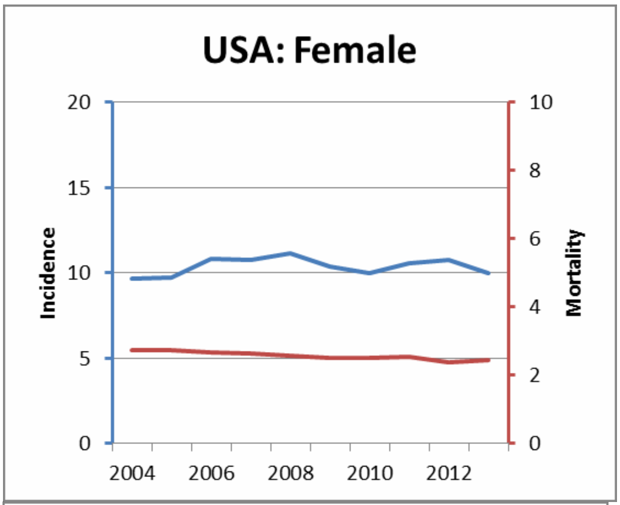

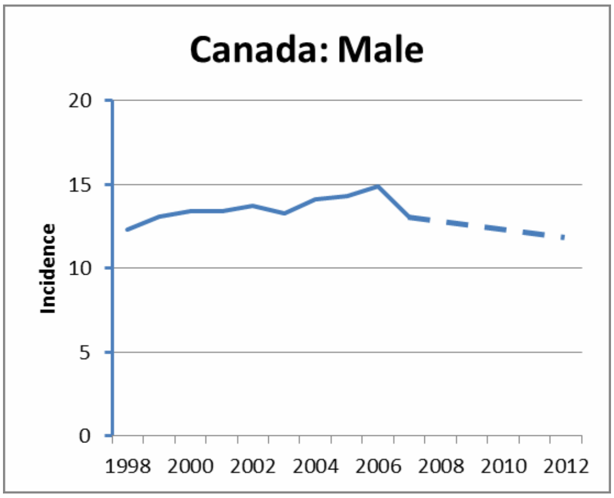

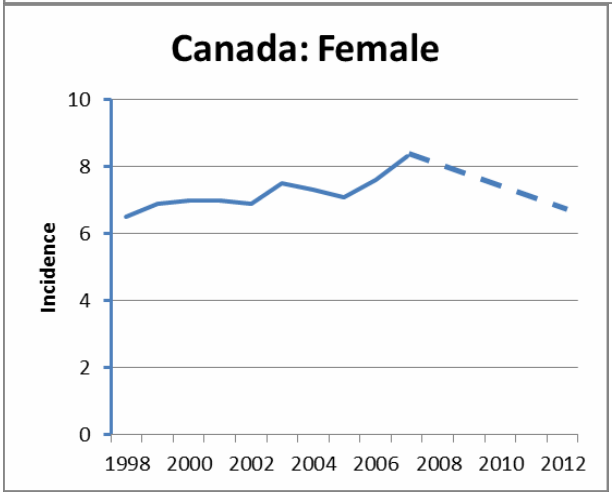

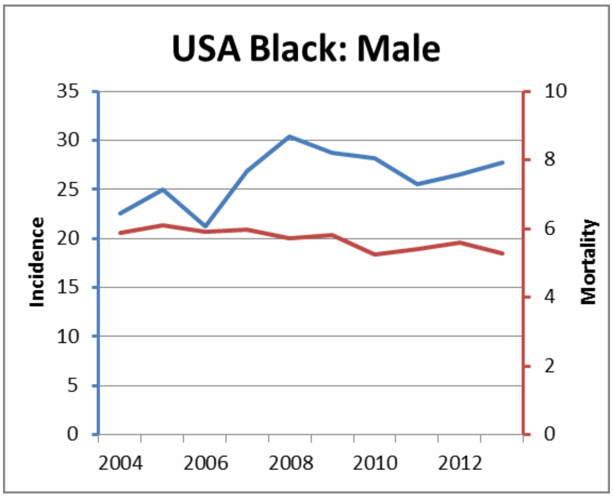
USA Black: Male
Incidence
Mortality
35
30
25
20
15
10
5
0
10
8
6
4
2
0
2004
2006
2008
2010
2012

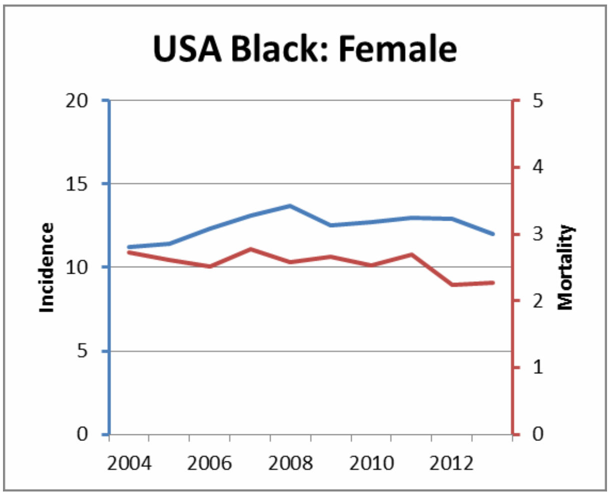
USA Black: Female
Incidence
Mortality
20
15
10
5
0
5
4
3
2
1
0
2004
2006
2008
2010
2012

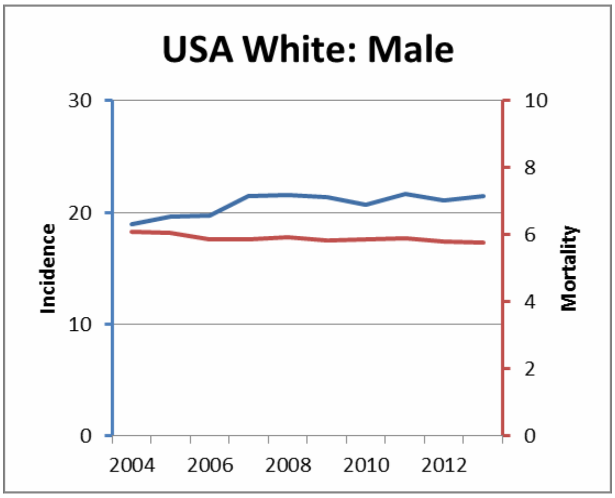
USA White: Male
Incidence
Mortality
30
20
10
0
10
8
6
4
2
0
2004
2006
2008
2010
2012

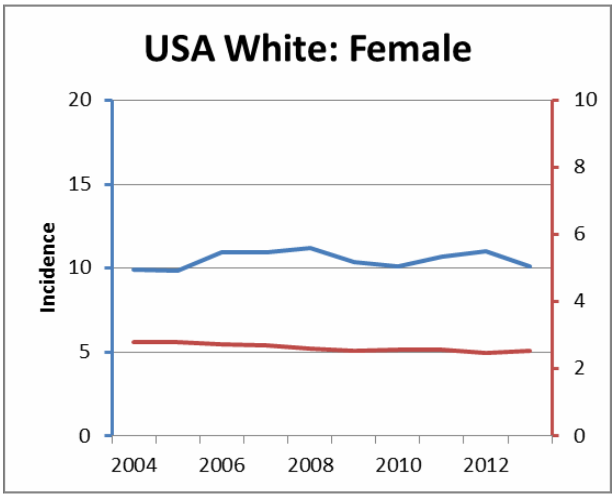
USA White: Female
Incidence
20
15
10
5
0
10
8
6
4
2
0
2004
2006
2008
2010
2012

3). Asia

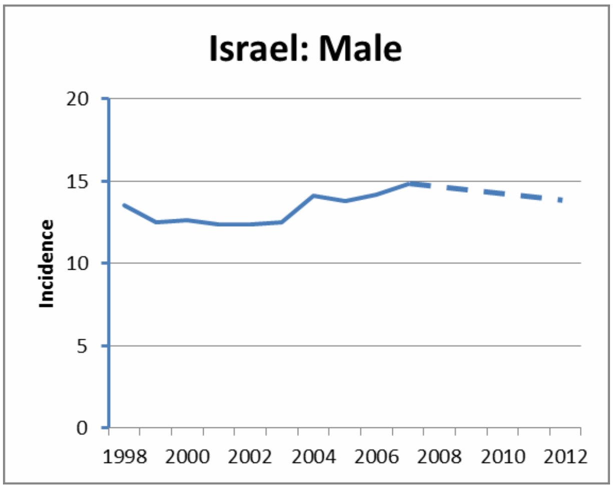
Israel: Male
Incidence
20
15
10
5
0
1998 2000 2002 2004 2006 2008 2010 2012

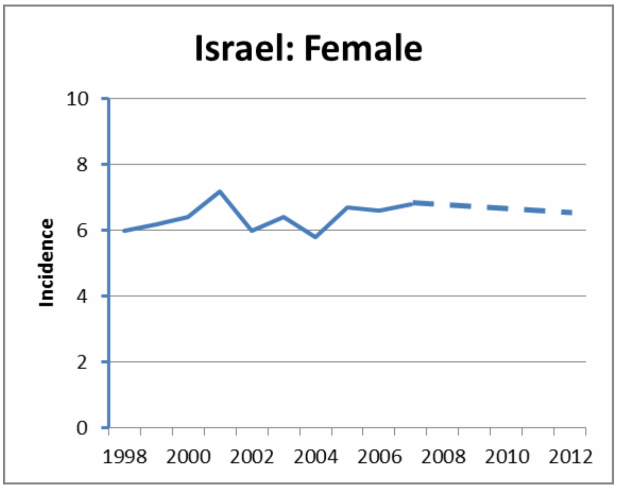
Israel: Female
Incidence
10
8
6
4
2
0
1998 2000 2002 2004 2006 2008 2010 2012

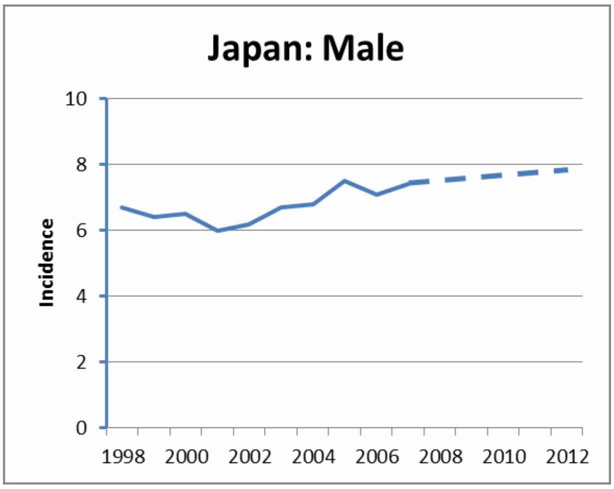
Japan: Male
Incidence
10
8
6
4
2
0
1998 2000 2002 2004 2006 2008 2010 2012

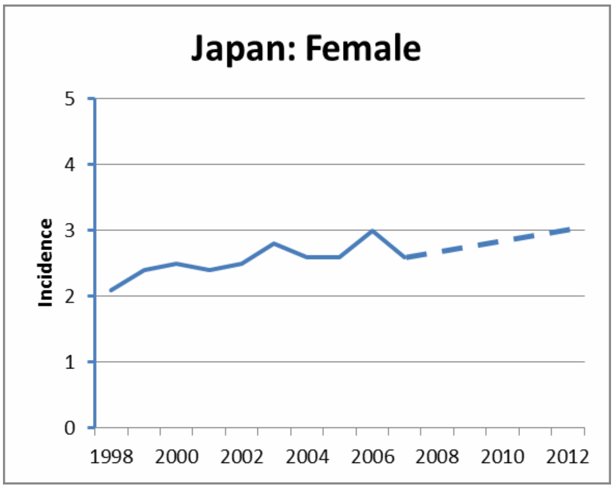
Japan: Female
Incidence
5
4
3
2
1
0
1998 2000 2002 2004 2006 2008 2010 2012

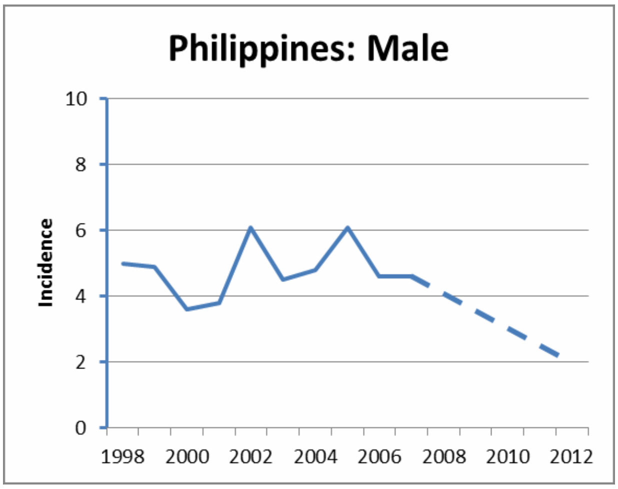
Philippines: Male
Incidence
10
8
6
4
2
0
1998 2000 2002 2004 2006 2008 2010 2012

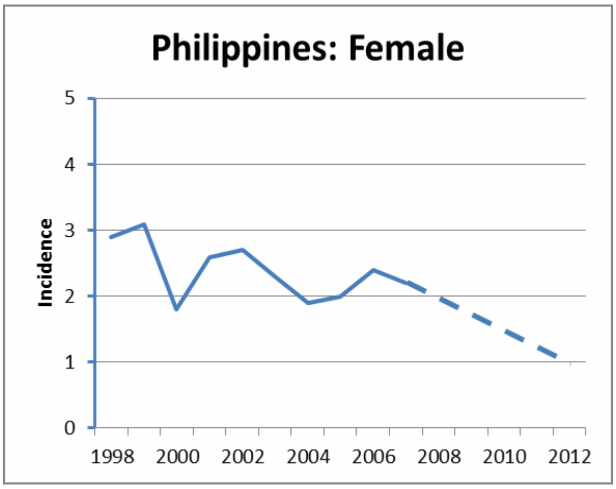
Philippines: Female
Incidence
5
4
3
2
1
0
1998 2000 2002 2004 2006 2008 2010 2012

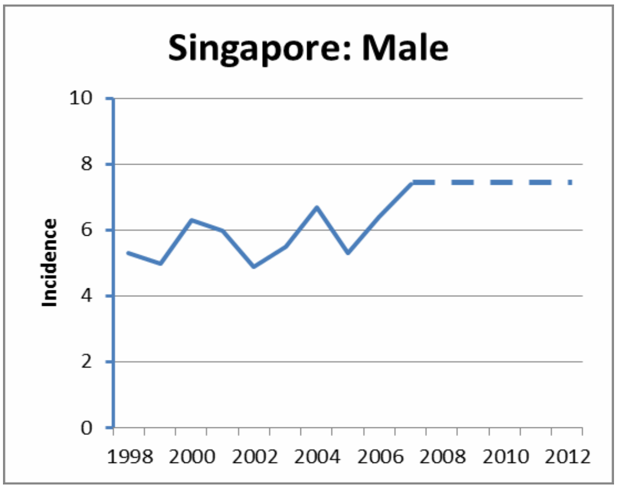
Singapore: Male
Incidence
0
2
4
6
8
10
1998
2000
2002
2004
2006
2008
2010
2012

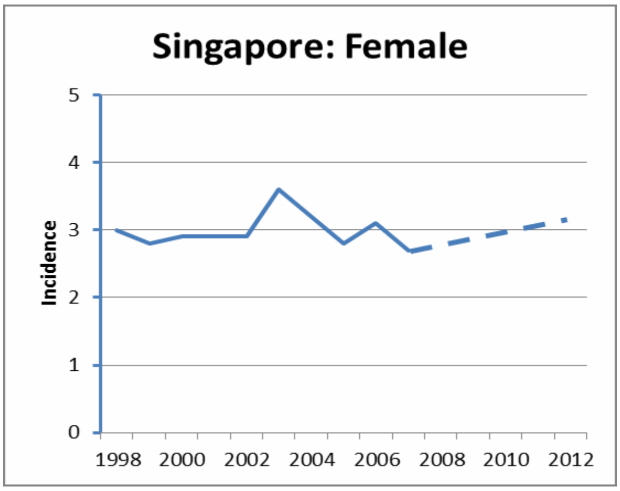
Singapore: Female
Incidence
0
1
2
3
4
5
1998
2000
2002
2004
2006
2008
2010
2012

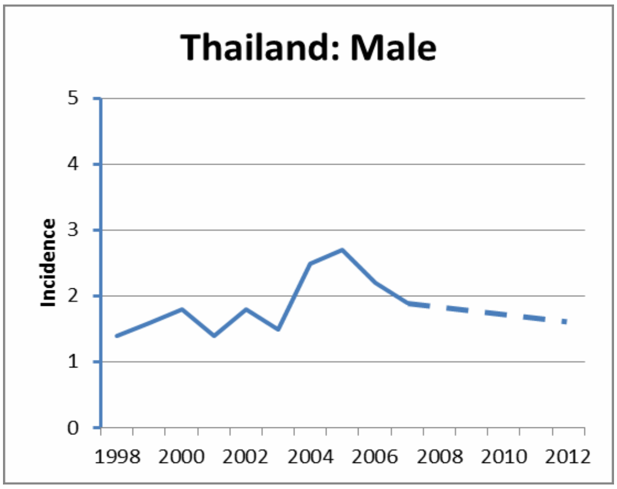
Thailand: Male
Incidence
0
1
2
3
4
5
1998
2000
2002
2004
2006
2008
2010
2012

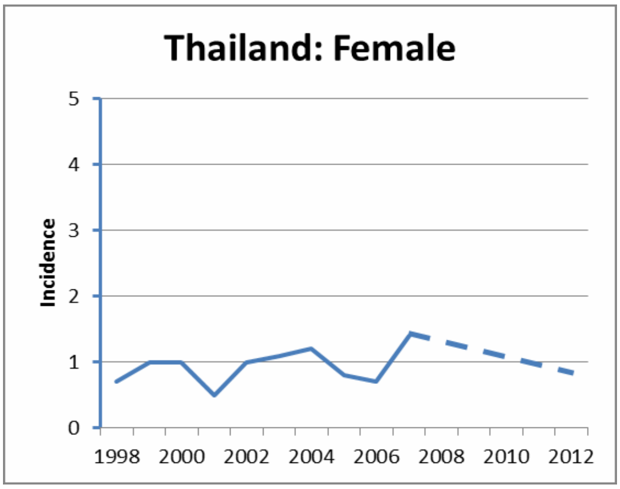
Thailand: Female
Incidence
0
1
2
3
4
5
1998
2000
2002
2004
2006
2008
2010
2012

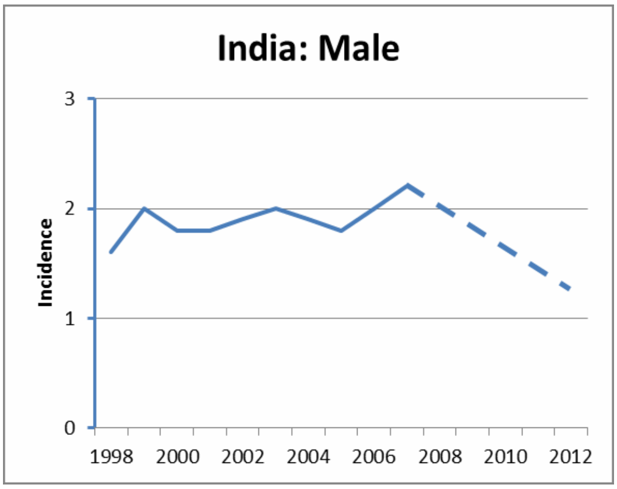
India: Male
Incidence
0
1
2
3
1998
2000
2002
2004
2006
2008
2010
2012

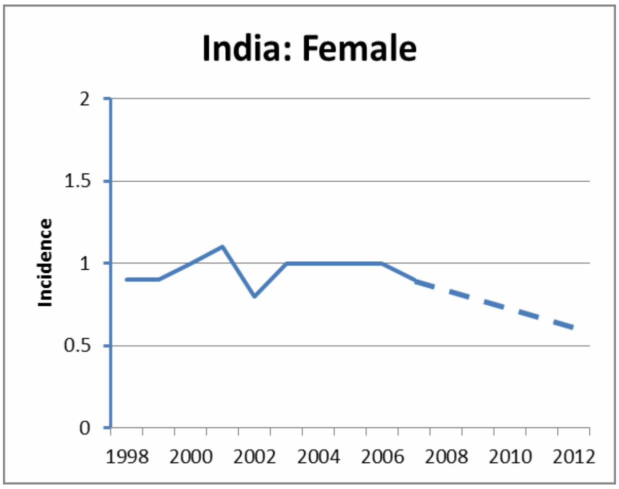
India: Female
Incidence
0
0.5
1
1.5
2
1998
2000
2002
2004
2006
2008
2010
2012

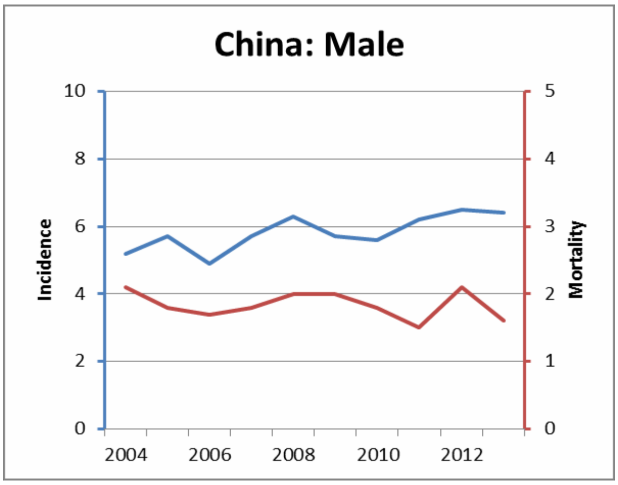
China: Male
Incidence
Mortality
10
8
6
4
2
0
5
4
3
2
1
0
2004
2006
2008
2010
2012

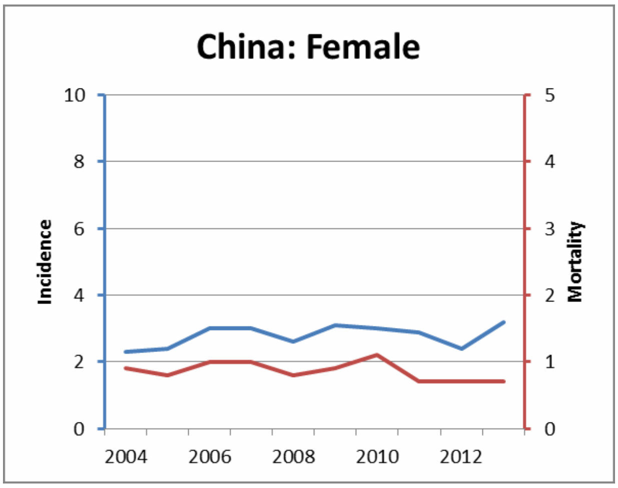
China: Female
Incidence
Mortality
10
8
6
4
2
0
5
4
3
2
1
0
2004
2006
2008
2010
2012

4). Oceania

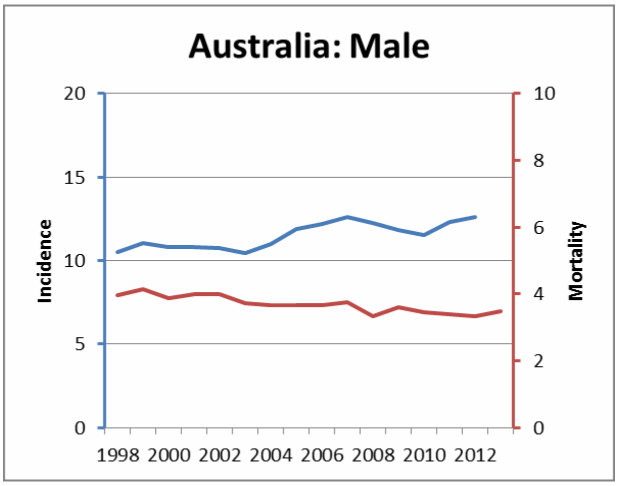
Australia: Male
Incidence
Mortality
20
15
10
5
0
10
8
6
4
2
0
1998 2000 2002 2004 2006 2008 2010 2012

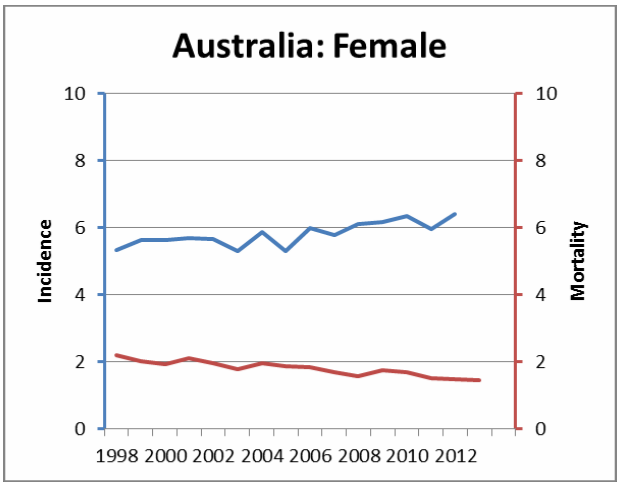
Australia: Female
Incidence
Mortality
10
8
6
4
2
0
10
8
6
4
2
0
1998 2000 2002 2004 2006 2008 2010 2012

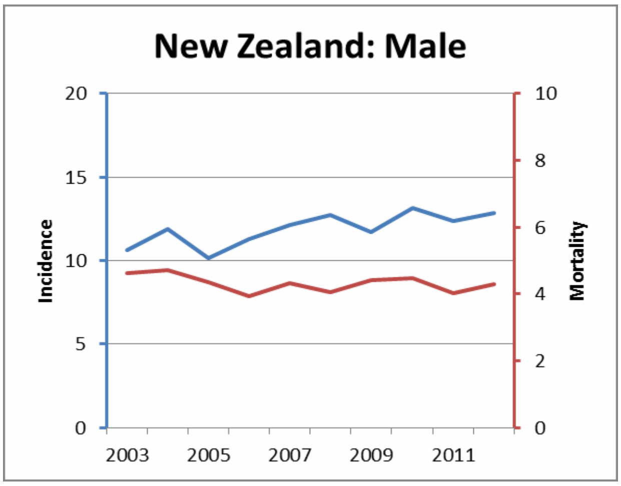
New Zealand: Male
Incidence
Mortality
20
15
10
5
0
10
8
6
4
2
0
2003
2005
2007
2009
2011

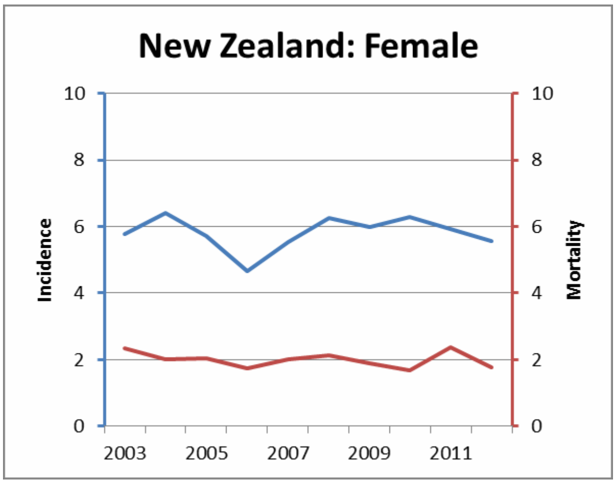
New Zealand: Female
Incidence
Mortality
10
8
6
4
2
0
10
8
6
4
2
0
2003
2005
2007
2009
2011

5). Northern Europe

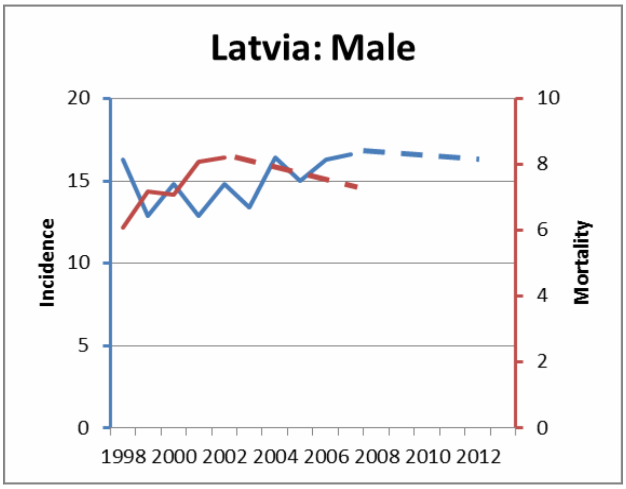

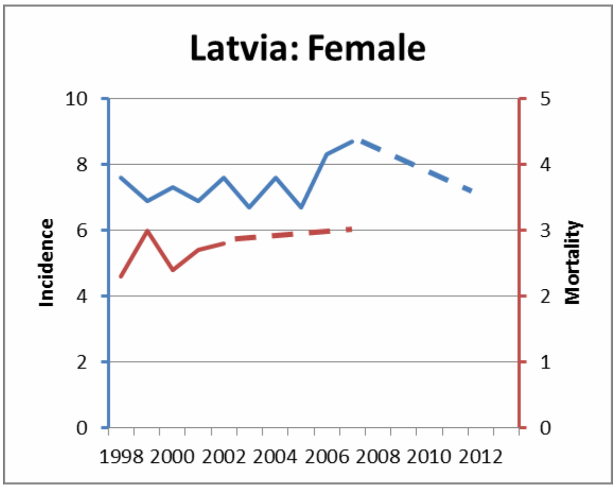

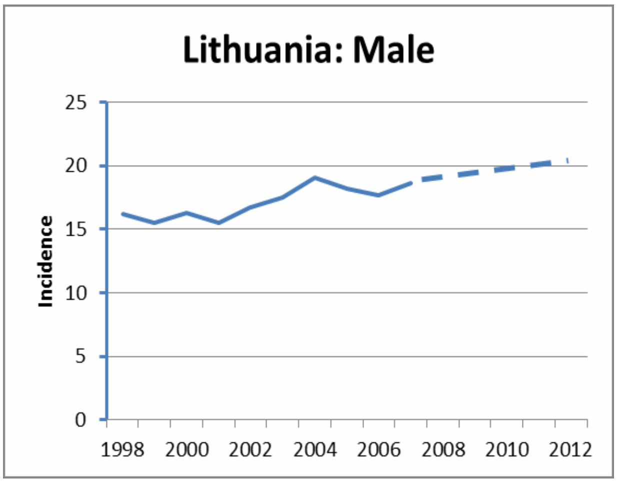

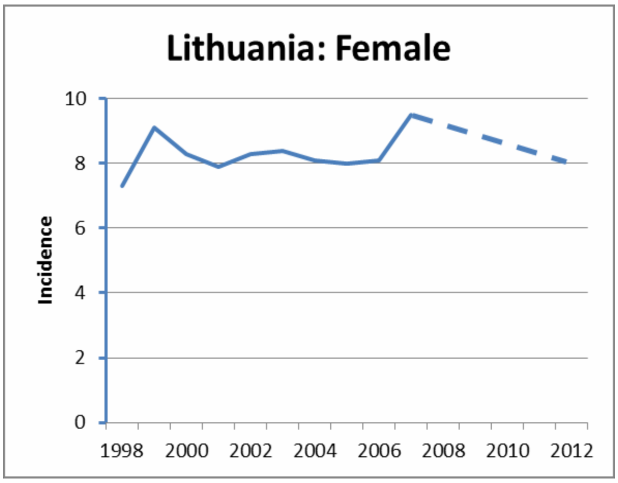

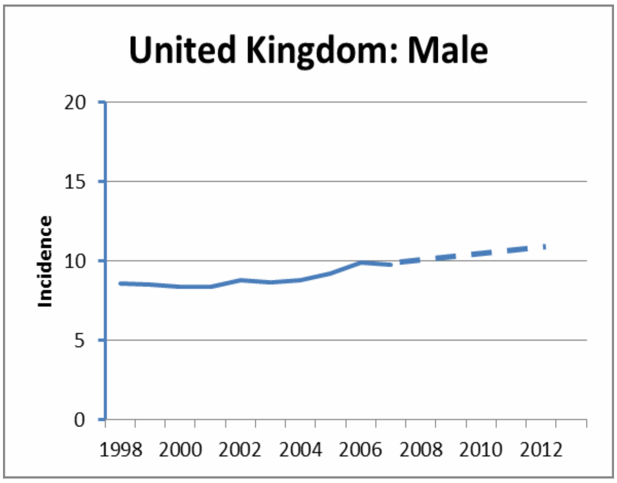

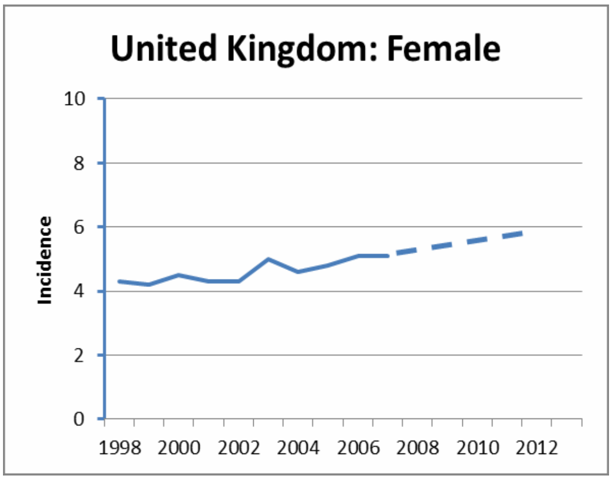

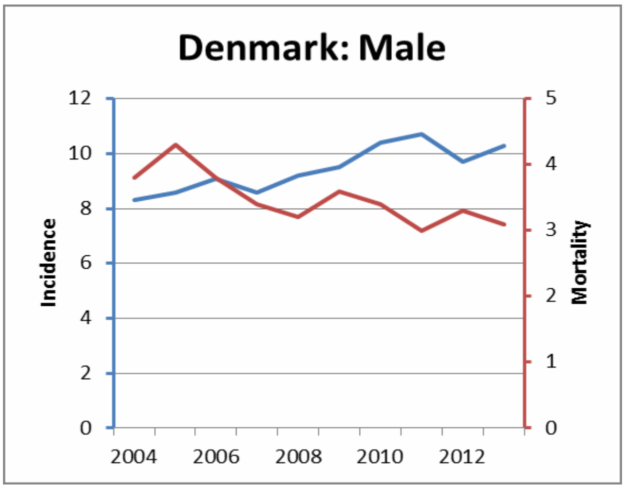
Denmark: Male
Incidence
Mortality
0 2 4 6 8 10 12
0 1 2 3 4 5
2004 2006 2008 2010 2012

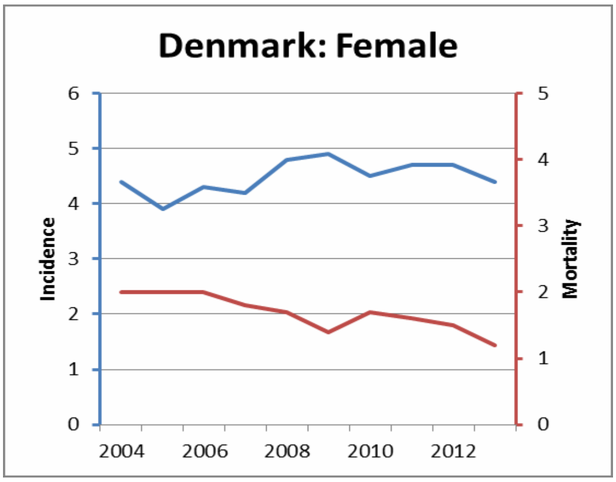
Denmark: Female
Incidence
Mortality
0 1 2 3 4 5 6
0 1 2 3 4 5
2004 2006 2008 2010 2012

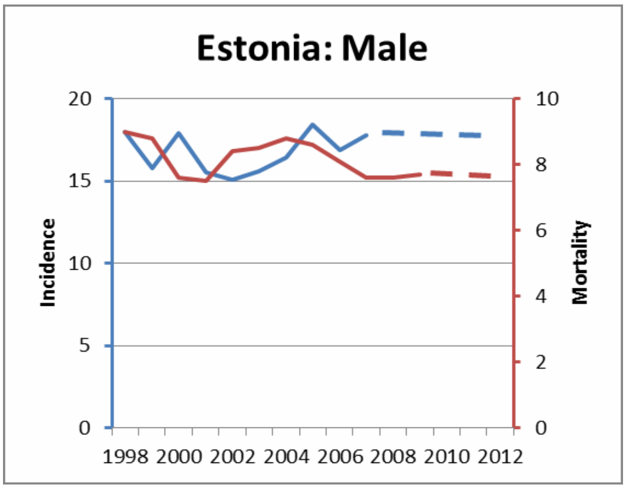
Estonia: Male
Incidence
Mortality
0 5 10 15 20
0 2 4 6 8 10
1998 2000 2002 2004 2006 2008 2010 2012

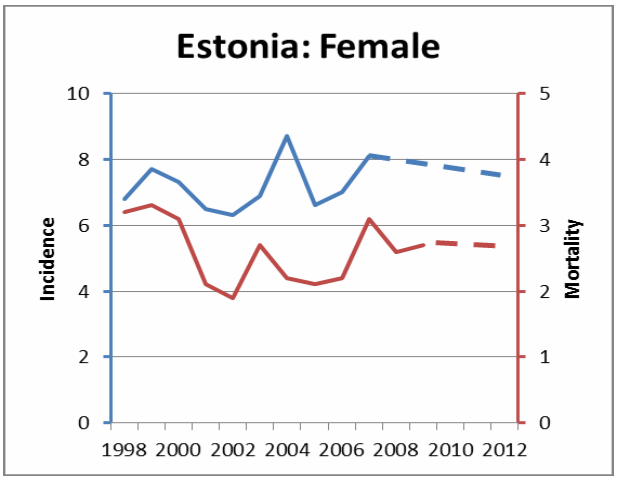
Estonia: Female
Incidence
Mortality
0 2 4 6 8 10
0 1 2 3 4 5
1998 2000 2002 2004 2006 2008 2010 2012

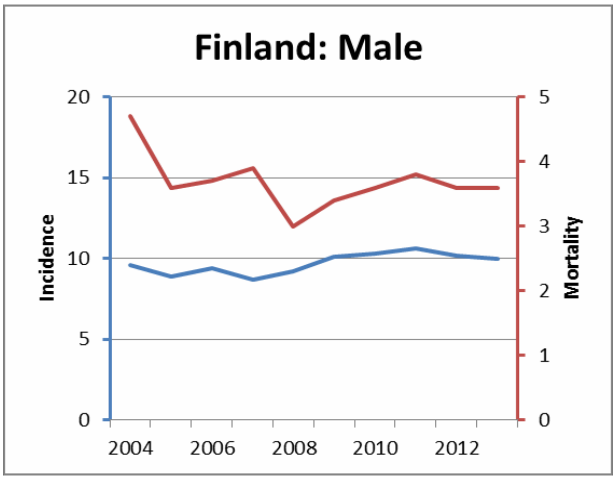
Finland: Male
Incidence
Mortality
0 5 10 15 20
0 1 2 3 4 5
2004 2006 2008 2010 2012

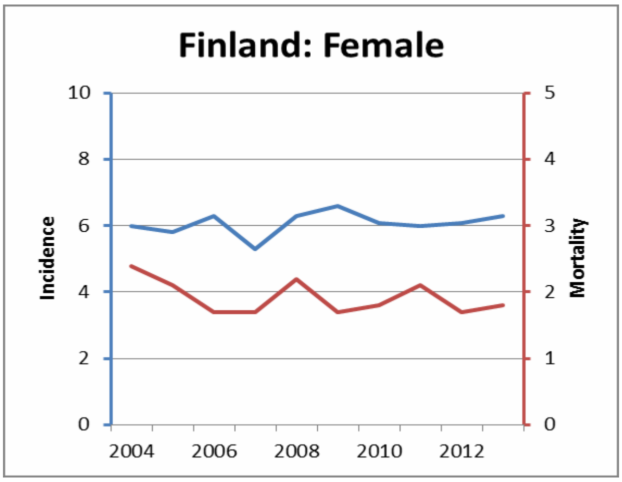
Finland: Female
Incidence
Mortality
0 2 4 6 8 10
0 1 2 3 4 5
2004 2006 2008 2010 2012

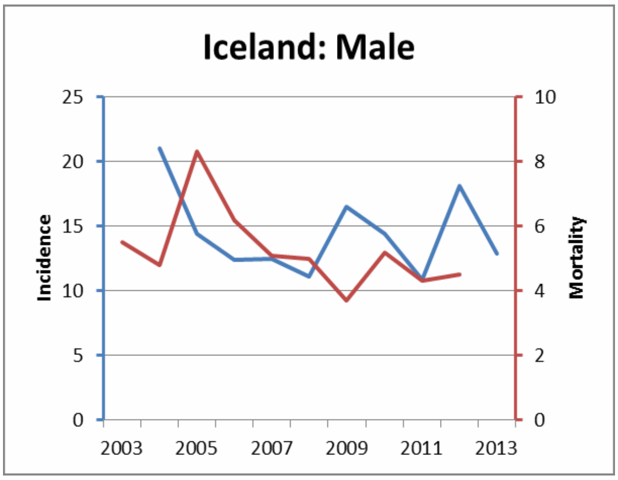
Iceland: Male
Incidence
Mortality
25
20
15
10
5
0
10
8
6
4
2
0
2003
2005
2007
2009
2011
2013

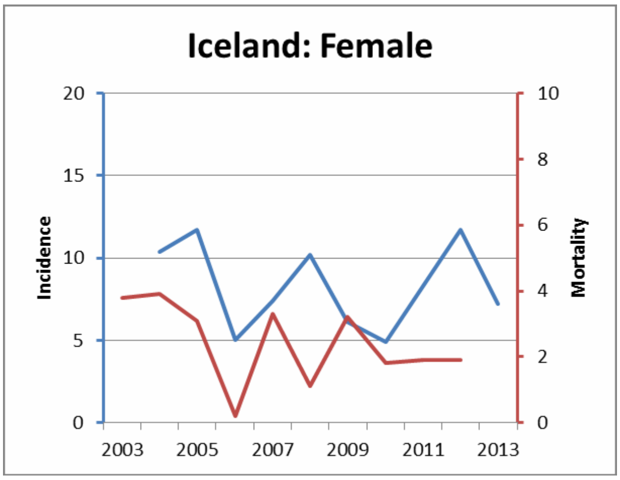
Iceland: Female
Incidence
Mortality
20
15
10
5
0
10
8
6
4
2
0
2003
2005
2007
2009
2011
2013

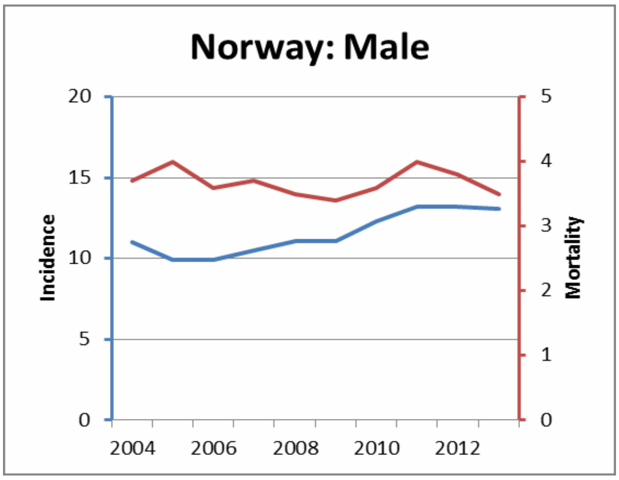
Norway: Male
Incidence
Mortality
20
15
10
5
0
5
4
3
2
1
0
2004
2006
2008
2010
2012

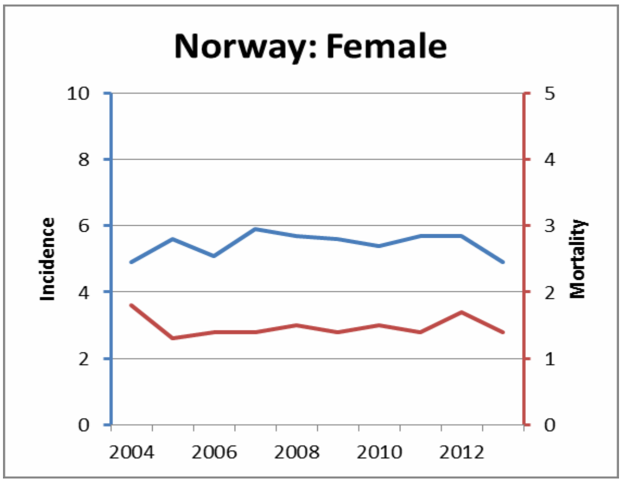
Norway: Female
Incidence
Mortality
10
8
6
4
2
0
5
4
3
2
1
0
2004
2006
2008
2010
2012

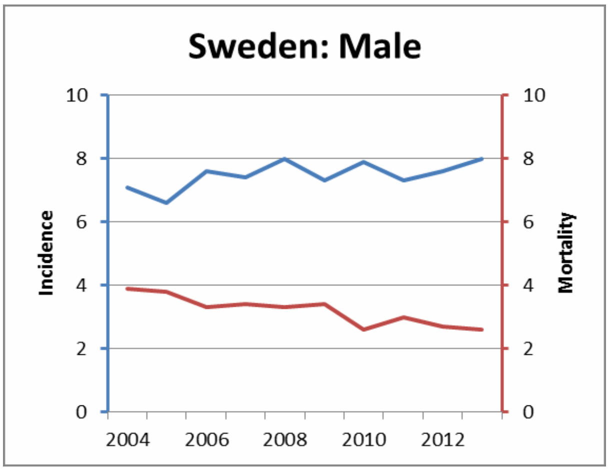
Sweden: Male
Incidence
Mortality
10
8
6
4
2
0
10
8
6
4
2
0
2004
2006
2008
2010
2012

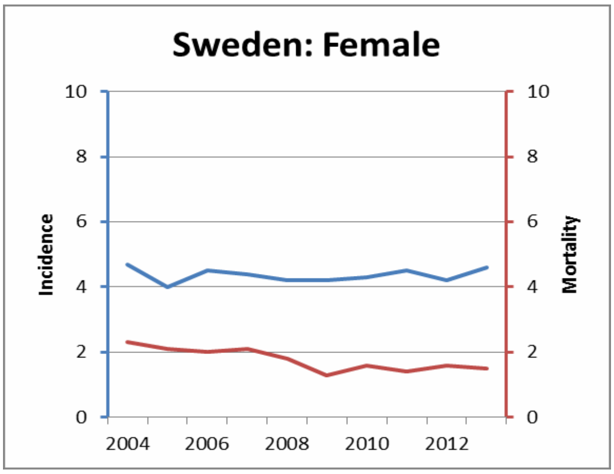
Sweden: Female
Incidence
Mortality
10
8
6
4
2
0
10
8
6
4
2
0
2004
2006
2008
2010
2012

6). Western Europe

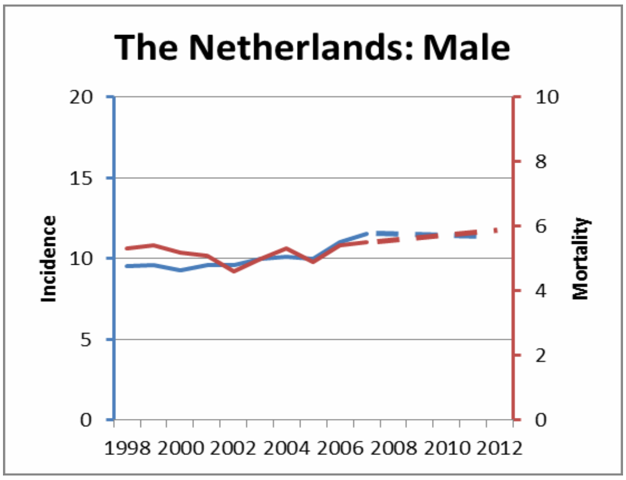

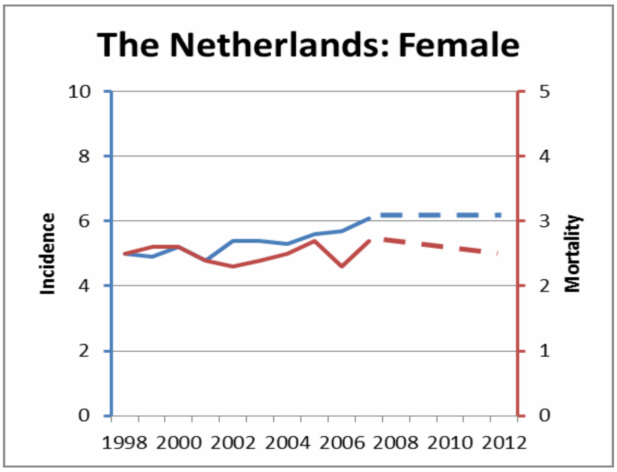

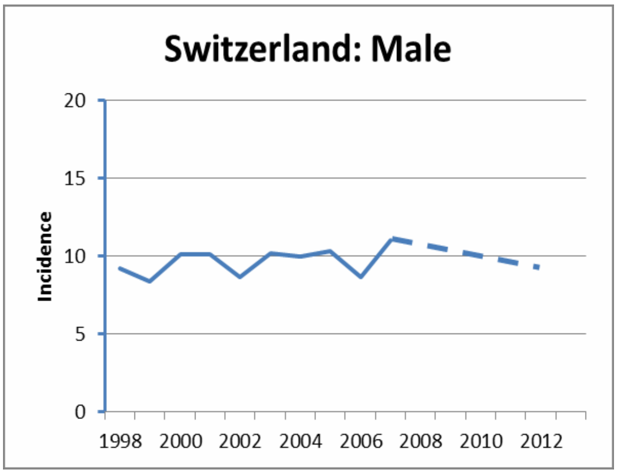

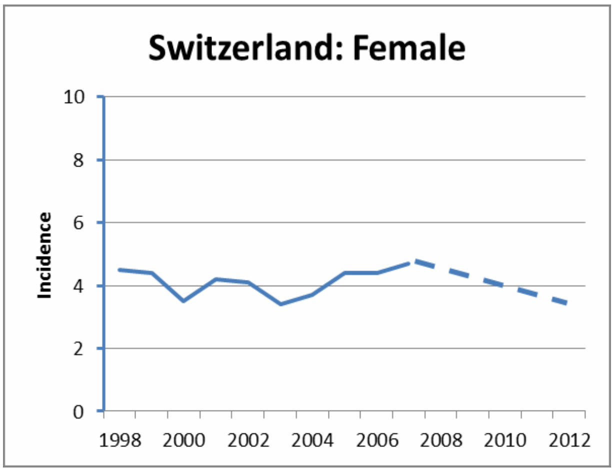

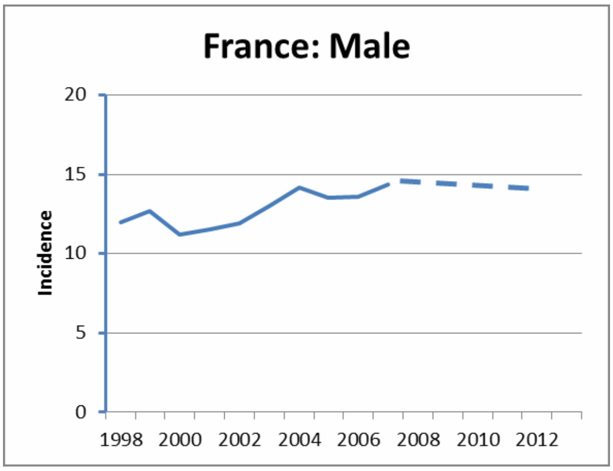

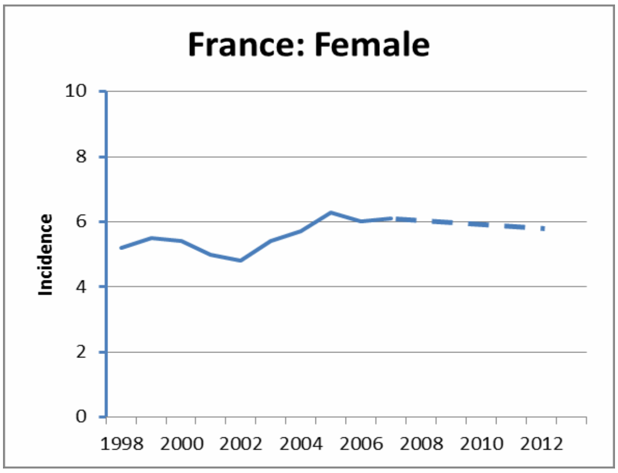

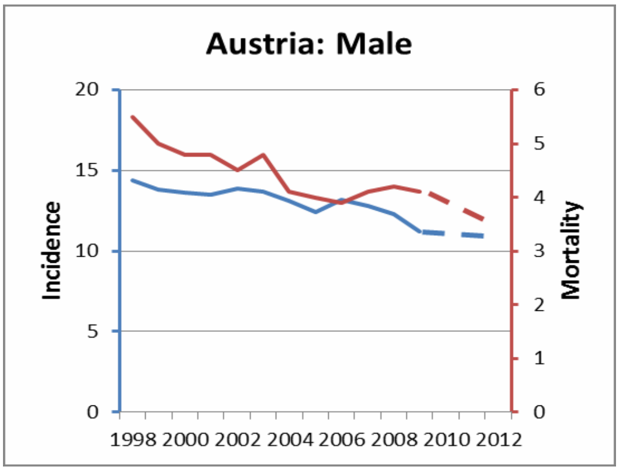
Austria: Male
Incidence
Mortality
20
15
10
5
0
6
5
4
3
2
1
0
1998 2000 2002 2004 2006 2008 2010 2012

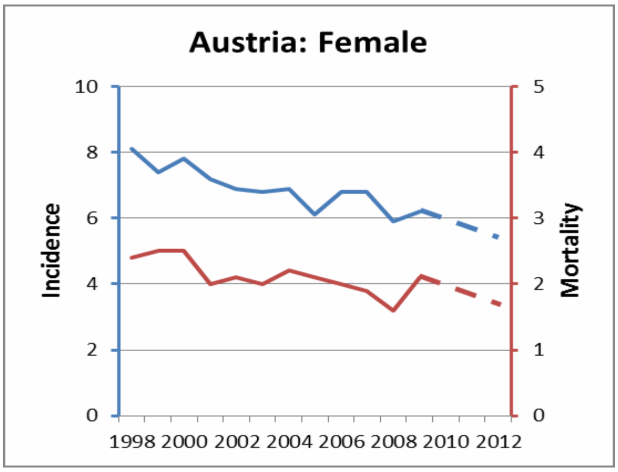
Austria: Female
Incidence
Mortality
10
8
6
4
2
0
5
4
3
2
1
0
1998 2000 2002 2004 2006 2008 2010 2012

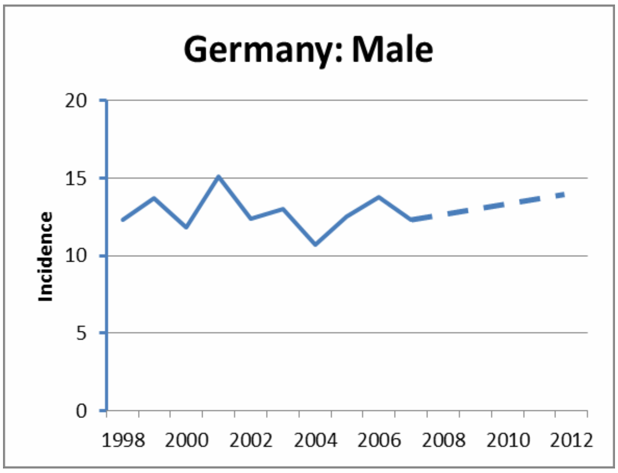
Germany: Male
Incidence
20
15
10
5
0
1998 2000 2002 2004 2006 2008 2010 2012

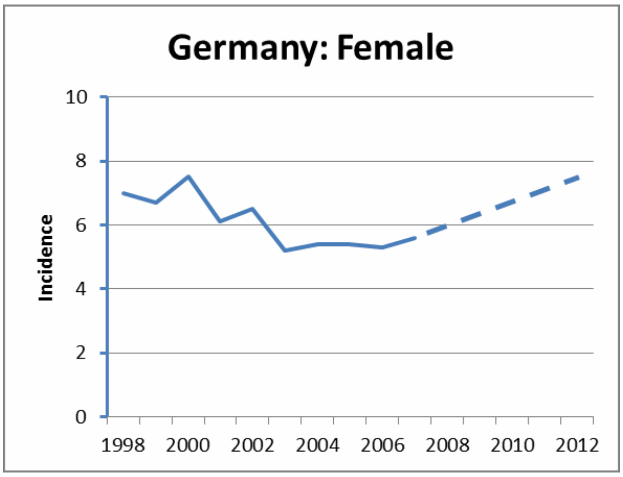
Germany: Female
Incidence
10
8
6
4
2
0
1998 2000 2002 2004 2006 2008 2010 2012

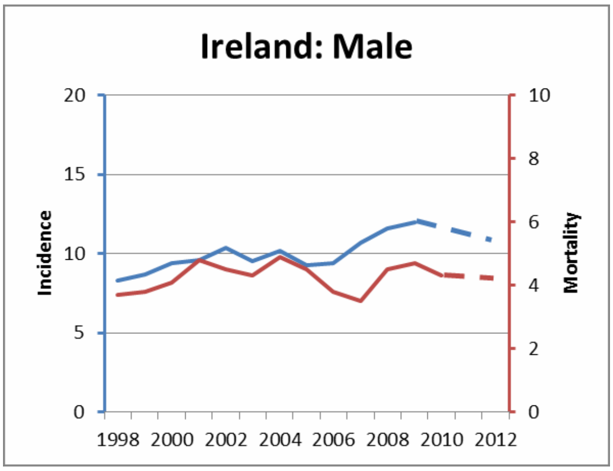
Ireland: Male
Incidence
Mortality
20
15
10
5
0
10
8
6
4
2
0
1998 2000 2002 2004 2006 2008 2010 2012

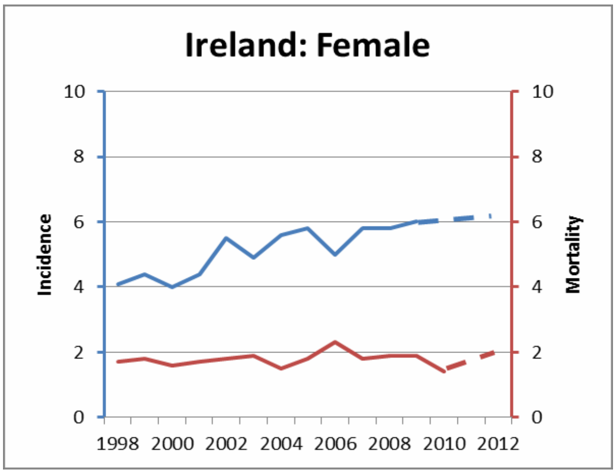
Ireland: Female
Incidence
Mortality
10
8
6
4
2
0
10
8
6
4
2
0
1998 2000 2002 2004 2006 2008 2010 2012

7). Southern Europe

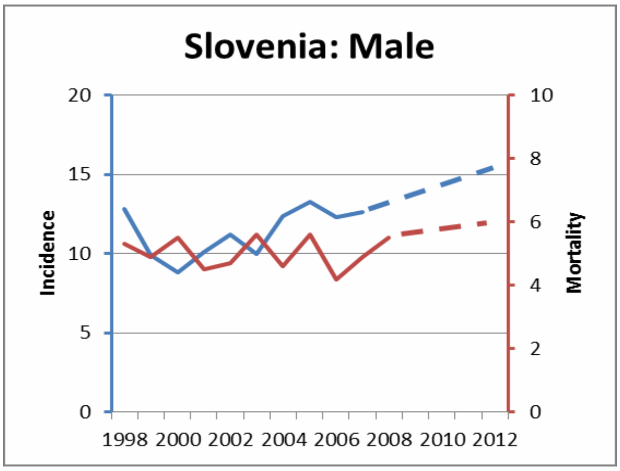

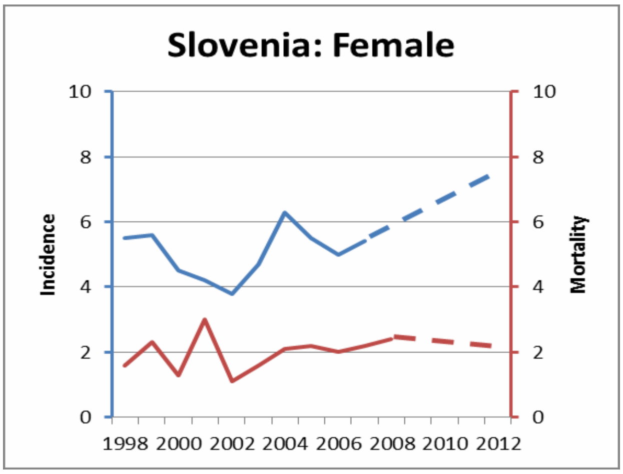

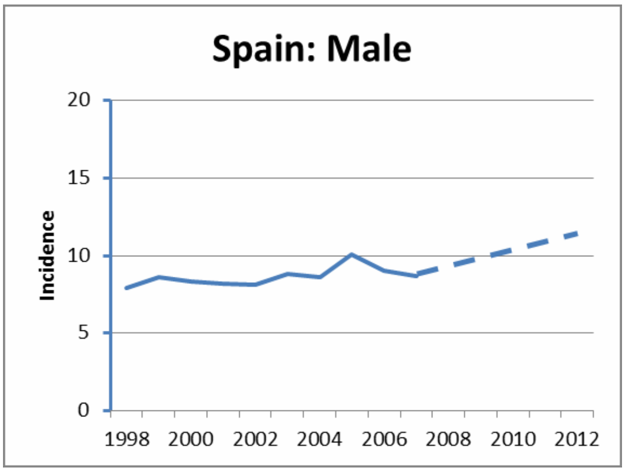

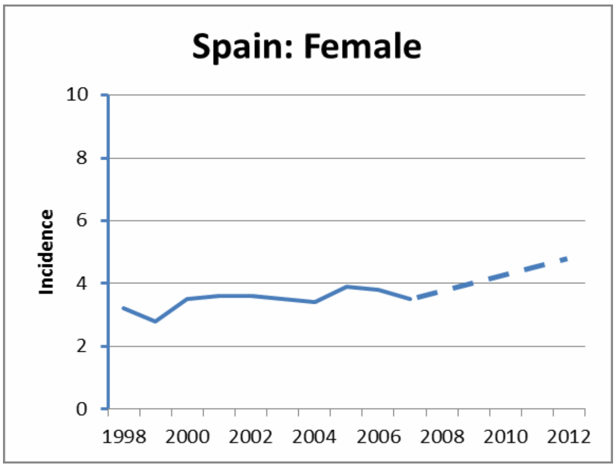

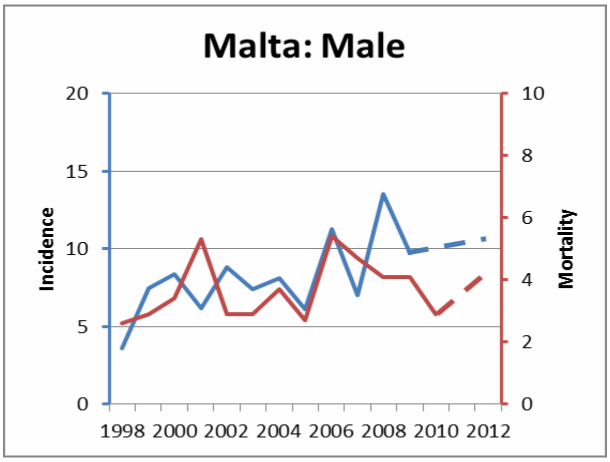

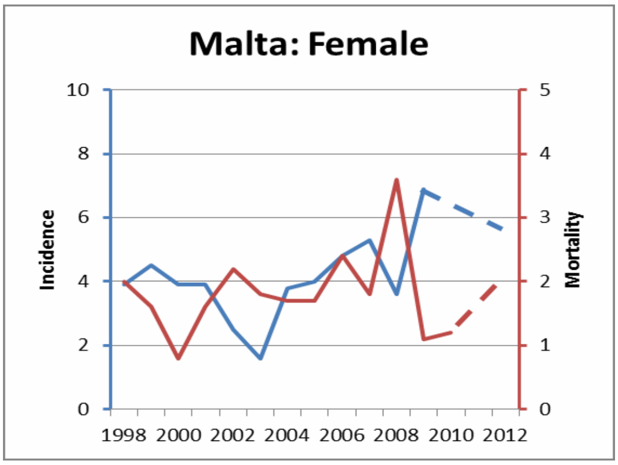

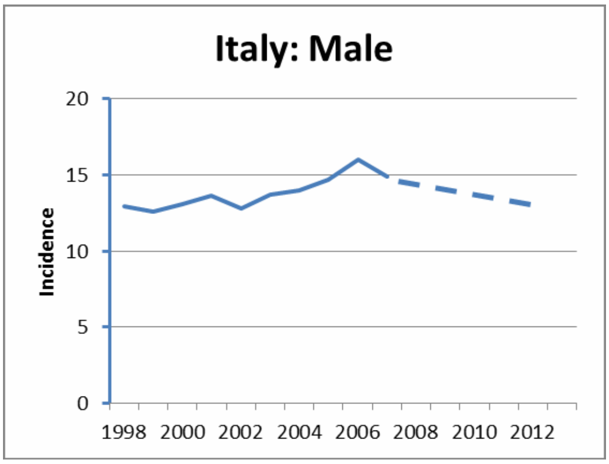
Italy: Male
Incidence
20
15
10
5
0
1998 2000 2002 2004 2006 2008 2010 2012

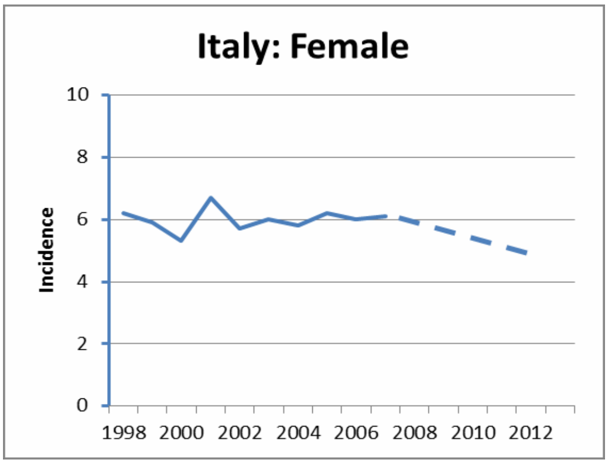
Italy: Female
Incidence
10
8
6
4
2
0
1998 2000 2002 2004 2006 2008 2010 2012

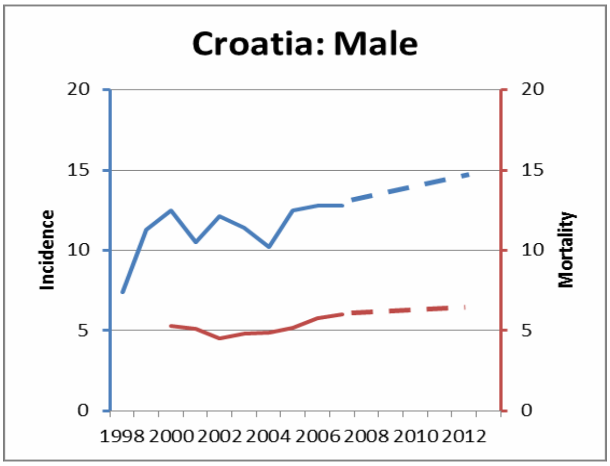
Croatia: Male
Incidence
Mortality
20
15
10
5
0
1998 2000 2002 2004 2006 2008 2010 2012

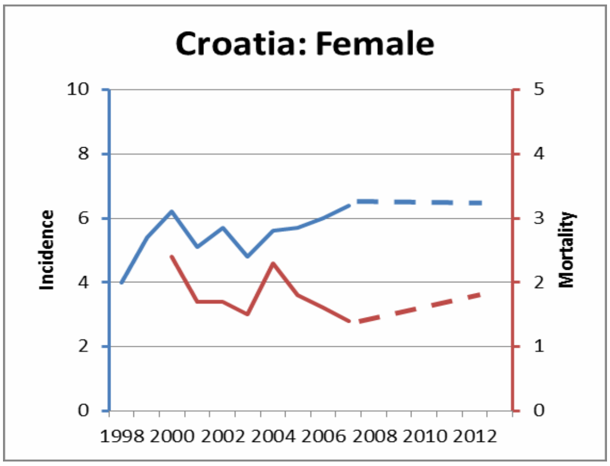
Croatia: Female
Incidence
Mortality
10
8
6
4
2
0
5
4
3
2
1
0
1998 2000 2002 2004 2006 2008 2010 2012

8). Eastern Europe

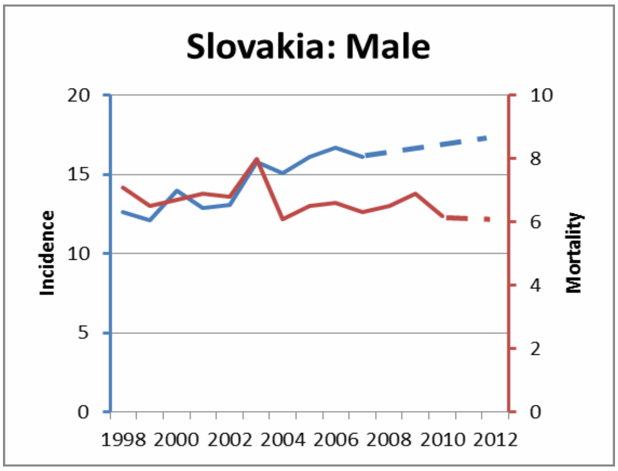

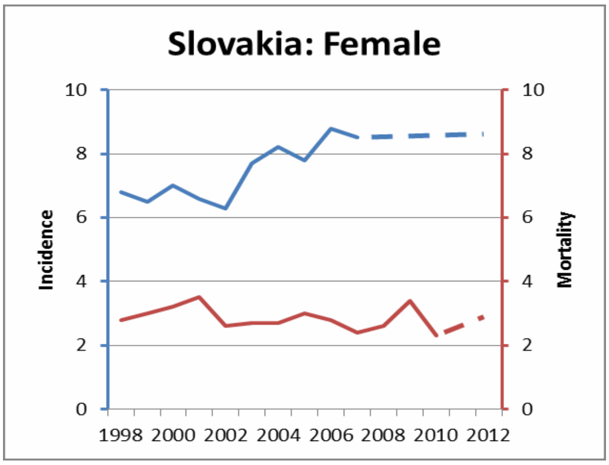

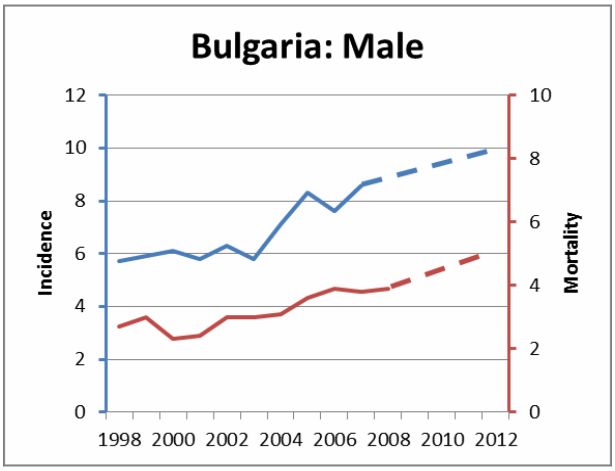

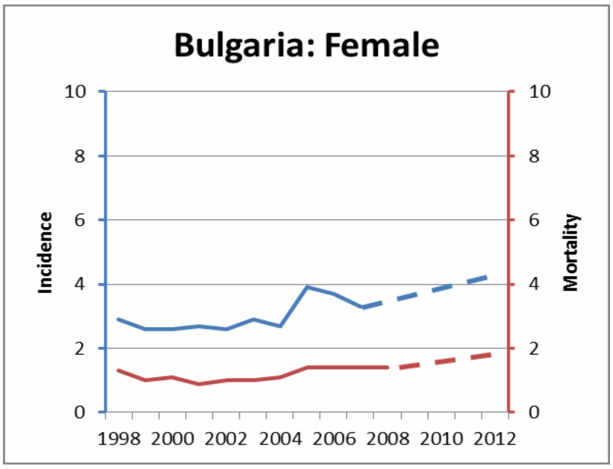

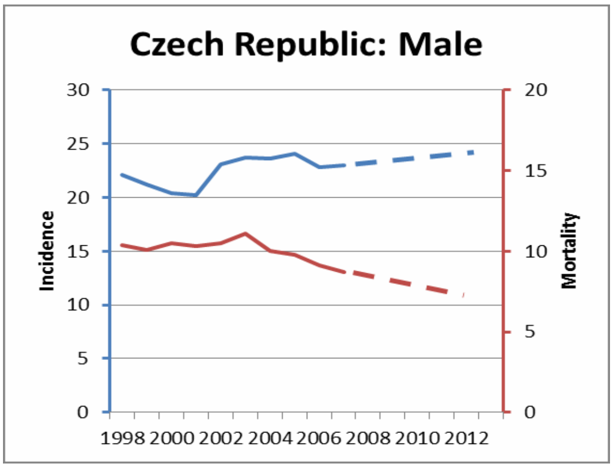

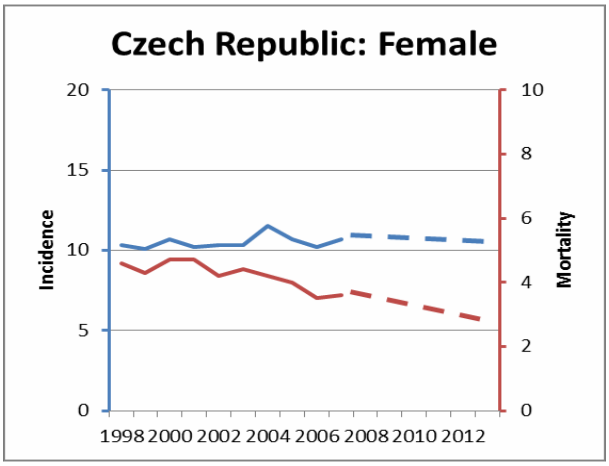

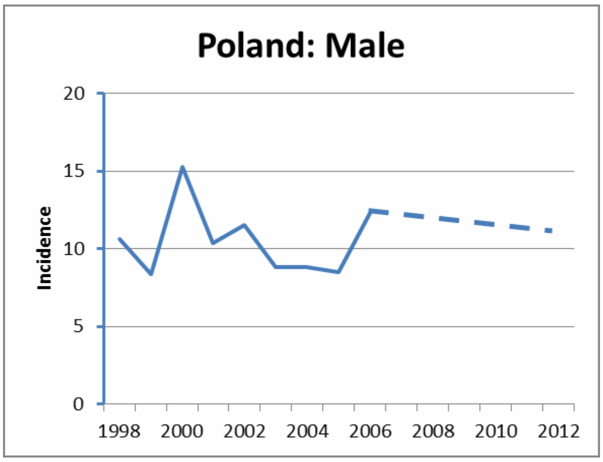
Poland: Male
Incidence
20
15
10
5
0
1998
2000
2002
2004
2006
2008
2010
2012

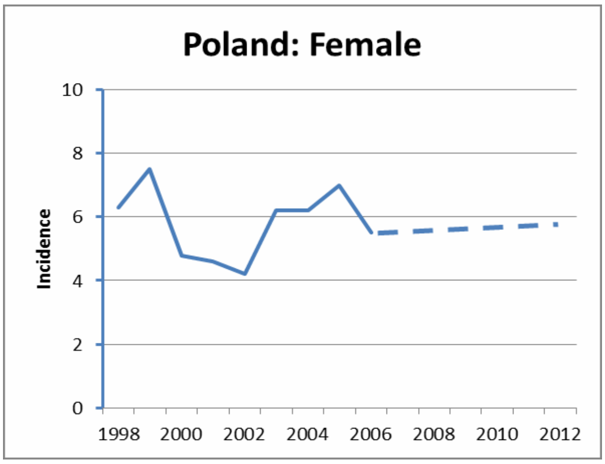
Poland: Female
Incidence
10
8
6
4
2
0
1998
2000
2002
2004
2006
2008
2010
2012

Supplementary Figure 2 Findings from the joinpoint regression analysis of the global incidence rates of kidney cancer (Left: Male, Right: Female)

1) **Latin America and the Caribbean**

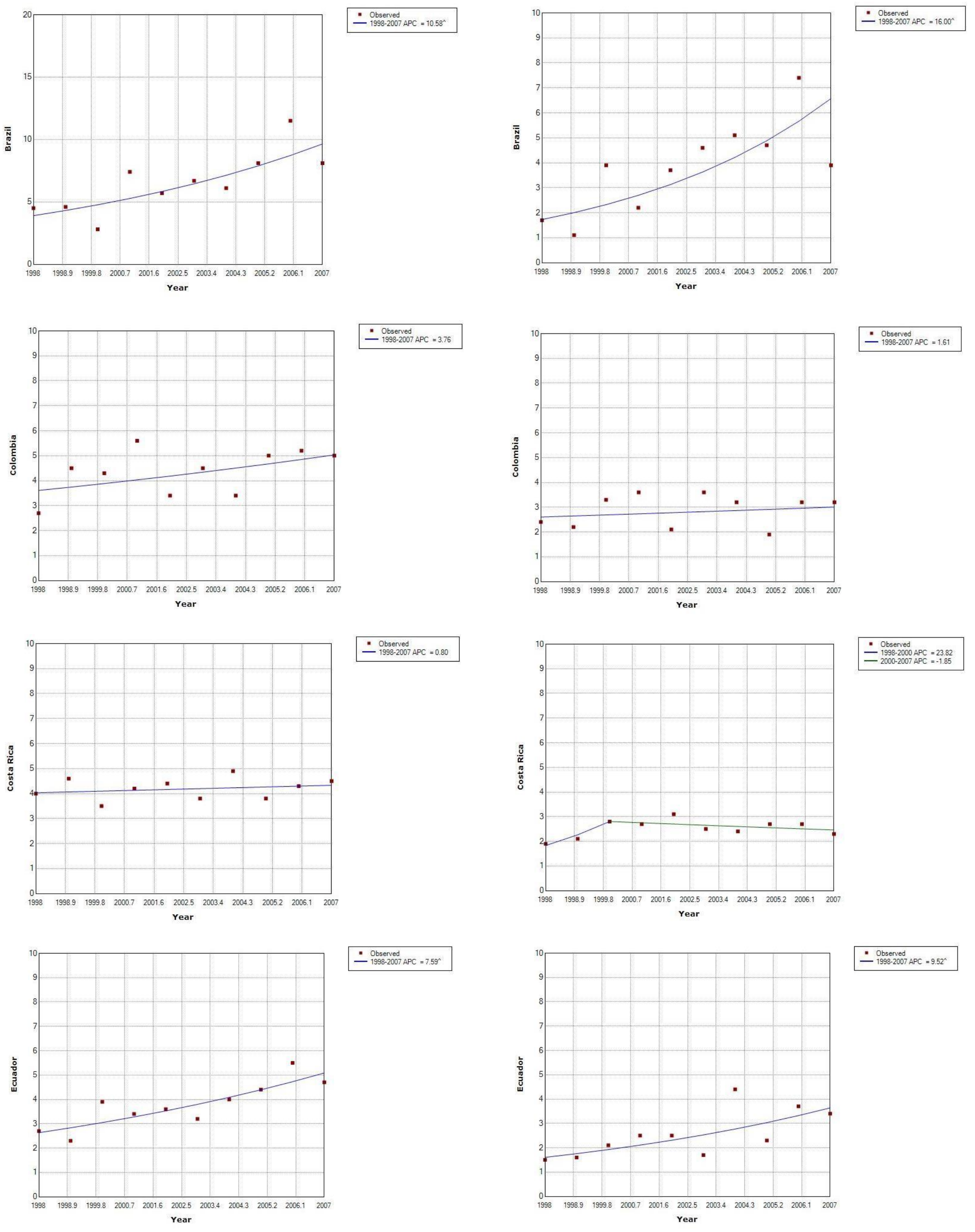

2) Northern America

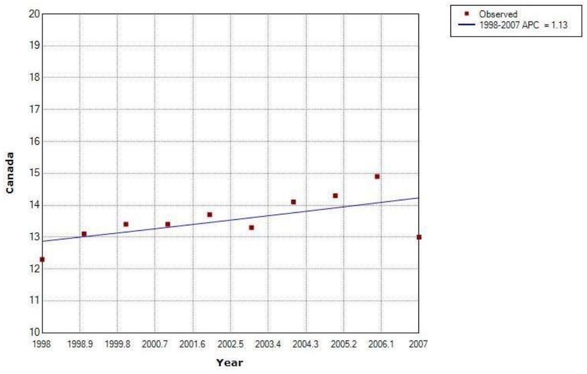

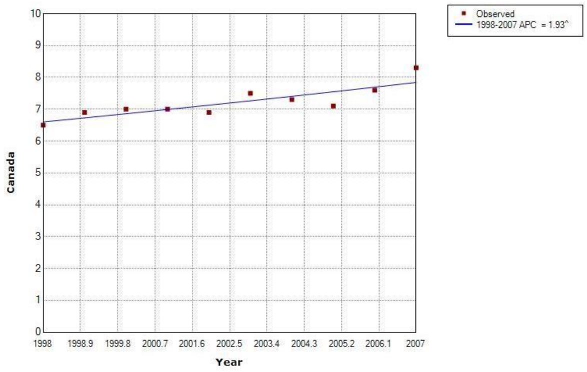

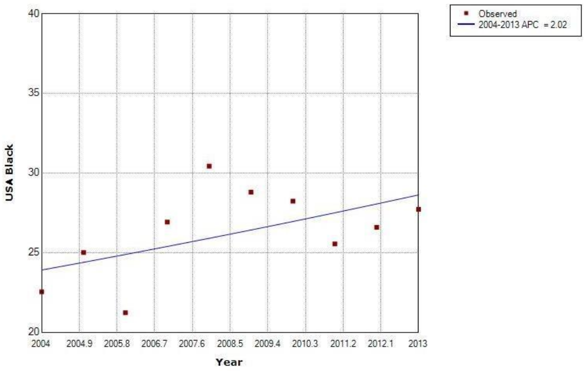

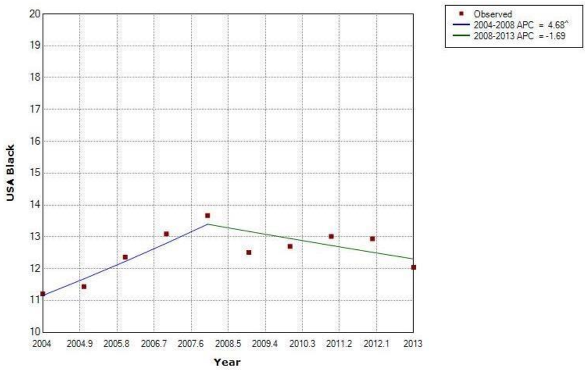

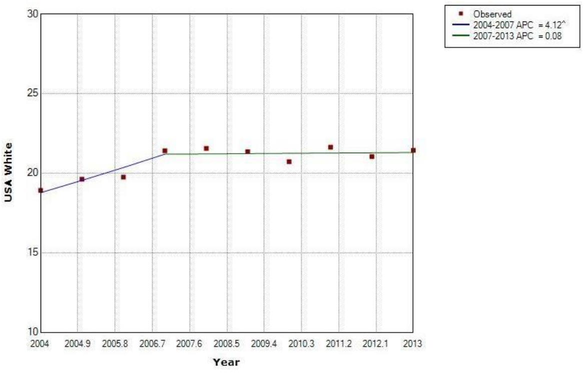

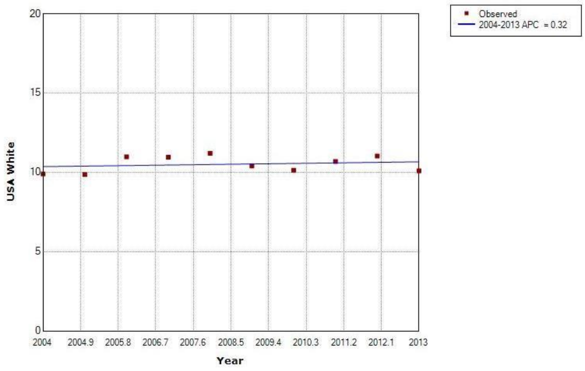

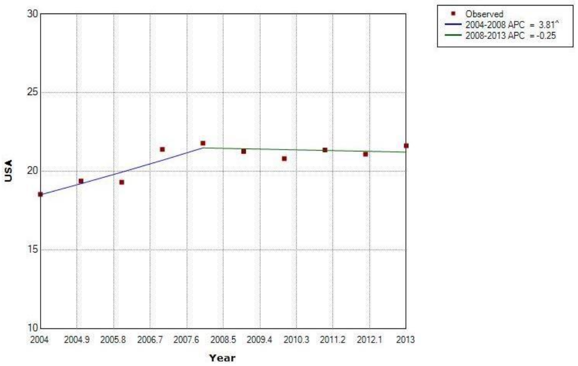

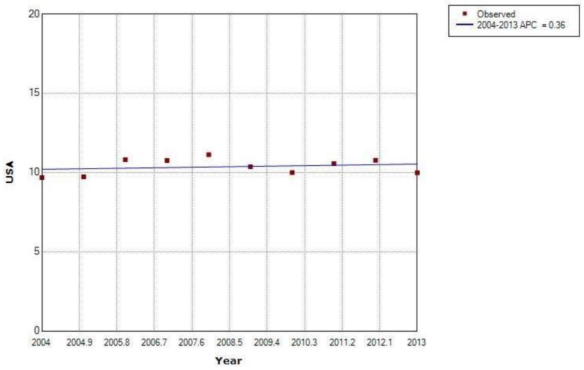

3) Asia

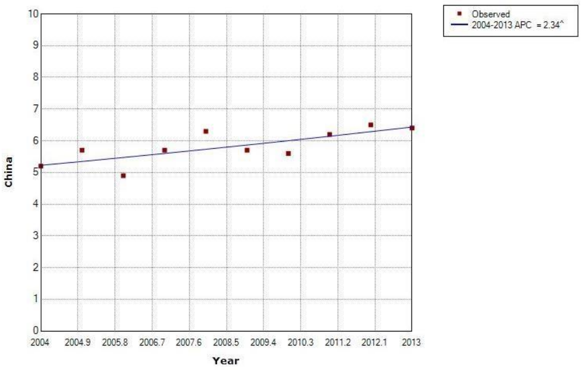
Observed
2004-2013 APC = 2.34^
China
Year

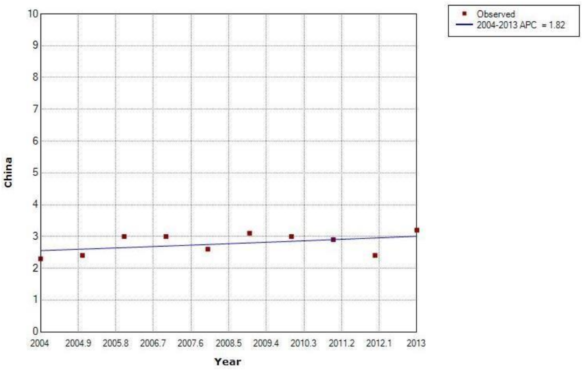
Observed
2004-2013 APC = 1.82
China
Year

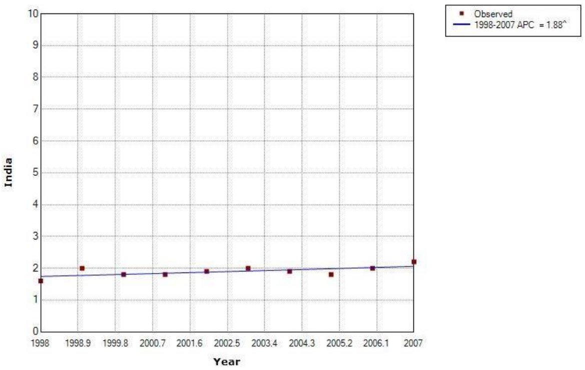
Observed
1998-2007 APC = 1.88^
India
Year

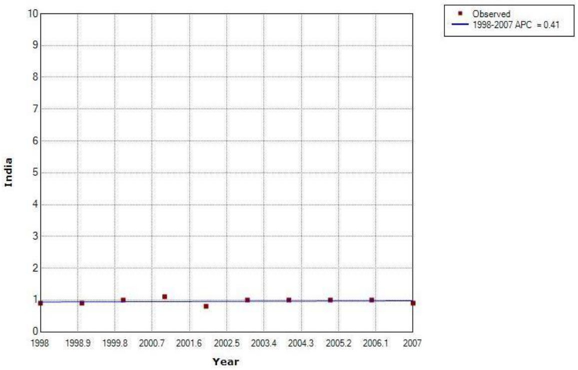
Observed
1998-2007 APC = 0.41
India
Year

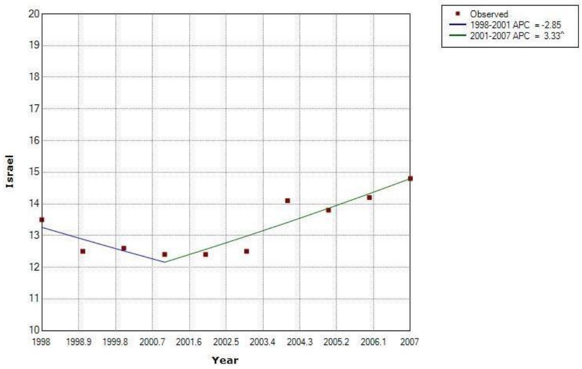
Observed
1998-2001 APC = -2.85
2001-2007 APC = 3.33^
Israel
Year

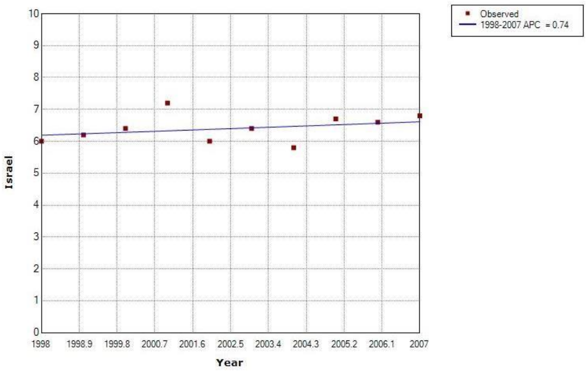
Observed
1998-2007 APC = 0.74
Israel
Year

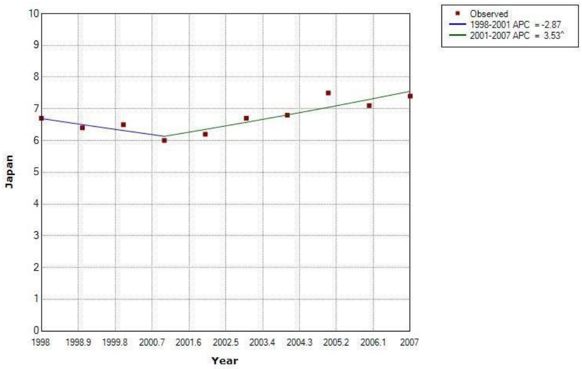
Observed
1998-2001 APC = -2.87
2001-2007 APC = 3.53^
Japan
Year

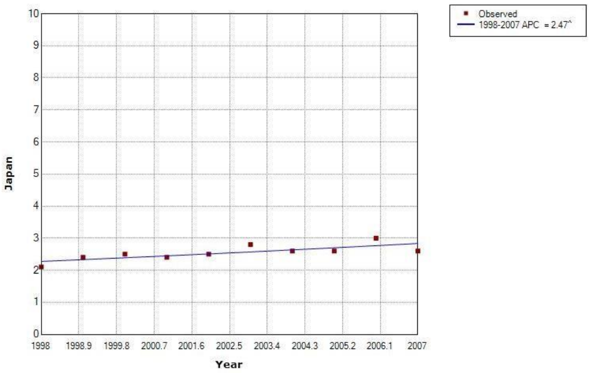
Observed
1998-2007 APC = 2.47^
Japan
Year

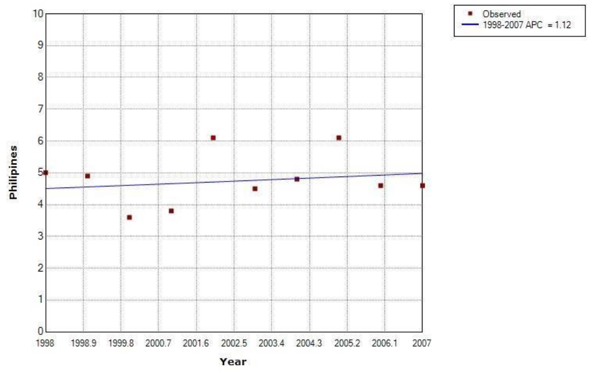
Observed
1998-2007 APC = 1.12
Philipines
Year

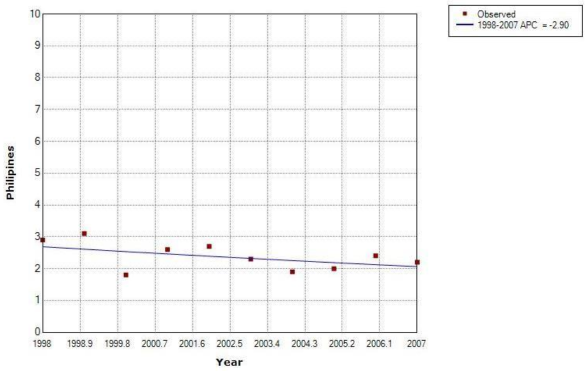
Observed
1998-2007 APC = -2.90
Philipines
Year

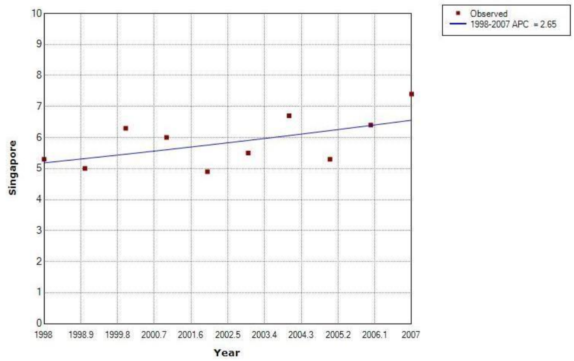
Observed
1998-2007 APC = 2.65
Singapore
Year

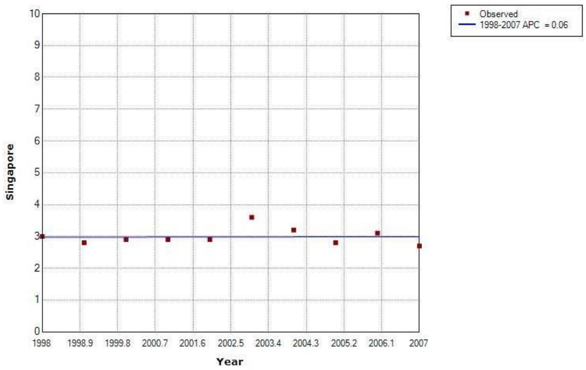
Observed
1998-2007 APC = 0.06
Singapore
Year

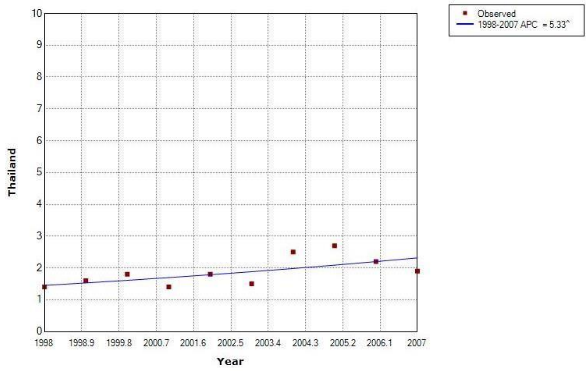
Observed
1998-2007 APC = 5.33^
Thailand
Year

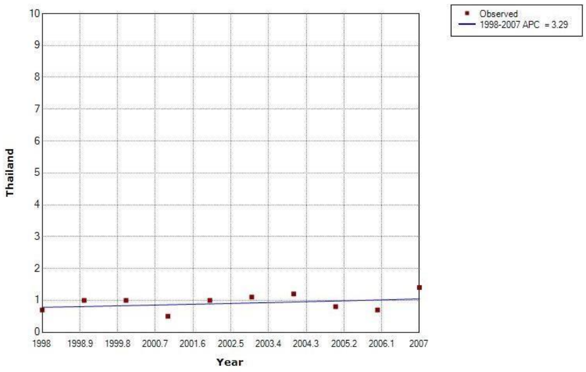
Observed
1998-2007 APC = 3.29
Thailand
Year

4) Oceania

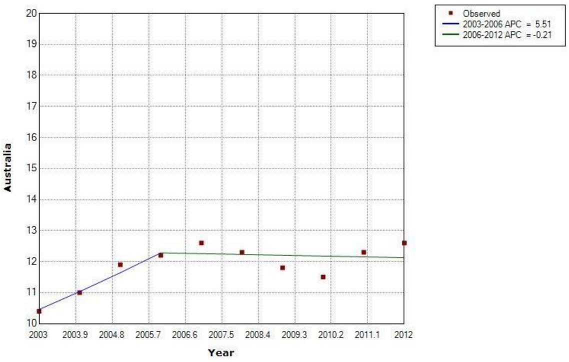
Observed
2003-2006 APC = 5.51
2006-2012 APC = -0.21
Australia
Year

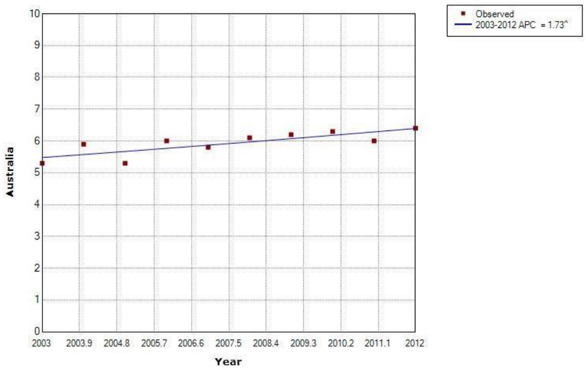
Observed
2003-2012 APC = 1.73^
Australia
Year

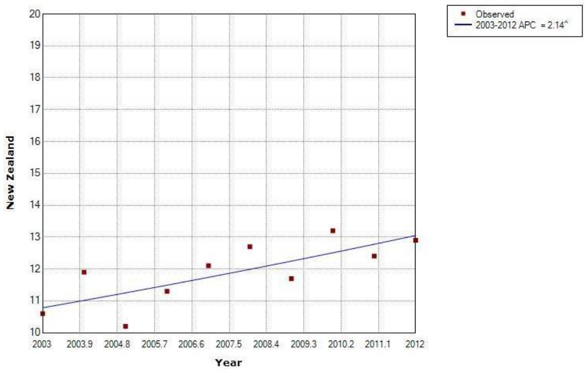

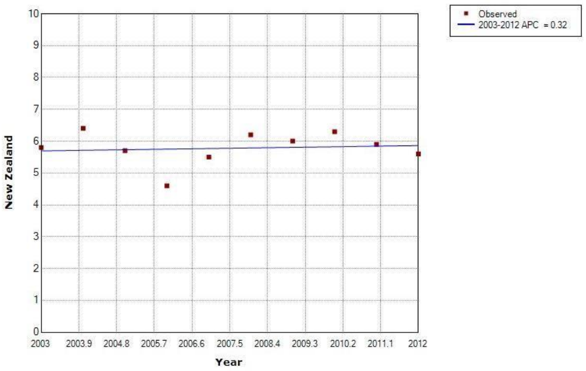

5). Northern Europe

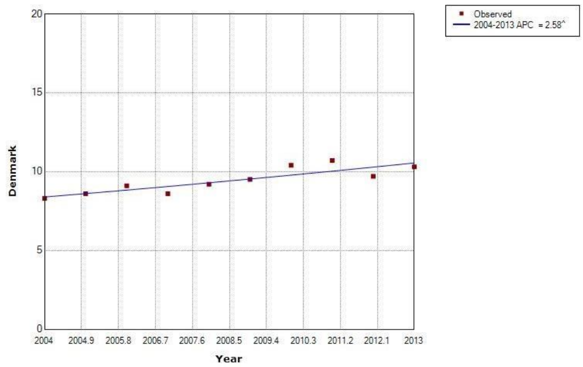

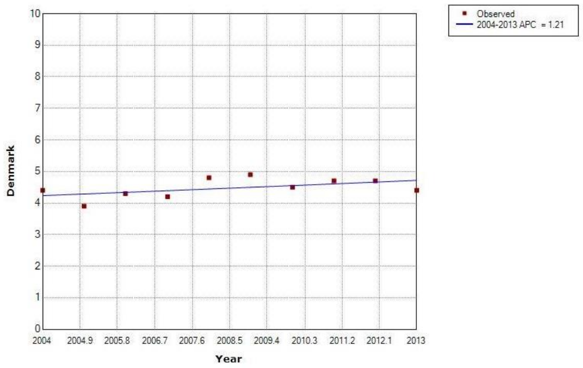

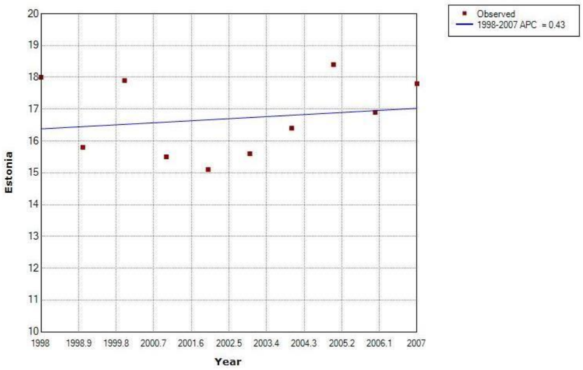

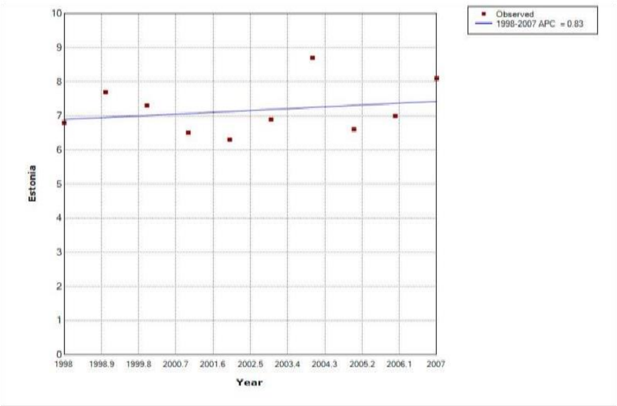

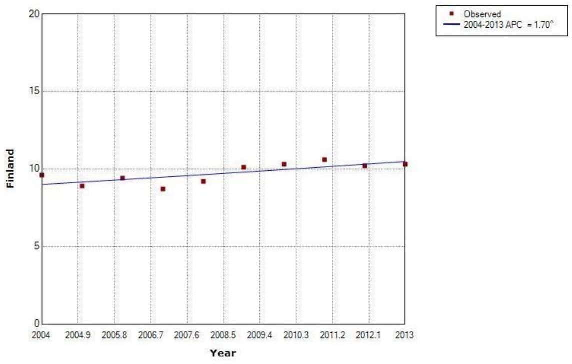

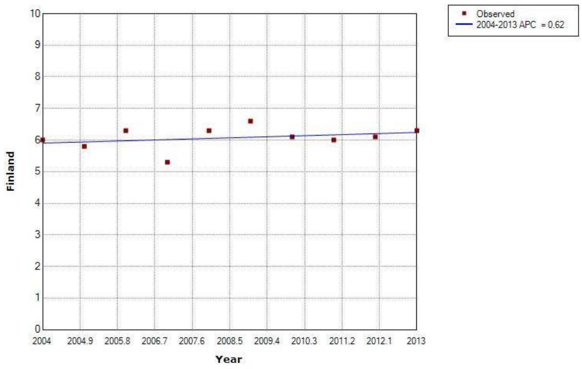

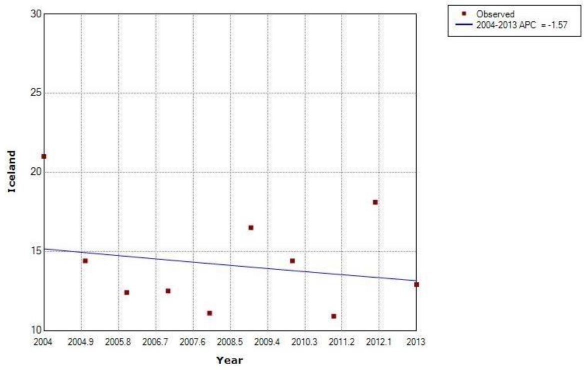
Observed
2004-2013 APC = -1.57
Iceland
Year
2004 2004.9 2005.8 2006.7 2007.6 2008.5 2009.4 2010.3 2011.2 2012.1 2013

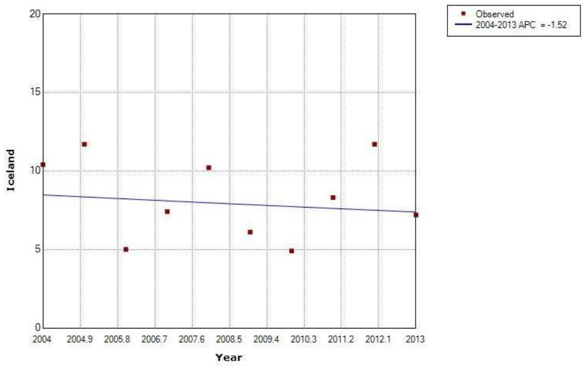
Observed
2004-2013 APC = -1.52
Iceland
Year
2004 2004.9 2005.8 2006.7 2007.6 2008.5 2009.4 2010.3 2011.2 2012.1 2013

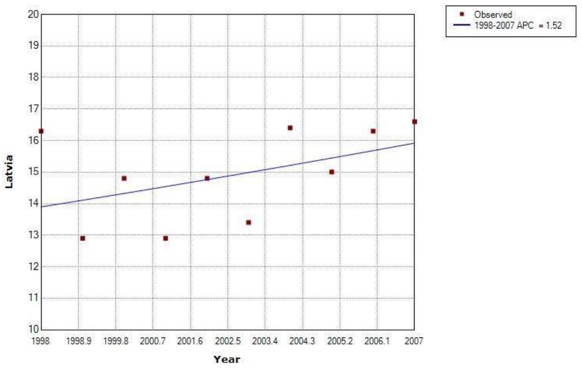
Observed
1998-2007 APC = 1.52
Latvia
Year
1998 1998.9 1999.8 2000.7 2001.6 2002.5 2003.4 2004.3 2005.2 2006.1 2007

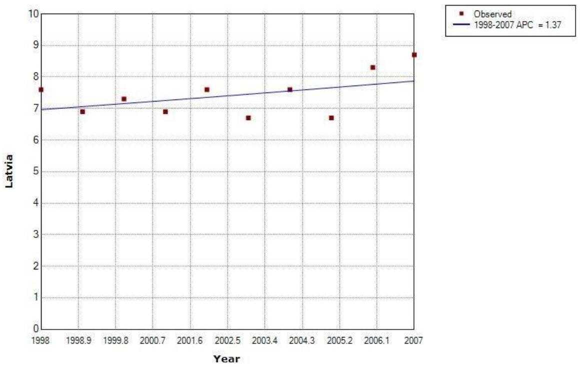
Observed
1998-2007 APC = 1.37
Latvia
Year
1998 1998.9 1999.8 2000.7 2001.6 2002.5 2003.4 2004.3 2005.2 2006.1 2007

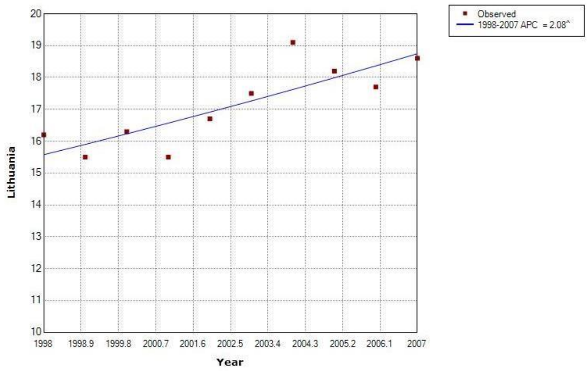
Observed
1998-2007 APC = 2.08^
Lithuania
Year
1998 1998.9 1999.8 2000.7 2001.6 2002.5 2003.4 2004.3 2005.2 2006.1 2007

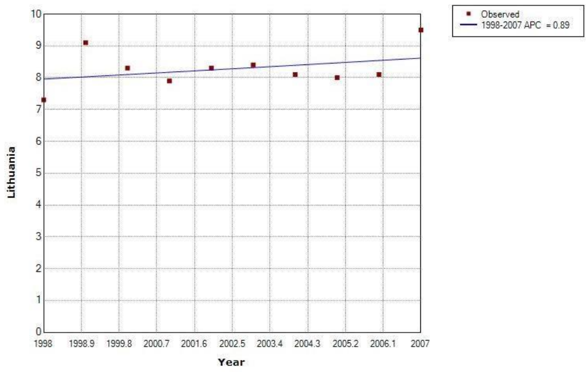
Observed
1998-2007 APC = 0.89
Lithuania
Year
1998 1998.9 1999.8 2000.7 2001.6 2002.5 2003.4 2004.3 2005.2 2006.1 2007

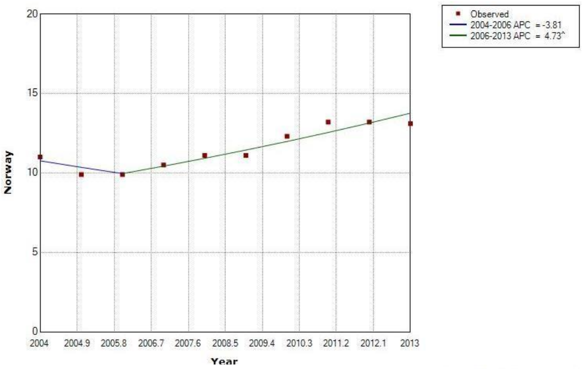
Observed
2004-2006 APC = -3.81
2006-2013 APC = 4.73^
Norway
Year
2004 2004.9 2005.8 2006.7 2007.6 2008.5 2009.4 2010.3 2011.2 2012.1 2013

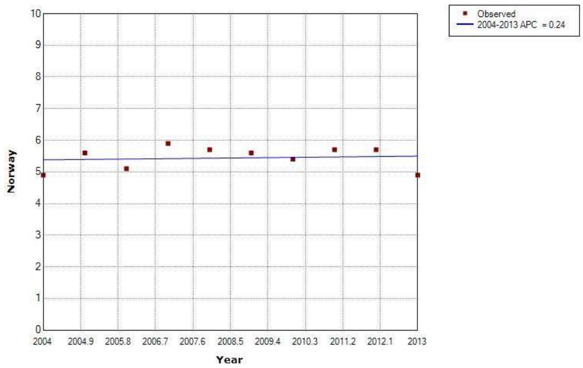
Observed
2004-2013 APC = 0.24
Norway
Year
2004 2004.9 2005.8 2006.7 2007.6 2008.5 2009.4 2010.3 2011.2 2012.1 2013

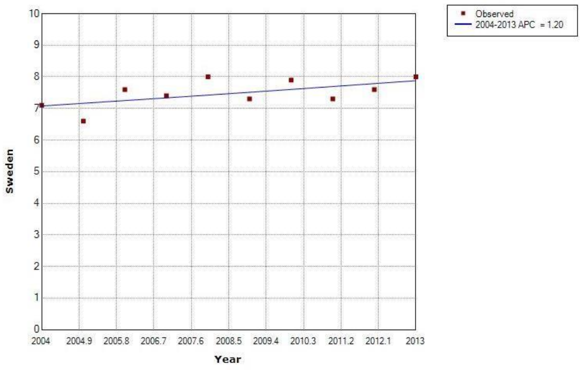
Observed
2004-2013 APC = 1.20
Sweden
Year
2004 2004.9 2005.8 2006.7 2007.6 2008.5 2009.4 2010.3 2011.2 2012.1 2013

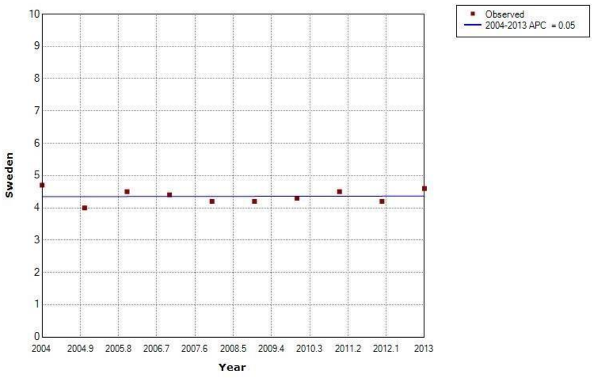
Observed
2004-2013 APC = 0.05
Sweden
Year
2004 2004.9 2005.8 2006.7 2007.6 2008.5 2009.4 2010.3 2011.2 2012.1 2013

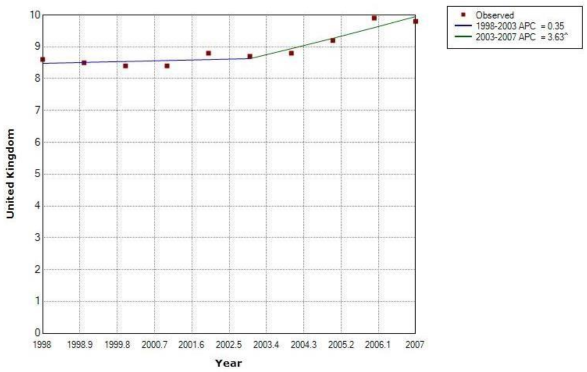

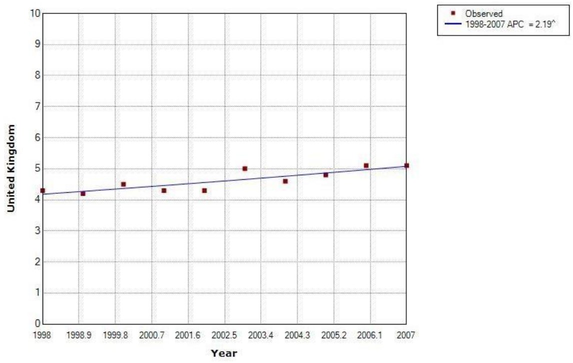

6) Western Europe

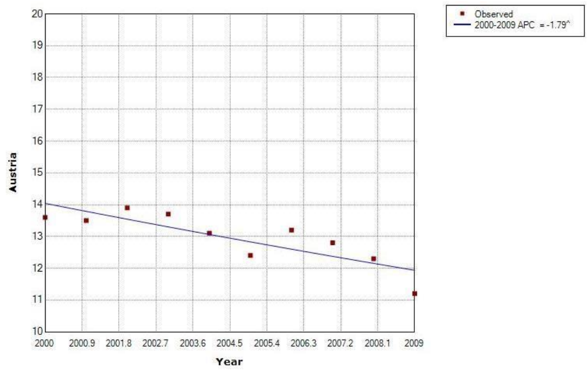

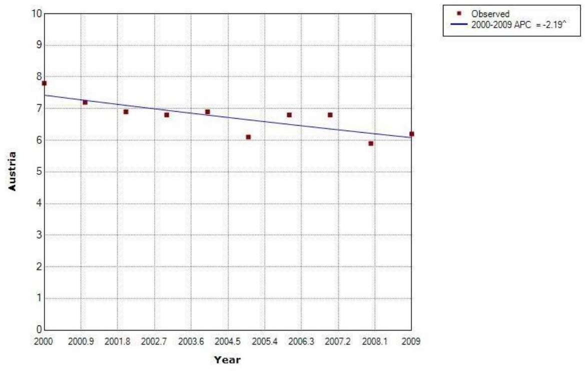

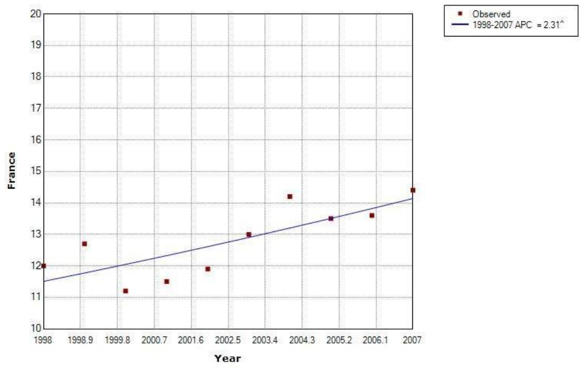

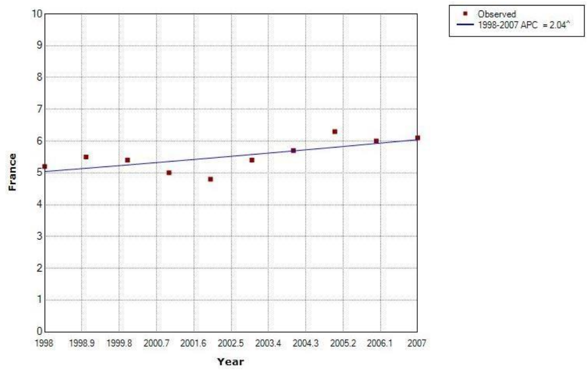

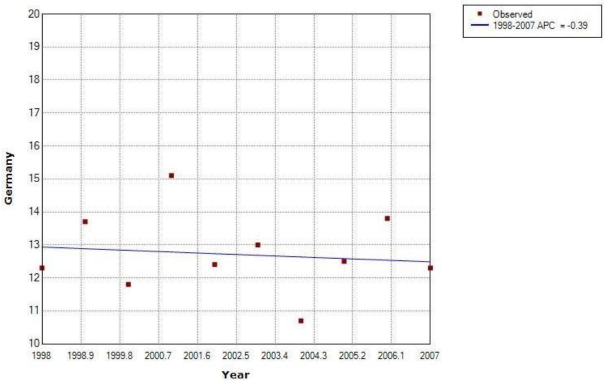
Observed
1998-2007 APC = -0.39
Germany
Year

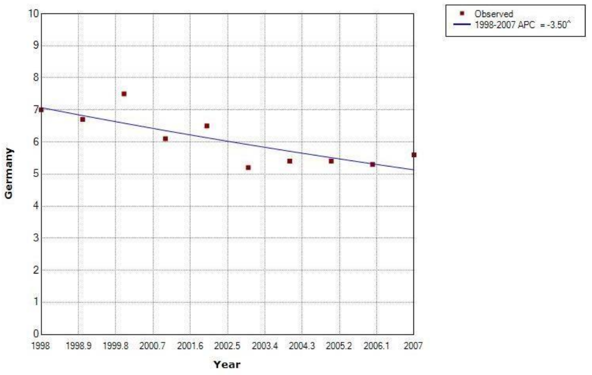
Observed
1998-2007 APC = -3.50^
Germany
Year

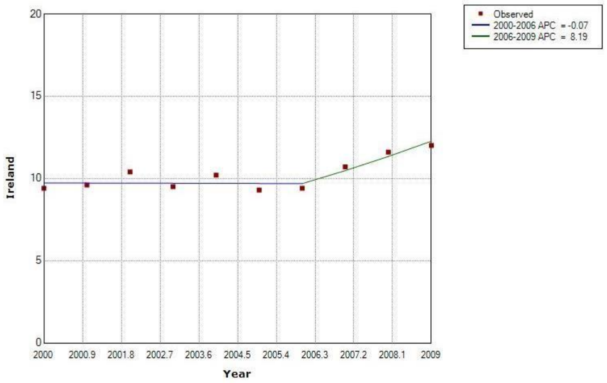
Observed
2000-2006 APC = -0.07
2006-2009 APC = 8.19
Ireland
Year

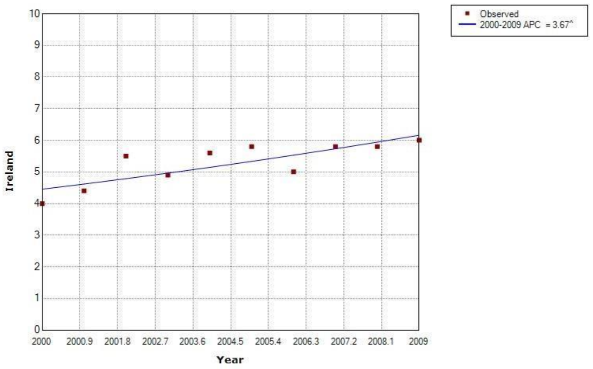
Observed
2000-2009 APC = 3.67^
Ireland
Year

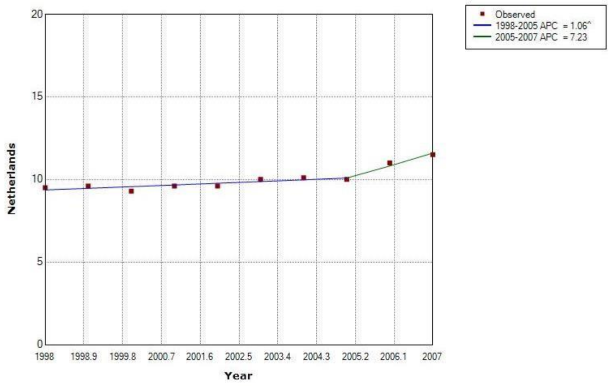
Observed
1998-2005 APC = 1.06^
2005-2007 APC = 7.23
Netherlands
Year

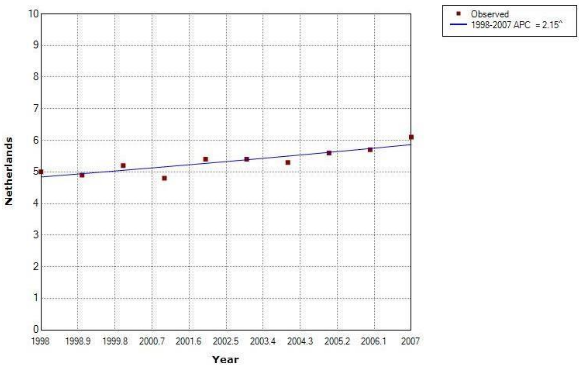
Observed
1998-2007 APC = 2.15^
Netherlands
Year

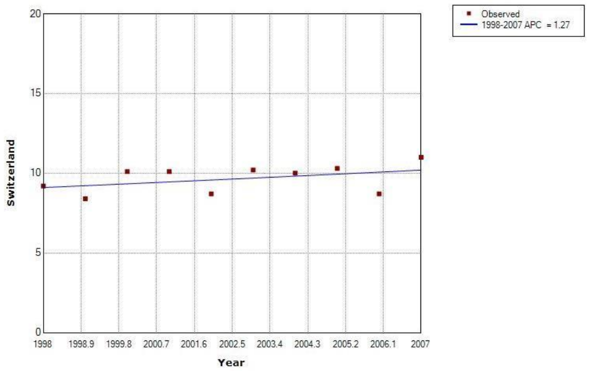
Observed
1998-2007 APC = 1.27
Switzerland
Year

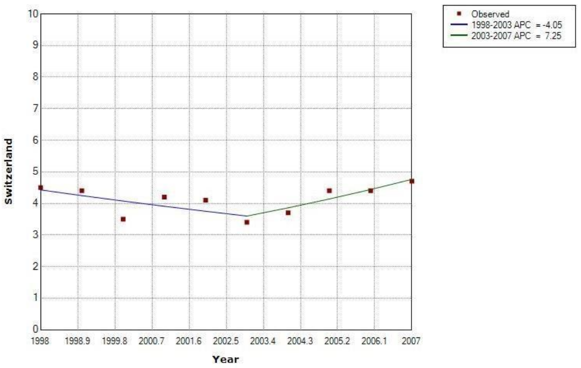
Observed
1998-2003 APC = -4.05
2003-2007 APC = 7.25
Switzerland
Year

7) Southern Europe

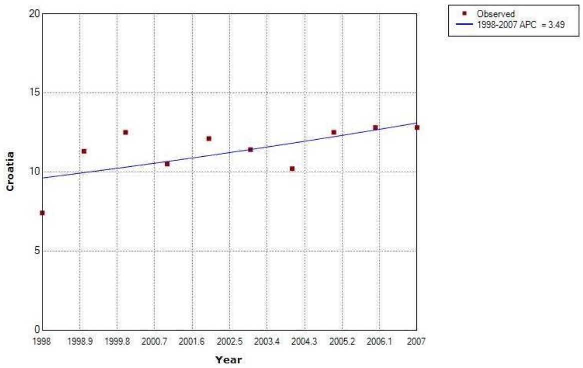

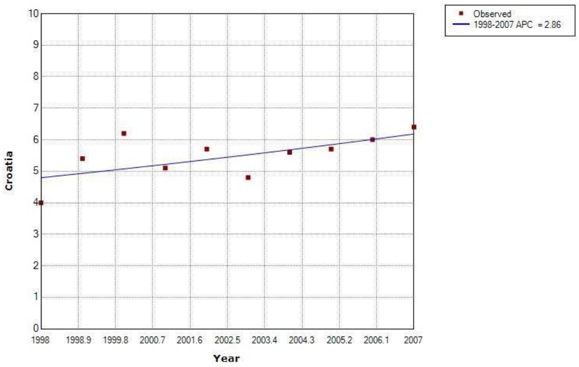

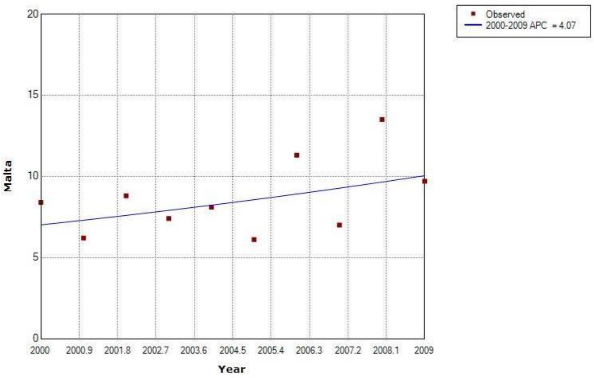

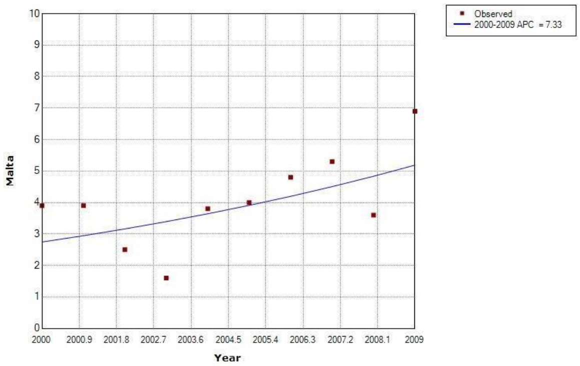

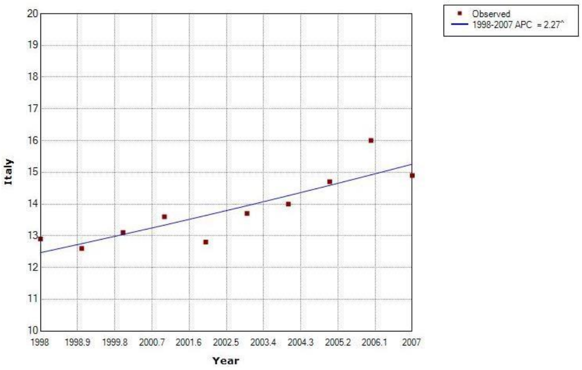

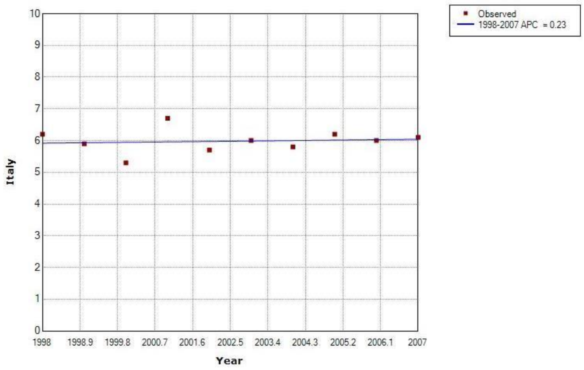

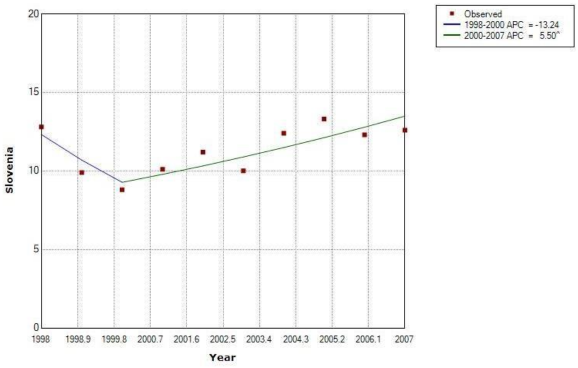

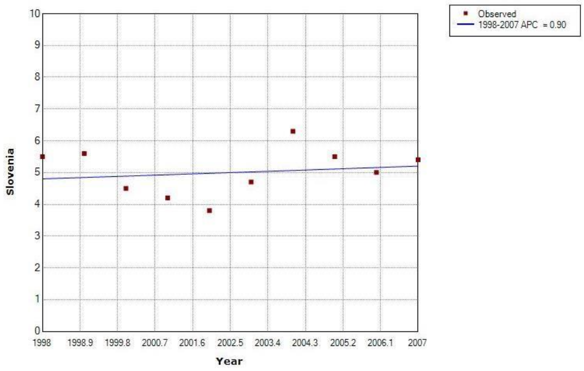

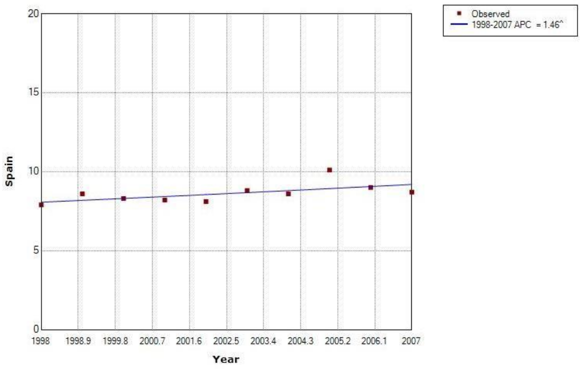

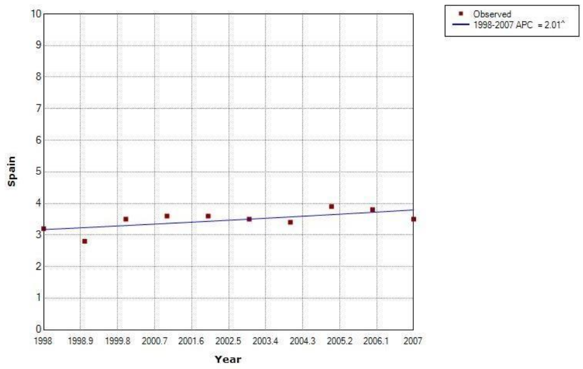

8) Eastern Europe

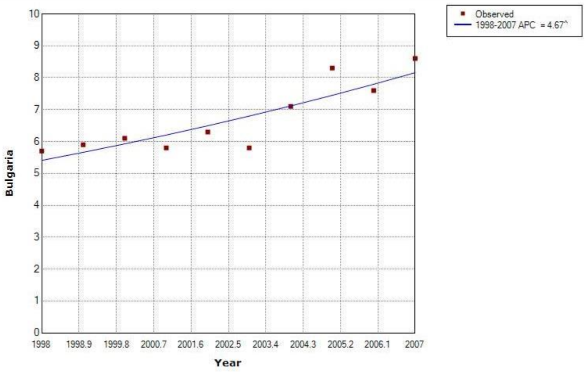

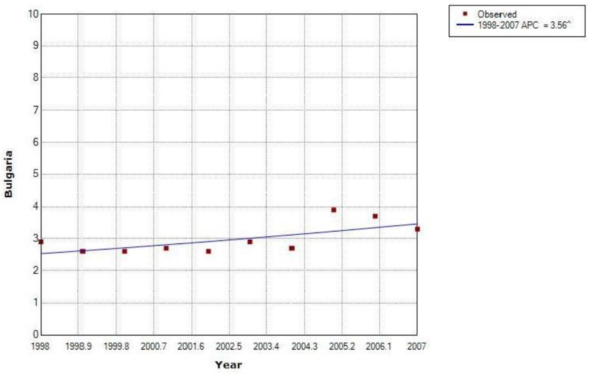

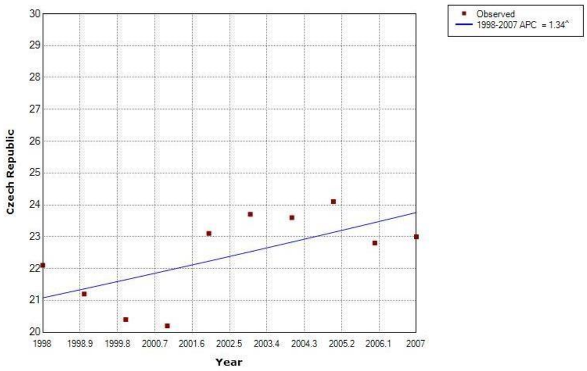

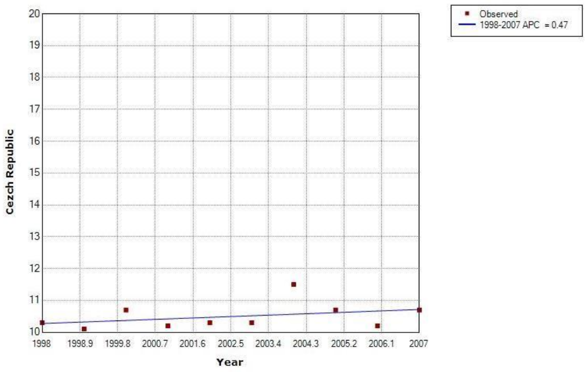

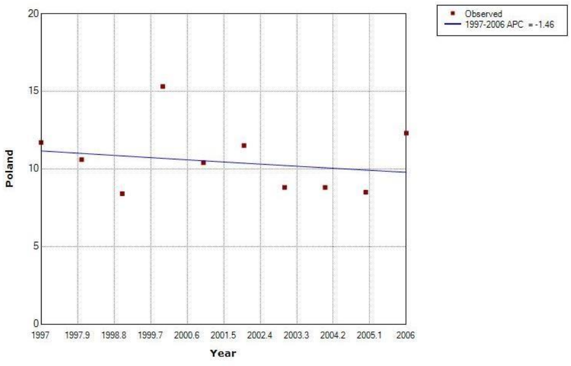

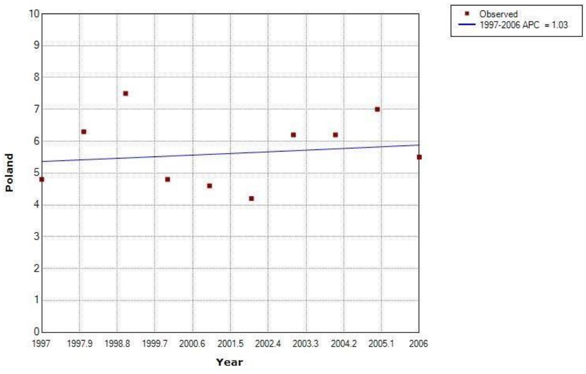

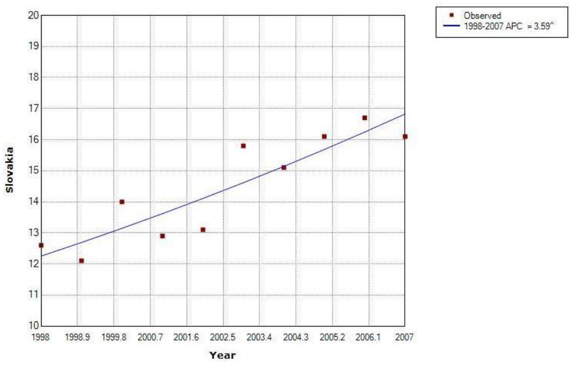

Observed
1998-2007 APC = 3.59^
Slovakia
Year

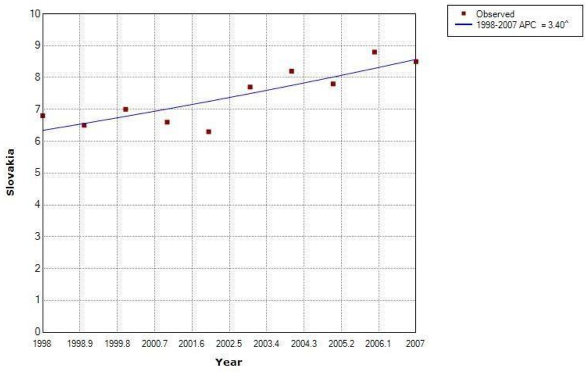

Observed
1998-2007 APC = 3.40^
Slovakia
Year

Supplementary Figure 3 Findings from the joinpoint regression analysis of the global mortality rates of kidney cancer (Left: Male, Right: Female)

1) North America

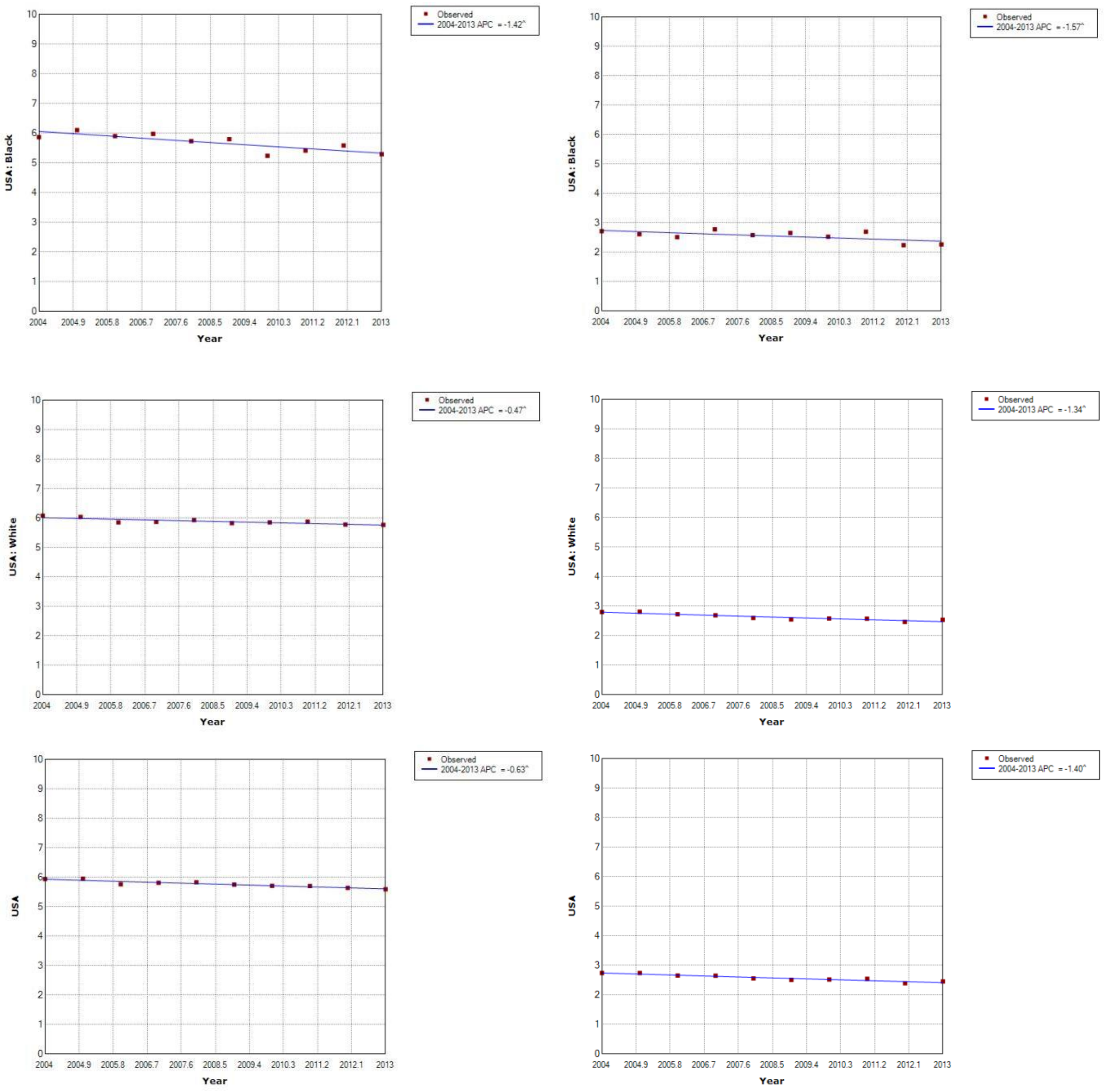

2) Asia

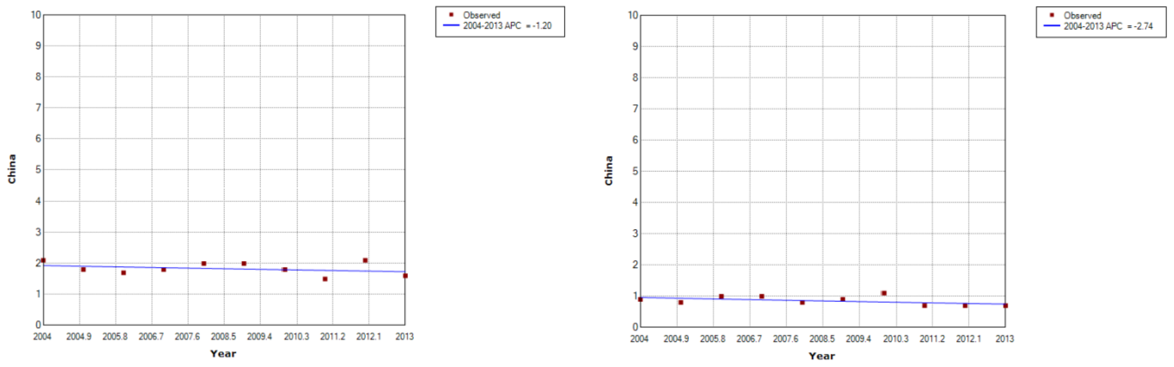

3) Oceania

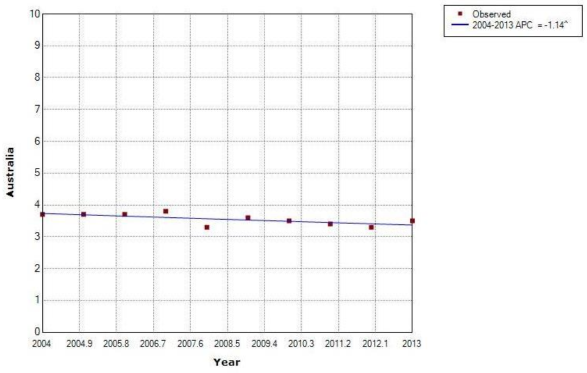

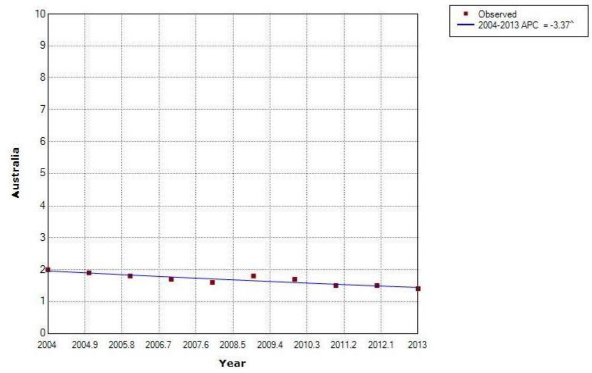

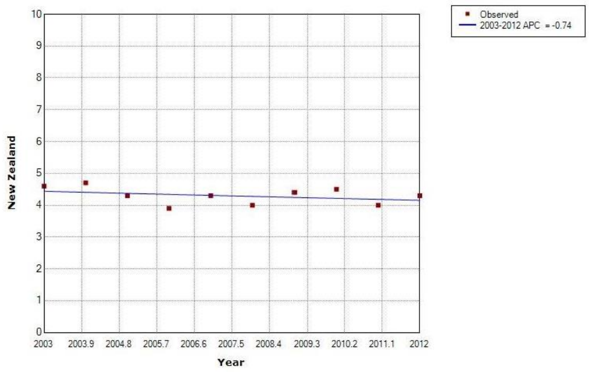

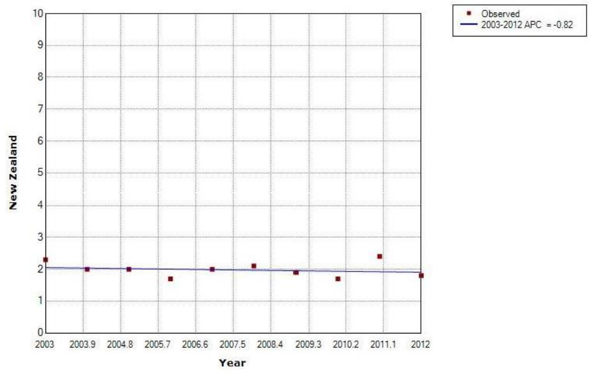

4) Northern Europe

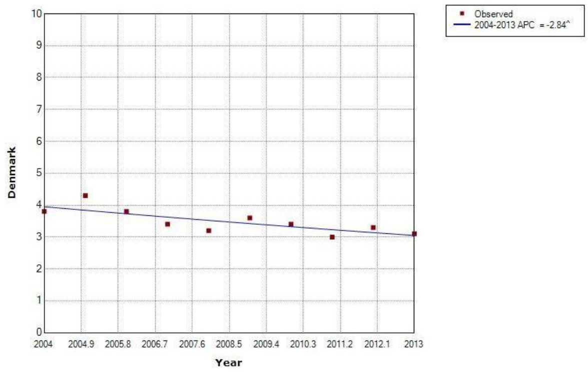

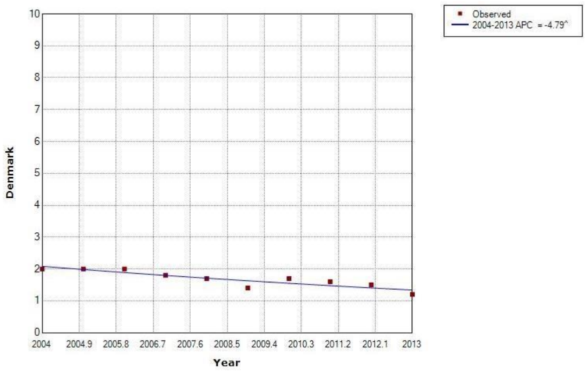

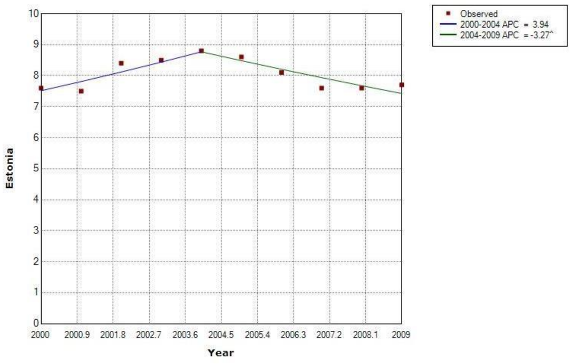

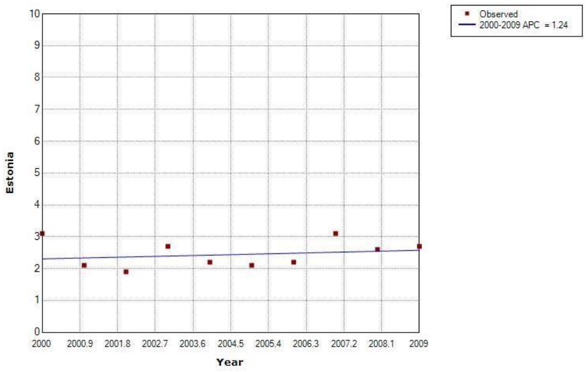

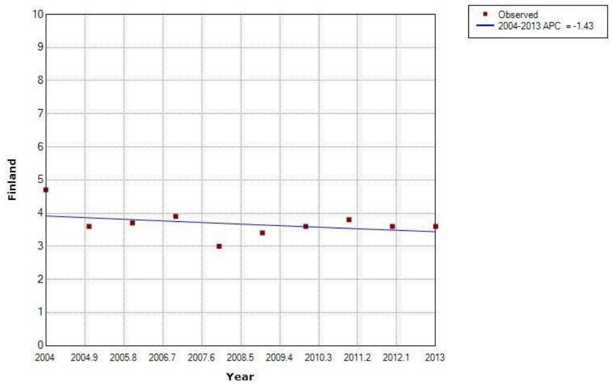
Finland
Year
Observed
2004-2013 APC = -1.43

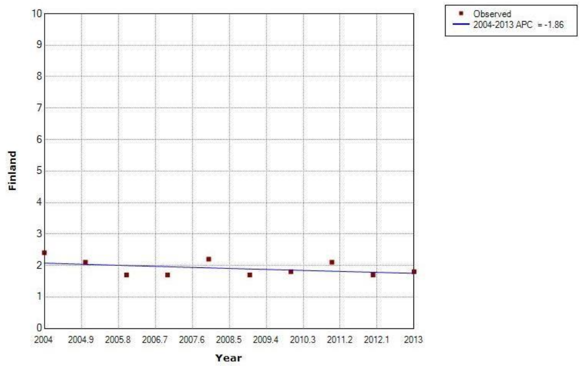
Finland
Year
Observed
2004-2013 APC = -1.86

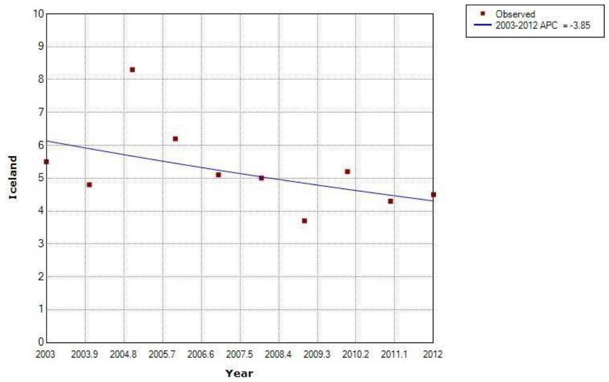
Iceland
Year
Observed
2003-2012 APC = -3.85

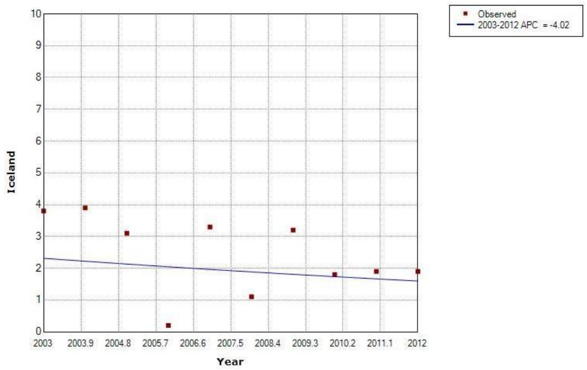
Iceland
Year
Observed
2003-2012 APC = -4.02

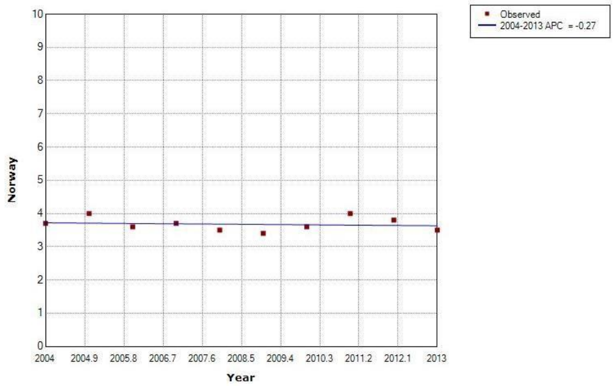
Norway
Year
Observed
2004-2013 APC = -0.27

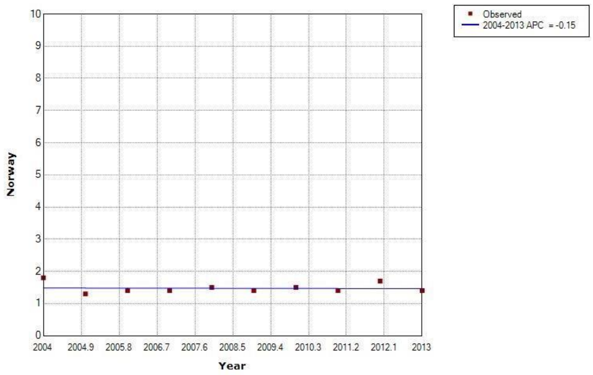
Norway
Year
Observed
2004-2013 APC = -0.15

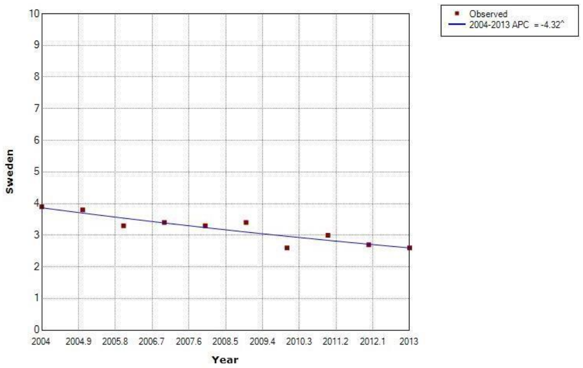
Sweden
Year
Observed
2004-2013 APC = -4.32^

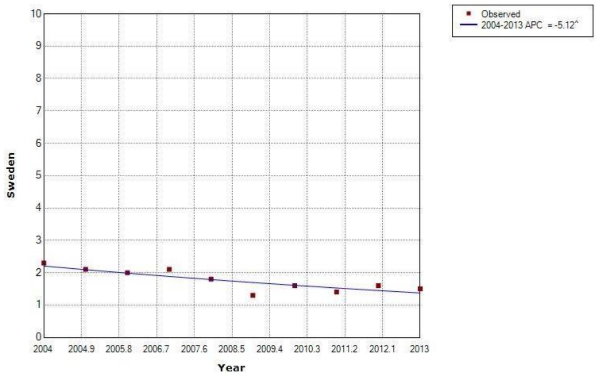
Sweden
Year
Observed
2004-2013 APC = -5.12^

5) Western Europe

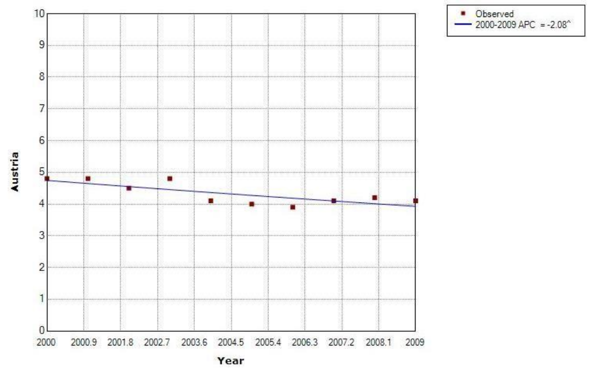

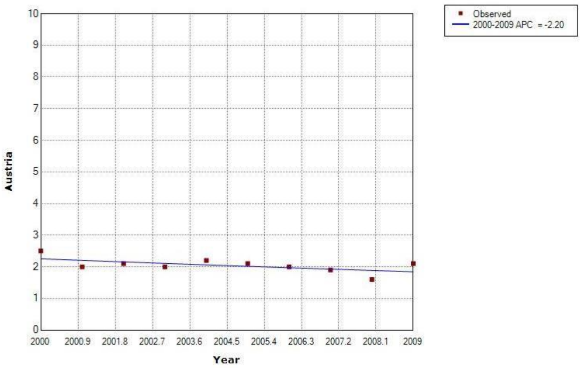

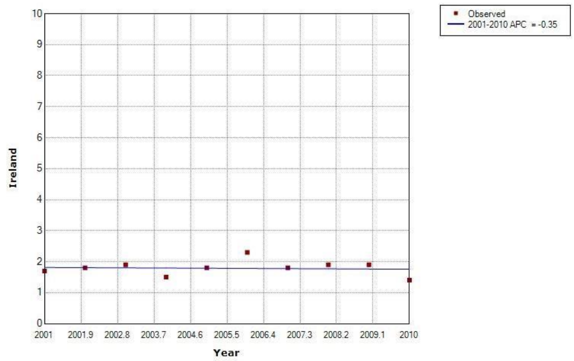

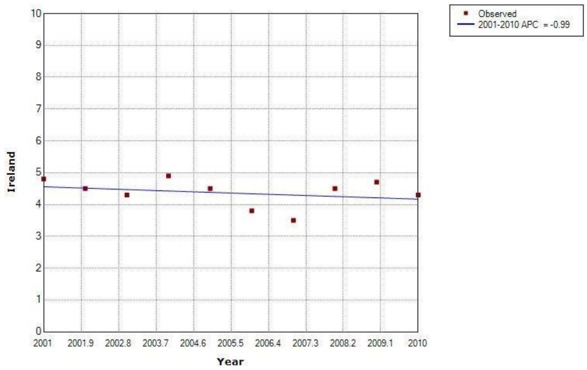

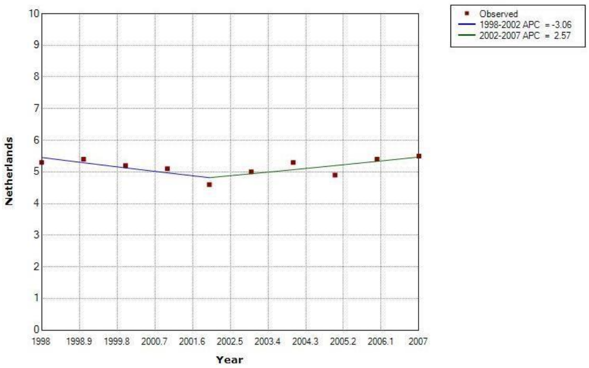

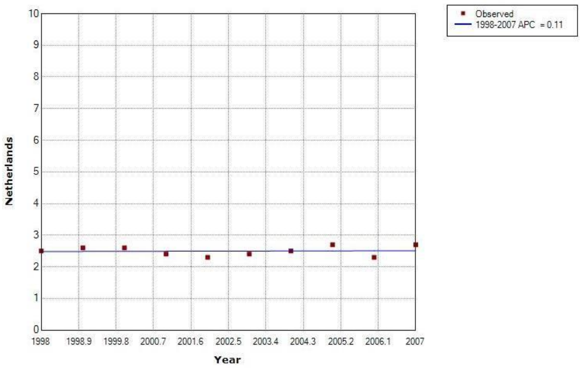

6) Southern Europe

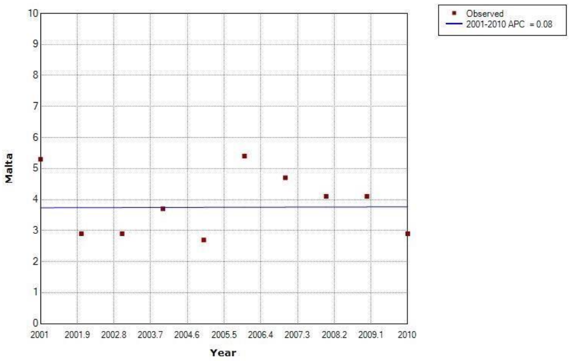

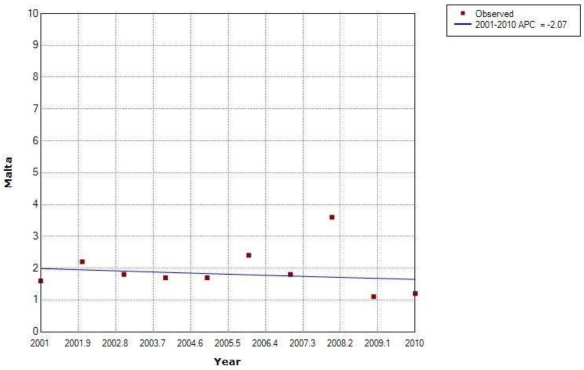

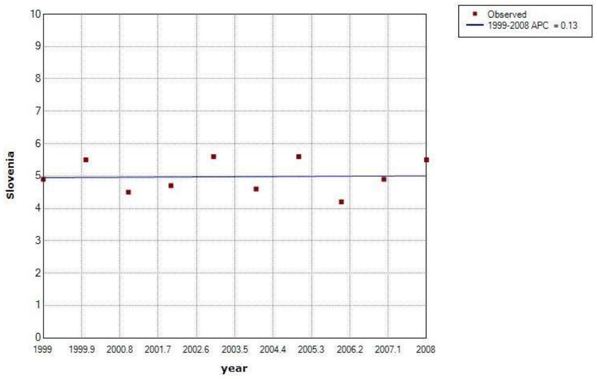

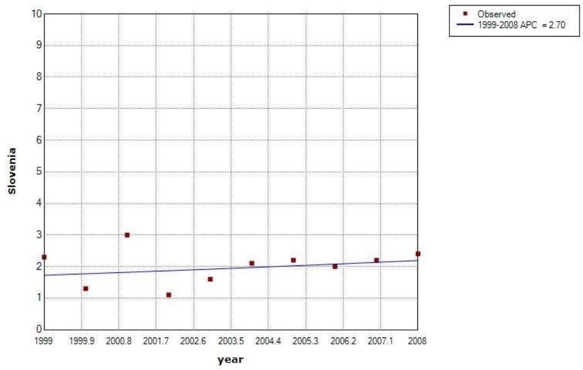

7) Eastern Europe

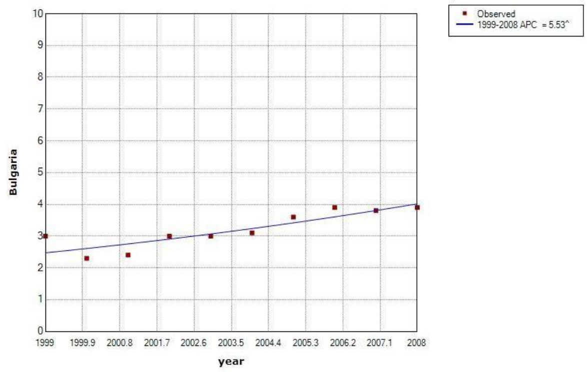

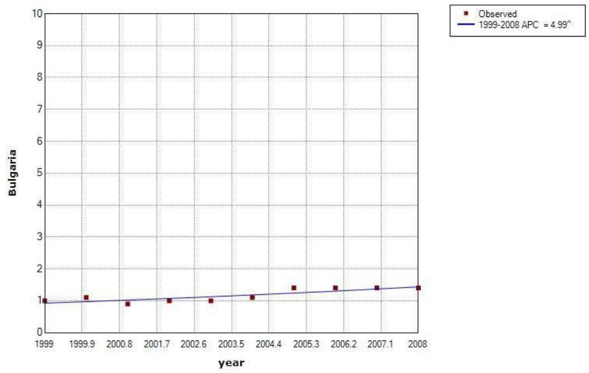

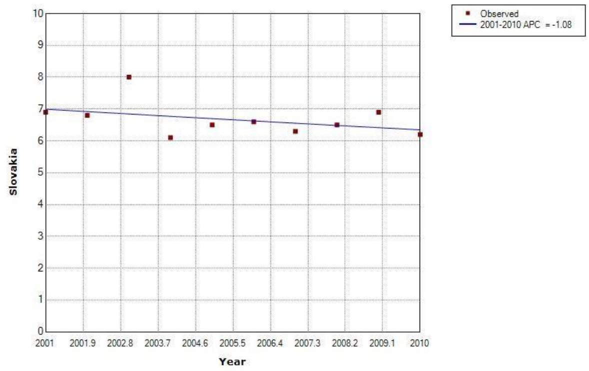

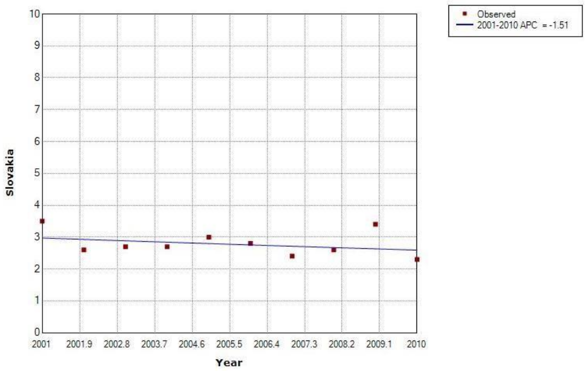

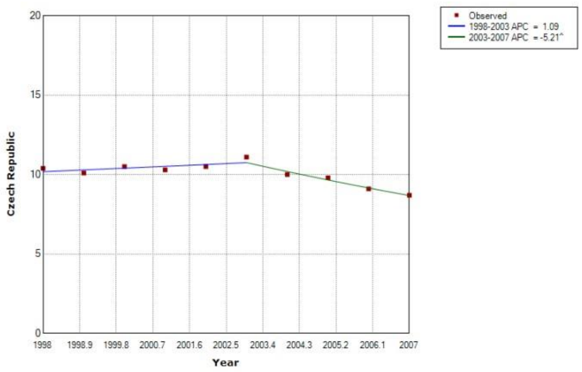

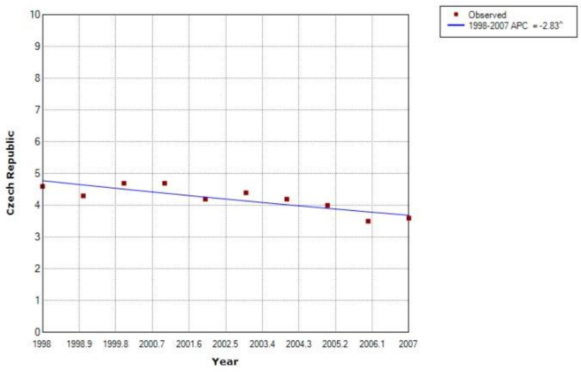

Supplementary Figure 4 Annual Percent Change (APC)-based predicted incidence and mortality rates of kidney cancer compared with the latest available rates (left: men; right: women)

(A). Latin America, the Caribbean and Northern America

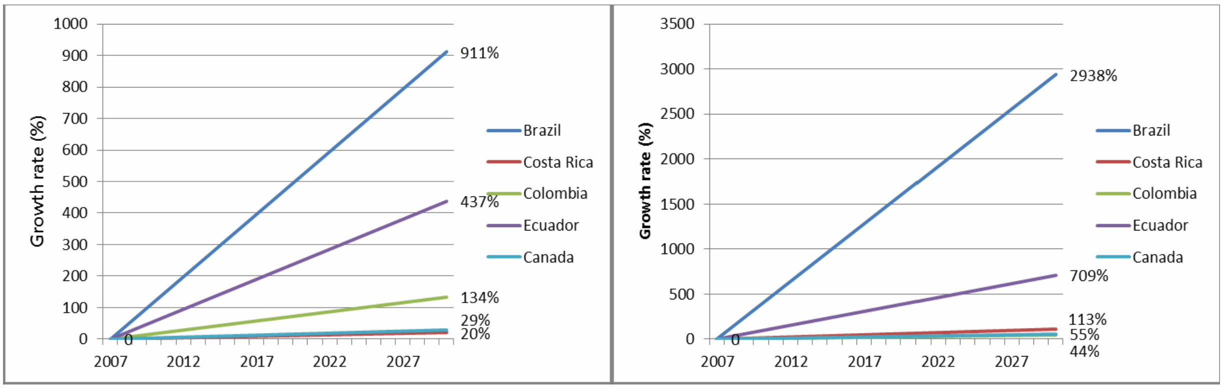

(B). Asia

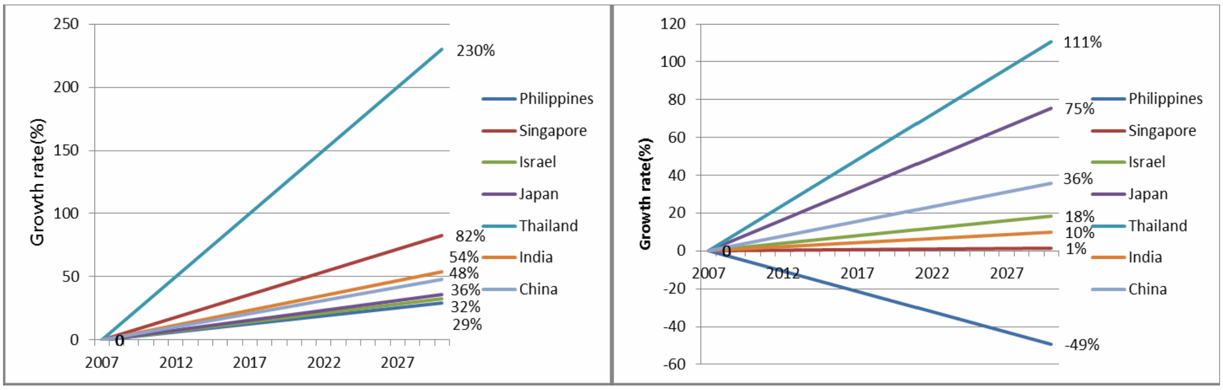

(C). Oceania

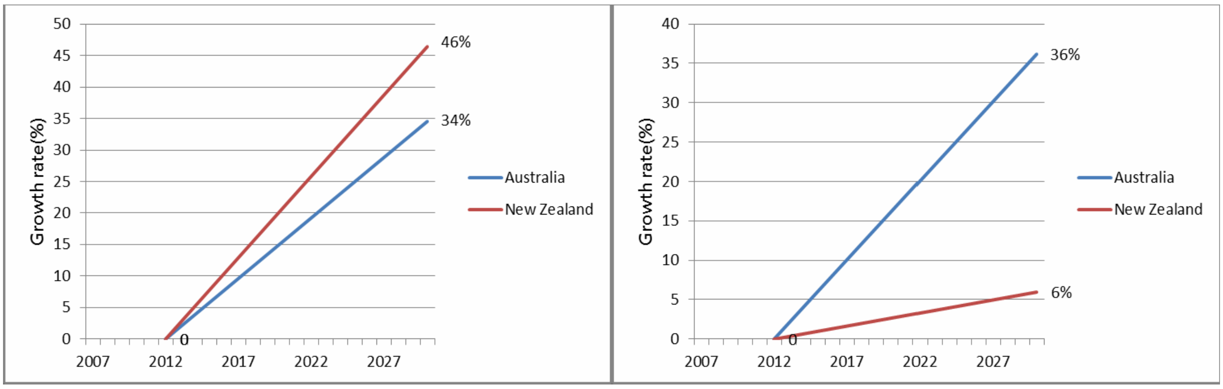

(D). Northern Europe

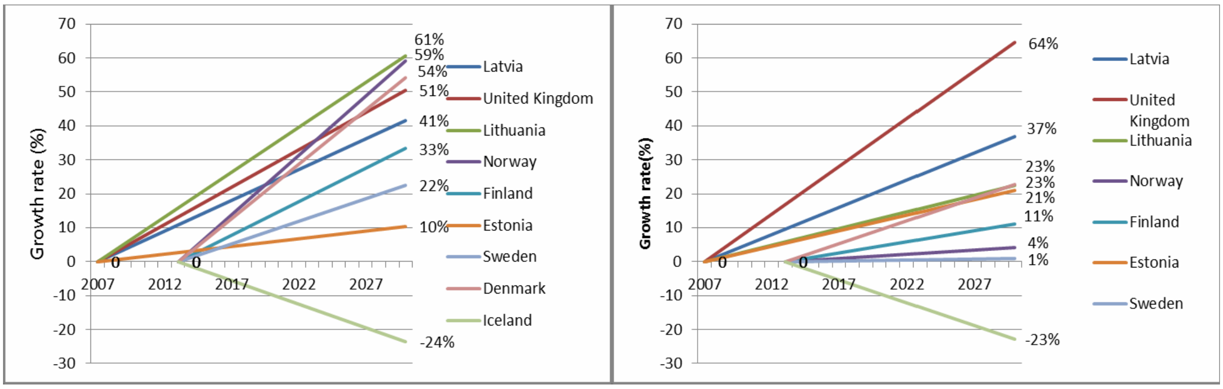

(E). Western Europe

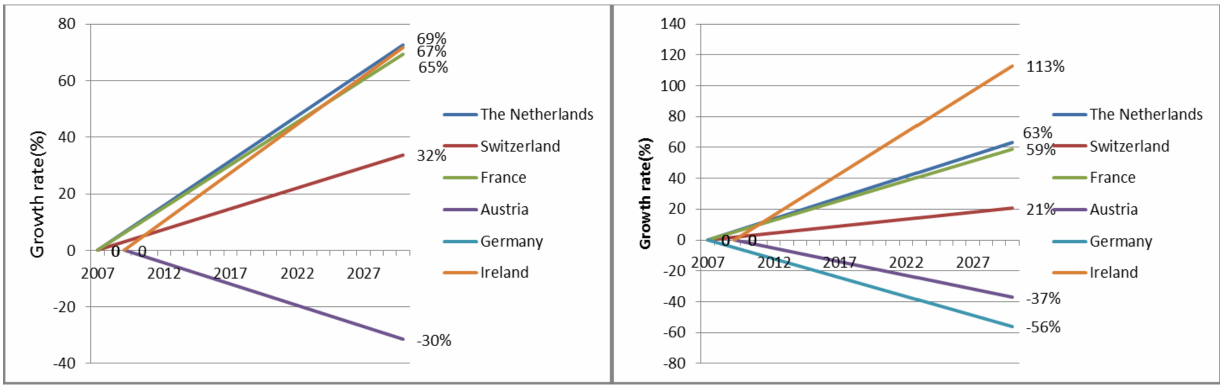

(F). Southern Europe

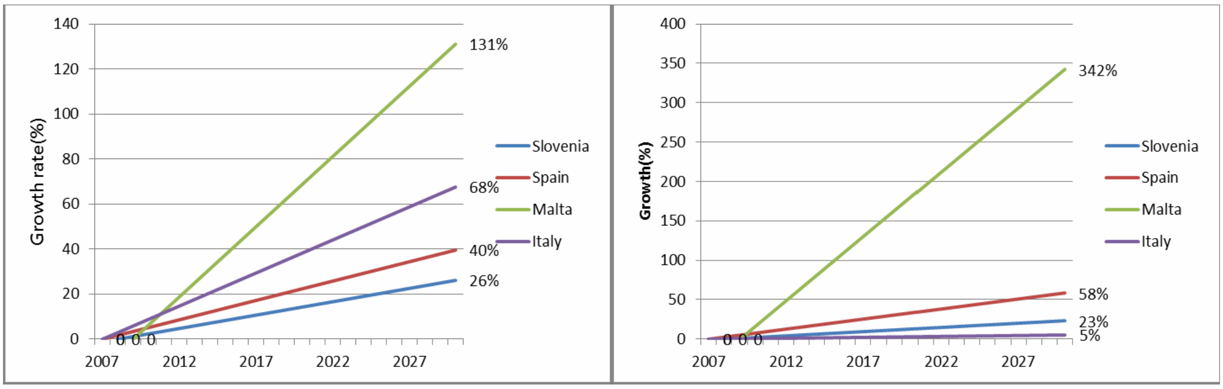

(G). Eastern Europe

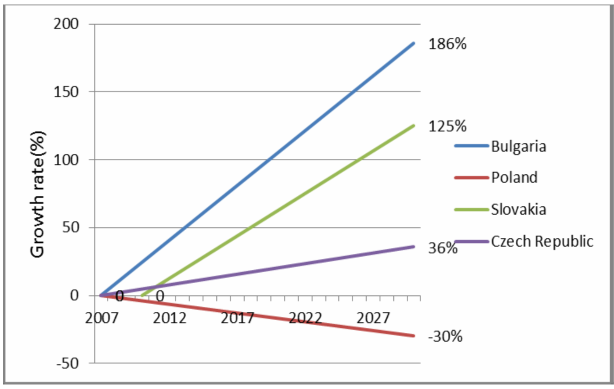

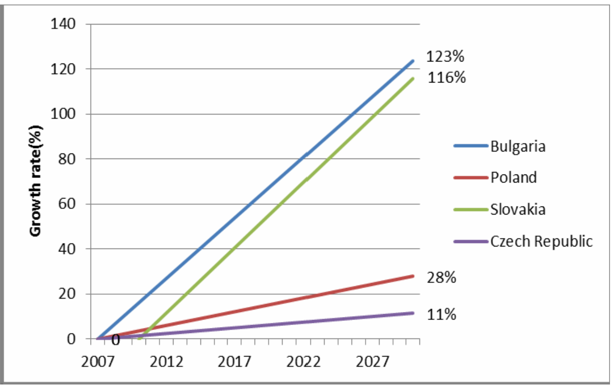

Supplementary Figure 5 **Annual Percent Change (APC)-based predicted mortality rates of kidney cancer compared with the latest available rates (left: men; right: women)**

(A). Asia & Oceania

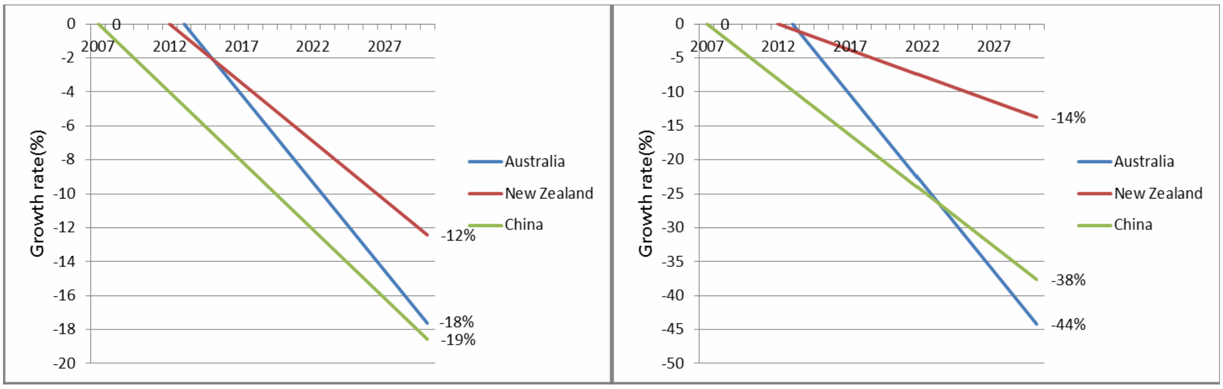

(B). Northern Europe

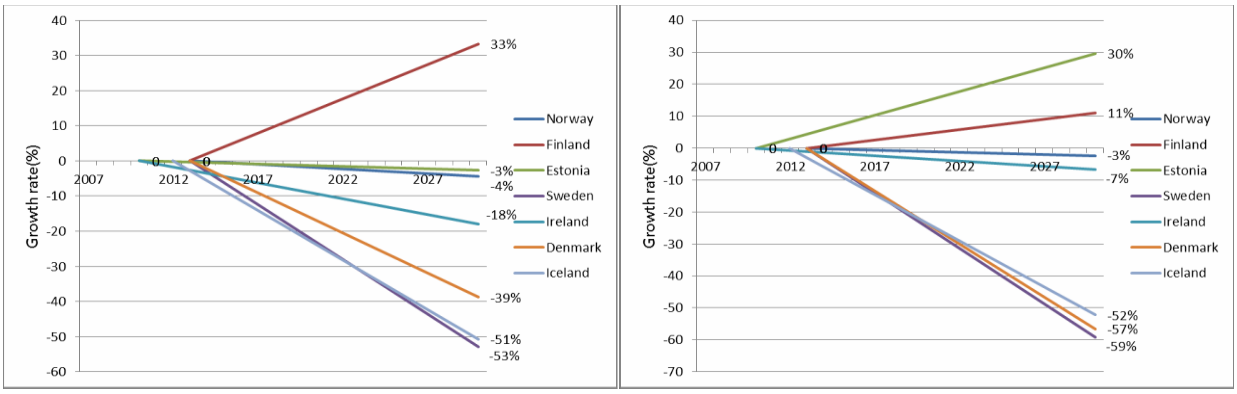

(C). Western Europe

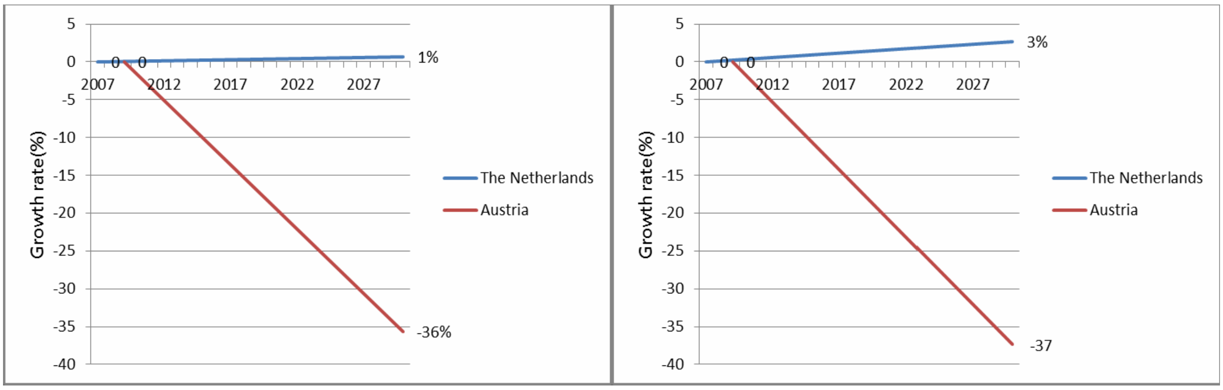

(D). Southern Europe

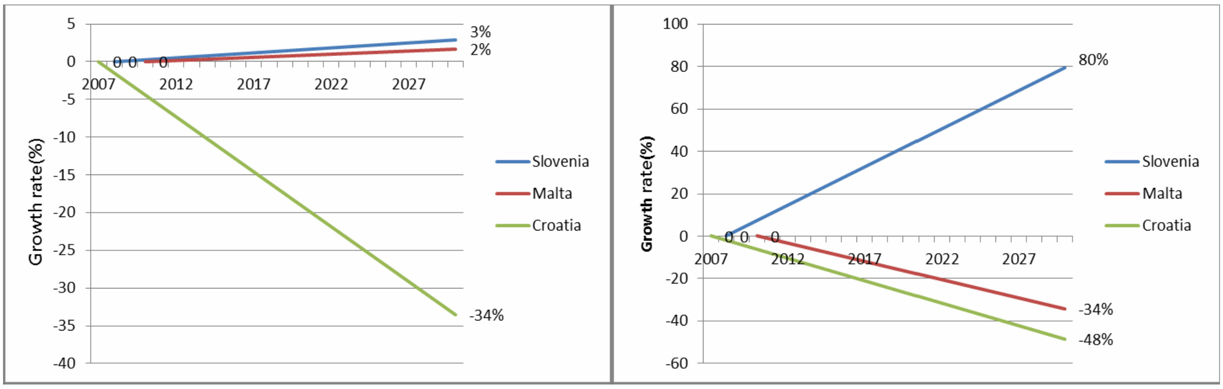

(E). Eastern Europe

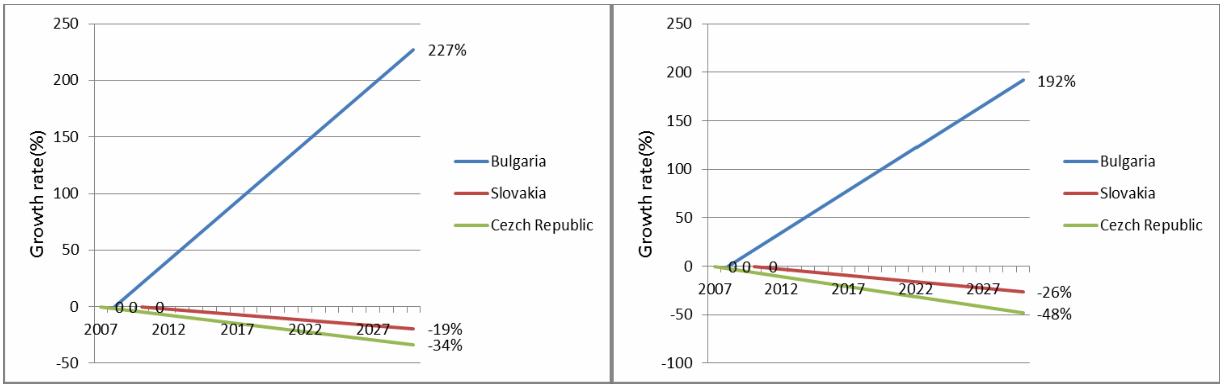

Supplement: Supplementary file 1 — Supplementary Figures [file 41598_2017_15922_MOESM1_ESM.pdf]
